# ACMGA: a reference-free multiple-genome alignment pipeline for plant species

**DOI:** 10.1186/s12864-024-10430-y

**Published:** 2024-05-25

**Authors:** Huafeng Zhou, Xiaoquan Su, Baoxing Song

**Affiliations:** 1 College of Computer Science and Technology, Qingdao University, Qingdao, Shandong 266071 China; 2 National Key Laboratory of Wheat Improvement, Peking University Institute of Advanced Agricultural Sciences, Shandong Laboratory of Advanced Agriculture Sciences in Weifang, Weifang, Shandong 261325 China; 3 Key Laboratory of Maize Biology and Genetic Breeding in Arid Area of Northwest Region of the Ministry of Agriculture, College of Agronomy, Northwest A&F University, Yangling, Shaanxi 712100 China

**Keywords:** Multiple genome alignment, Genome comparison, Plant genome

## Abstract

**Background:**

The short-read whole-genome sequencing (WGS) approach has been widely applied to investigate the genomic variation in the natural populations of many plant species. With the rapid advancements in long-read sequencing and genome assembly technologies, high-quality genome sequences are available for a group of varieties for many plant species. These genome sequences are expected to help researchers comprehensively investigate any type of genomic variants that are missed by the WGS technology. However, multiple genome alignment (MGA) tools designed by the human genome research community might be unsuitable for plant genomes.

**Results:**

To fill this gap, we developed the AnchorWave-Cactus Multiple Genome Alignment (ACMGA) pipeline, which improved the alignment of repeat elements and could identify long (> 50 bp) deletions or insertions (INDELs). We conducted MGA using ACMGA and Cactus for 8 Arabidopsis (*Arabidopsis thaliana*) and 26 Maize (*Zea mays*) de novo assembled genome sequences and compared them with the previously published short-read variant calling results. MGA identified more single nucleotide variants (SNVs) and long INDELs than did previously published WGS variant callings. Additionally, ACMGA detected significantly more SNVs and long INDELs in repetitive regions and the whole genome than did Cactus. Compared with the results of Cactus, the results of ACMGA were more similar to the previously published variants called using short-read. These two MGA pipelines identified numerous multi-allelic variants that were missed by the WGS variant calling pipeline.

**Conclusions:**

Aligning *de* *novo* assembled genome sequences could identify more SNVs and INDELs than mapping short-read. ACMGA combines the advantages of AnchorWave and Cactus and offers a practical solution for plant MGA by integrating global alignment, a 2-piece-affine-gap cost strategy, and the progressive MGA algorithm.

**Supplementary Information:**

The online version contains supplementary material available at 10.1186/s12864-024-10430-y.

## Background

Genomic variation is the basis for the developmental or phenotypical diversity of different organisms, and the identification of genomic variants is of broad interest. Short-read whole-genome sequencing (WGS) has been widely used to call variants in different natural varieties from the same species and represent the variants as single nucleotide variants (SNVs) and insertions or deletions (INDELs) [1]. Short-read WGS is cost-effective and uses massively parallel sequencing technologies (e.g., Illumina) to generate short-reads (usually 50 to 300 bases) across the whole genome randomly and computationally aligns the reads to a pre-existing de novo assembled reference genome sequence. Short-read sequencing works well for SNV calling; however, it exhibits a limited ability to genotype long INDELs (by long INDEL, we refer to INDEL > 50 bp herein) [2]. Detecting long variations is crucial; for example, a 1.2-M inversion in *A. thaliana* chromosome 4 suppressed meiotic recombination in Ler and Col-0 hybrids, and this suppression introduced isolated inversion haplotypes into the worldwide population of Arabidopsis [3, 4]. Furthermore, the existence of repetitive regions complicated short-read mapping techniques, where reads originating from one region were often mapped to multiple repetitive regions, referred to as multi-mapped reads. In such cases, the majority of read aligners would report a randomly selected location from the possible mapping locations, consequently leading to a significantly reduced power to identify variants in repeat regions [5]. Compared with short-read WGS, long-read WGS significantly improved the length of reads [6]. Long-read WGS uses long-read mapping tools such as minimap2 [7] to align long reads to the reference genome sequence and uses long-read variant calling tools such as Sniffles2 [8] to call long INDELs. Long-read WGS can greatly improve the identification of long INDELs.

Using well de novo assembled genome sequences, in theory, we could identify all types of genomic variants [9]. In the last decade, improvements in genome sequencing and assembly technologies have allowed the assembly of a group of accessions from the same plant species, for example, Arabidopsis [10], maize [11], and rice [12]. This affordability of large-scale de novo genome assembly paved the way to precisely reveal genetic variations using the whole-genome alignment (WGA) approach. WGA typically only compares two taxa, but because many genetics and evolutionary studies have been improved by sampling multiple taxa, the multiple-genome alignment (MGA) technology is needed. When aligning a divergent sequence to a reference genome sequence, multiple alignment isomorphs frequently occur, where the essentially same sequence is aligned in different ways. MGA is not simply combining a set of pairwise genome alignments but can unify multiple alignment isomorphs [13]. Herein, we restricted our focus to methods that scaled to more than two genomes. The majority of the available MGA algorithms and tools including Mugsy [14], Mavue [15], and TBA [16] were initially developed by the human genome research community and optimized to align mammal genomes, e.g., human, mouse, rat, or chimpanzee. Moreover, there is an unambiguous contrast between the number of MGA approaches developed in the first decade of the 2000s as opposed to the last ten years [17], and these widely mentioned tools were developed before the availability of population-scale de novo genomes and were rarely optimized using real data, especially plant genomes. Compared with animal genomes, plant genomes exhibit distinct features owing to high content and high activity of transposable elements (TEs), causing a high proportion of repetitive elements in the genome sequence and long INDELs among individuals [18]. Moreover, there is higher sequence diversity between plant species. Thus, new approaches are needed to investigate variants in plant populations efficiently [2].

The Progressive Cactus [19] toolkit incorporates a progressive alignment strategy by generating ancestral sequences. Cactus has been used to align the genomes of 600 bird species. Cactus uses the LASTZ software [20] for pairwise genome alignment. LASTZ provides high sensitivity and controls false positives well for mammal genomes, whereas it has not been well optimized for plant genomes with high sequence diversity and enriched with repetitive elements. AnchorWave [21] is a pairwise WGA software developed mainly by the plant community and has been carefully optimized for plant genomes.

To perform MGA and variant calling for plant natural populations, we combined AnchorWave with Cactus and developed a novel pipeline, AnchorWave-Cactus Multiple Genome Alignment (ACMGA). We compared ACMGA with the short-read WGS variant calling pipeline and Cactus in identifying variants for Arabidopsis and maize. ACMGA aligned a larger proportion of genomes and identified more SNVs and INDELs. The MGA methods also suggested that multi-allelic variants were common in plant populations and largely missed by the previous WGS method. ACMGA was optimized to perform reference-free MGA for the natural individuals of plant inner species.

## Implementation

### Overview

**Fig. 1:**
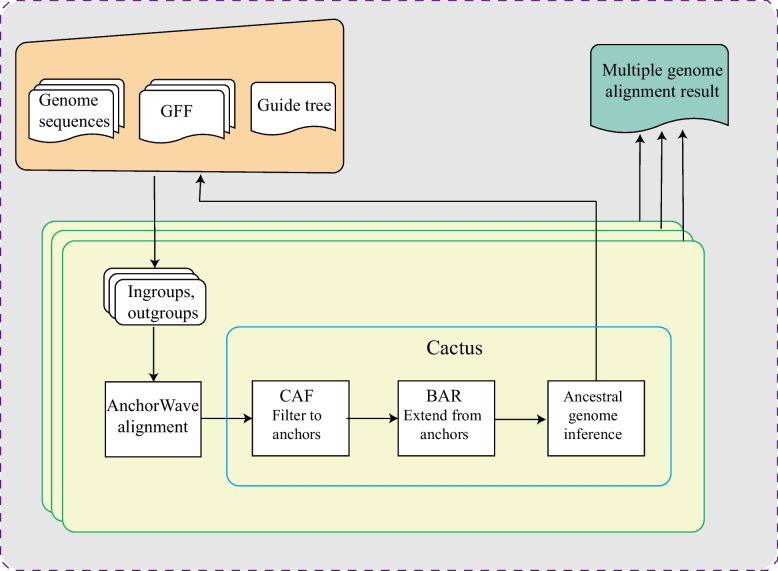
The overall schematic of ACMGA. The flowchart shows the overall flow and the subproblem alignment it proceeds through. The end result is a reconstructed ancestral genome and an alignment between this ancestral genome and its children. Upon the successful resolution of all subproblems, the parent–child alignments are combined into a reference-free MGA result

We developed a reference-free MGA pipeline, ACMGA, to perform MGA for plant de novo assembled genome sequences. The pipeline adapted the progressive strategy [22] implemented in Cactus by breaking a multiple alignment problem into many smaller sub-alignments and using reconstructed ancestral genome sequences for combining these sub-alignments (Fig. 1), each of which aligned only a small number (usually 2–5) of genomes against one another in a pairwise way. ACMGA uses the AnchorWave software to perform pairwise genome alignment. AnchorWave identifies collinear regions via conserved anchors (protein-coding genes) and breaks collinear regions into shorter fragments, i.e., anchor and inter-anchor intervals. By performing global sequence alignment using a 2-piece-affine-gap cost strategy for each shorter interval and merging them, the pairwise genome alignment results were generated in the multiple alignment format (MAF). ACMGA uses the “maf-convert” command of LAST [23], SAMtools [24], and paftools [7] to convert the alignment results from MAF into the SAM and pairwise mApping formats (PAF) [7]. ACMGA uses a custom script (replace_ref_que.py, available from the GitHub repository) and the paf_invert, paf_chain, and paf_tile commands from the Cactus package [25] to fuse the alignment information of the current subtree and feed it into the cactus_consolidated command in the Cactus toolkit (v2.4.0) [25] to reconstruct the ancestral sequence. The reconstructed ancestral sequence is used as input for the next progressive iteration.

AnchorWave requires a genome annotation file in the GFF format for the reference genome. We implemented a pipeline to generate GFF files for constructed ancestral genomes. We combined coding sequences (CDS) from all the input genomes and generated a merged CDS set. For each constructed ancestral genome sequence, we used minimap2 [7] to map the merged CDSs to the ancestral genome sequence and generated a SAM file. Additionally, we used SAMtools [24] and BEDTools [26] to convert the SAM file into BAM and BED formats sequentially, and used the UCSC tools bedToGenePred and genePredToGtf [27] to generate a genome annotation file in the GTF format. We reformatted the GTF file into the GFF format using GFFread [28] and used the generated GFF file together with the ancestral sequence as the input for AnchorWave. The ACMGA pipeline is built upon the Snakemake workflow execution system [29], which ensures robust and scalable execution. Additionally, we provided an ACMGA Docker [30] container and the users only need to download the Docker image and configure the input file.

### Input and output

ACMGA requires a set of FASTA and GFF files of genomes and a guide tree to be aligned. FASTA files are standard results of modern genome assembly projects. The release of almost all high-quality genome sequences is accompanied by the release of GFF files. For the newly assembled genome sequences without annotation, the above-mentioned ancestral genome annotation pipeline can be used. The progressive MGA strategy uses a guide tree to break the MGA process into many pairwise alignment problems. The ACMGA pipeline uses GEAN [31] to extract protein sequences for each individual and uses the OrthoFinder tookit [32] to generate a guide tree. The final output of ACMGA is in the hierarchical alignment (HAL) format [33], which is a graph-based format for storing MGA results. The Cactus toolkit provides many tools to parse HAL files.

## Results

### Genome alignment identifies more SNVs and INDELs than does WGS

We performed MGA for 8 de novo assembled Arabidopsis genome sequences (An-1, C24, Cvi-0, Eri-1, Kyo, L*er*-0, Sha, and Col-0) [10, 34] and 26 genome sequences of maize NAM founder lines [11] using ACMGA and Cactus and compared them with the previously published short-read WGS variant calling results [35, 36]. To compare variant callings obtained from different methods, we artificially introduced a reference genome for each reference-free MGA.

**Fig. 2:**
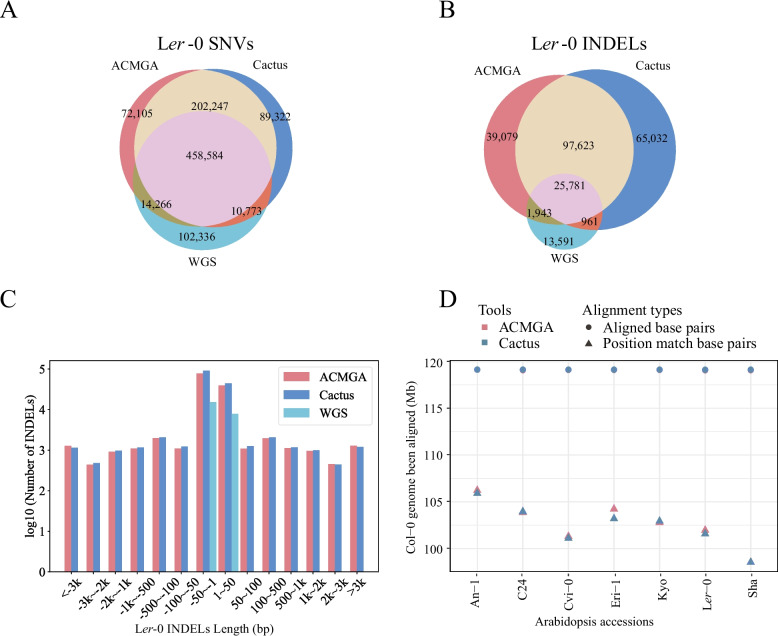
Variant calling of different methods for Arabidopsis (L*er*-0). **A** The SNVs identified between Col-0 and L*er*-0 from the MGA of eight Arabidopsis accessions using ACMGA and Cactus and comparing them with WGS SNVs called by the 1001 genomes project. **B** The INDELs (left alignment standardization) obtained by ACMGA, Cactus, and WGS. **C** The length distribution of INDELs obtained by ACMGA, Cactus, and WGS. **D** The numbers of position matches and aligned base pairs by ACMGA and Cactus to the reference genome (Col-0) across the whole genome

We performed variant calling for seven Arabidopsis accessions using Col-0 as the reference. We found three accessions (An-1, L*er*-0, and Cvi-0) [35] among the seven accessions subjected to short-read WGS-based variant calling via the 1001 genomes project [35]. In the case of Arabidopsis L*er*-0, ACMGA recognized a total of 747,202 SNVs, 164,426 INDELs, and shared 472,850 SNVs and 42,276 INDELs using WGS. Cactus identified a total of 760,926 SNVs, 189,397 INDELs, and shared 469,357 SNVs and 26,742 INDELs using WGS (Figs. 2A and B). The WGS method identified a total of 585,959 SNVs and 42,276 INDELs, which were less than those identified by the WGA methods. Compared with Cactus, ACMGA shared more variants with WGS (Figs. 2A and B). The WGS method only identified INDELs less than 50 bp (Fig. 2C), whereas both MGA methods exhibited the ability to identify long INDELs (> 50 bp). Similar patterns were observed in Cvi-0 and An-1 (Additional file 1: Figs. S1 and S2).

The length of INDELs in CDSs is more often a multiple of three than those in non-CDSs [37]. For variants identified by both ACMGA and Cactus, we observed an enrichment of INDELs with length divisible by three in coding regions. An enrichment pattern was observed for variants identified specifically by ACMGA (Additional file 1: Fig. S3-S8), which was an indication of validation. Compared with Cactus, ACMGA aligned more base pairs as a position match (defined as an ungapped alignment, either matched or mismatched nucleotides, Additional file 1: Fig. S9) in five out of seven accessions and aligned a similar number of base pairs in all Arabidopsis accessions in the whole genome (Fig. 2D).

**Fig. 3:**
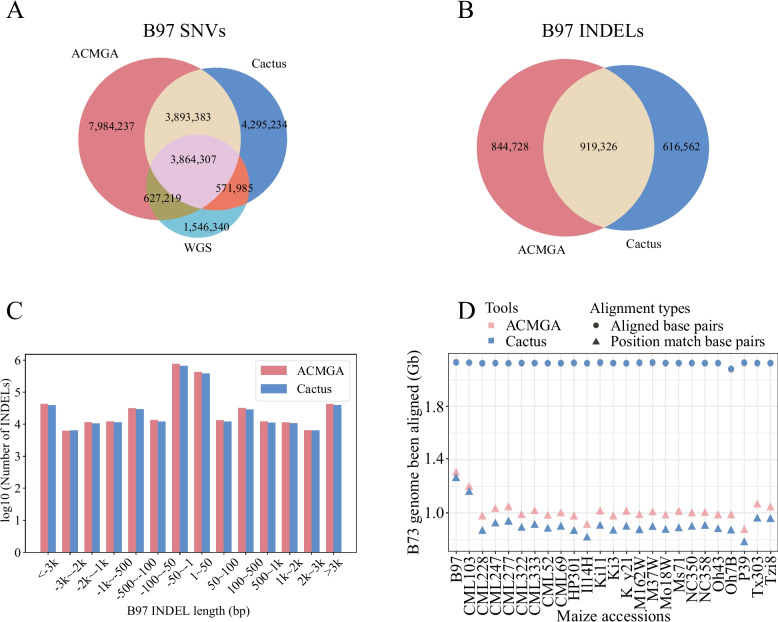
Variant calling of different methods for maize (B97). **A** The SNVs identified between B73 and B97 from the MGA of 26 maize accessions using ACMGA and Cactus and comparing them with WGS SNVs called by the Panzea project. **B** The INDELs (left alignment standardization) obtained by ACMGA, Cactus, and WGS. **C** The length distribution of INDELs obtained by ACMGA, Cactus, and WGS. **D** The numbers of position matches and aligned base pairs by ACMGA and Cactus to the reference genome (B73) across the whole genome

Similarly, for maize, we compared the genome sequence of each accession against B73, resulting in variant callings for 25 accessions. We extracted the short-read WGS-based variant callings for the 25 accessions from a 282-maize-accession dataset [36]. There were no INDEL variant records in the previously published variant callings in the VCF format, and the INDEL variant calling comparison was conducted between ACMGA and Cactus. Consider B97 as an example. ACMGA identified 16,369,146 SNVs and 1,764,054 INDELs, whereas Cactus identified 12,624,909 SNVs and 1,535,888 INDELs. ACMGA had 4,491,526 SNVs in common with WGS, and Cactus had 4,436,292 SNVs in common with WGS (Fig. 3A and 3B). ACMGA identified the largest number of SNVs and shared more common SNV variant records with WGS than Cactus. Moreover, ACMGA could identify more long INDELs than could Cactus (Fig. 3C). Similar patterns were observed for another 24 maize accessions (Additional file 1: Fig. S10-S33). The INDELs with length divisible by three were enriched in coding regions (Additional file 1: Fig. S34-S83). Compared with Cactus, ACMGA aligned more base pairs as a position match and aligned a similar number of base pairs in all maize accessions in the whole genome (Fig. 3D).

In summary, our findings showed that MGA detected a more comprehensive set of genomic variants than did short-read WGS, especially longer INDELs. ACMGA recalled more variants identified by short-read WGS than Cactus.

### ACMGA aligns more base pairs as a position match in genic regions than does *Cactus*

Genic sequences are generally more conserved than intergenic regions, and there are fewer variants in genic regions. To evaluate the performance of the MGA tools, we counted the position match and aligned base pairs in the CDS and genic regions for 7 Arabidopsis and 25 maize accessions.

**Fig. 4:**
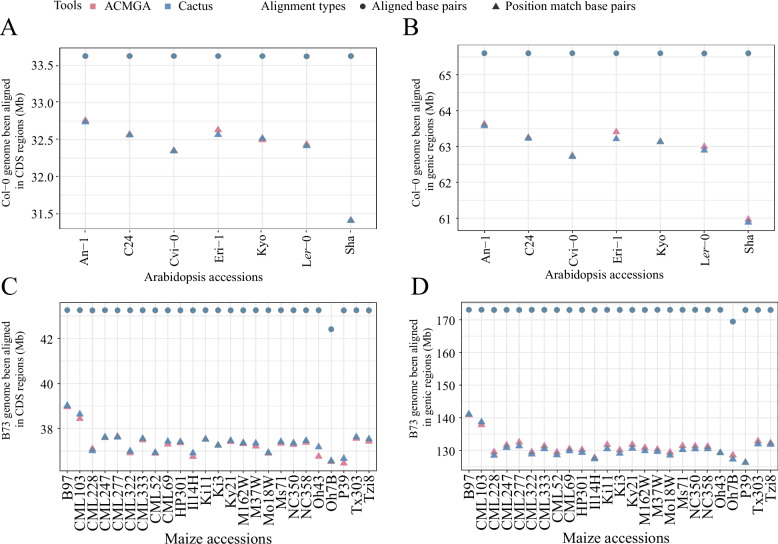
The numbers of position matches and aligned base pairs. **A** The numbers of position matches and aligned base pairs for seven Arabidopsis accessions in the Col-0 CDS region. **B** The numbers of position matches and aligned base pairs for seven Arabidopsis accessions in the Col-0 genic region. **C** The numbers of position matches and aligned base pairs for 25 maize accessions in the B73 CDS region. **D** The numbers of position matches and aligned base pairs for 25 maize accessions in the B73 genic region

In the CDS regions of Arabidopsis, ACMGA aligned more base pairs as a position match in six out of seven accessions and aligned a similar number of base pairs in all Arabidopsis accessions compared with Cactus (Fig. 4A). In the genic regions of Arabidopsis, ACMGA aligned more base pairs as a position match in all accessions and aligned a similar number of base pairs in all accessions compared with Cactus (Fig. 4B).

In the CDS regions of maize, ACMGA aligned slightly fewer base pairs as a position match in most accessions and aligned a similar number of base pairs in all maize accessions compared with Cactus (Fig. 4C). In the genic regions of maize, ACMGA aligned more base pairs as a position match in 23 out of 25 accessions and aligned a similar number of base pairs in all accessions compared with Cactus (Fig. 4D).

### ACMGA detects more SNVs in repetitive sequences than does *Cactus*

**Fig. 5:**
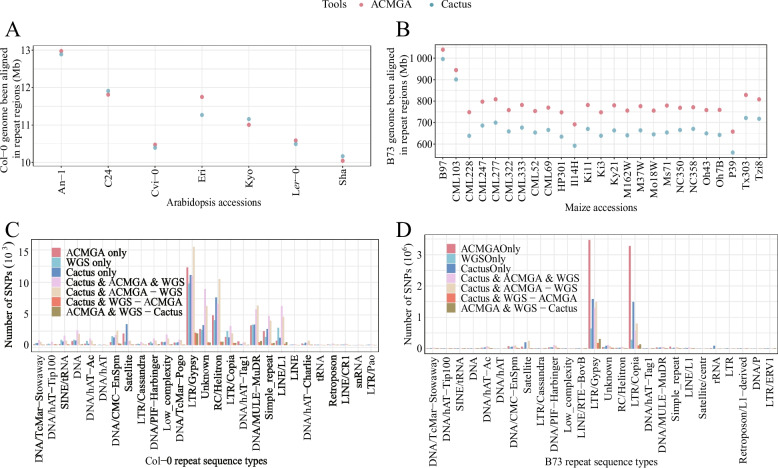
The numbers of position-matched base pairs and SNVs in repeated sequences. **A** The numbers of position-matched base pairs for seven Arabidopsis accessions in the Col-0 repeat region by ACMGA and Cactus. **B** The numbers of position-matched base pairs for 25 maize accessions in the B73 repeat region by ACMGA and Cactus. **C** The number of SNVs originating from repeated sequences between L*er*-0 and Col-0 by different methods. **D** The number of SNVs originating from repeated sequences between B73 and B97 by different methods. The “ACMGA only” category denotes SNVs that are exclusively identifiable by ACMGA, remaining undetected by alternative methodologies. The “WGS only” category denotes SNVs that are exclusively identifiable by WGS, remaining undetected by alternative methodologies. The “Cactus only” category denotes SNVs that are exclusively identifiable by Cactus, remaining undetected by alternative methodologies. The “Cactus&ACMGA&WGS” category denotes SNVs that are commonly identified by all three methods. The “Cactus&ACMGA-WGS” category denotes SNVs discerned by both Cactus and ACMGA, but remain undetected by WGS. The “Cactus&WGS-ACMGA” category denotes SNVs discerned by both ACMGA and WGS, but remain undetected by Cactus

Repetitive sequences pose a major challenge for MGA, and WGS methods also show diminished effectiveness in analyzing these regions. We annotated repetitive sequences for Arabidopsis Col-0 and maize B73 using RepeatMasker [38]. The total length of annotated repetitive elements accounted for 13.12% of the Arabidopsis Col-0 genome assembly, and LTR elements accounted for 6.66%. The annotated repetitive elements accounted for 81.94% of the maize B73 genome assembly, and LTR elements accounted for 74.86%. We also counted the numbers of base pairs aligned as a position match for 7 Arabidopsis and 25 maize accessions aligned to reference repetitive sequence regions. For Arabidopsis, the numbers of position-matched base pairs in repetitive sequences showed no significant difference between ACMGA and Cactus (Fig. 5A). For maize, ACMGA exhibited a significant increase in the number of position-matched base pairs in repetitive sequences compared with Cactus (Fig. 5B).

We further explored the performance of variant calling in repetitive sequences using ACMGA, Cactus, and short-read WGS. In Arabidopsis L*er*-0, ACMGA identified more SNVs from repetitive elements (Fig. 5C) than did Cactus and WGS. In An-1 and Cvi-0, ACMGA and WGS respectively identified the largest number of SNVs from repetitive elements (Additional file 1: Fig S84 and S85). In maize B97, ACMGA identified more SNVs from repetitive elements (Fig. 5D) than did Cactus and WGS. A long terminal repeat (LTR) harbors more variants because it accounts for a very large proportion of repetitive elements. Similar patterns were observed for the remaining 24 maize accessions (Additional file 1: Fig. S86-S109).

### MGA identifies many multi-allelic variants

**Fig. 6:**
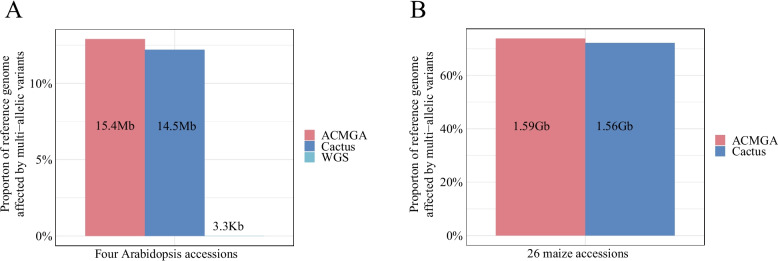
The number of reference genome base pairs affected by multi-allelic variants. **A** The numbers of base pairs and proportion of the genome affected by multi-allelic variants in the reference genome sequence (Col-0) for a population of four Arabidopsis accessions (Col-0, L*er*-0, An-1, and Cvi-0) using ACMGA, Cactus, and WGS. **B** The numbers of base pairs and proportion of the genome affected by multi-allelic variants in the B73 reference sequence genome for a population of 26 maize accessions using ACMGA and Cactus

Multi-allelic variants, many of which have been demonstrated to be functional and disease-relevant [39], have largely been ignored or simplified as biallelic variants. We used Col-0 and B73 as reference genome sequences to count the number of base pairs affected by multi-allelic variants. For four Arabidopsis accessions (Col-0, L*er*-0, An-1, and Cvi-0), ACMGA and Cactus identified 15,355,658 and 14,524,518 base pairs affected by multi-allelic variants, representing 12.88% and 12.19% of the Col-0 genome sequence, respectively. In contrast, WGS methods identified only 3,326 base pairs affected by multi-allelic variants, representing a mere 0.0027% (Fig. 6A). For the 25 maize populations, ACMGA and Cactus identified 1,586,074,982 and 1,555,175,750 base pairs affected by multi-allelic variants, representing 74.40% and 72.95% of the B73 genome sequence, respectively (Fig. 6B). Thus, MGA methods can be significantly effective in identifying multi-allelic variants.

### Computational cost comparison between ACMGA and Cactus

ACMGA uses AnchorWave for pairwise genome alignment. For each ancestral sequence generation iteration, ACMGA runs AnchorWave alignments for five rounds. The comparison data from each iteration are fed into the cactus_consolidated command. Generally, the number of iterations equals the number of accessions, and the total number of AnchorWave alignments can be calculated by (number of accessions − 2) × 5 + 4. On a computer with 128 GB memory and the Intel Xeon W-2295 CPU, Cactus took about 4 h to align eight Arabidopsis genomes, whereas ACMGA took about 5.5 h. For the wall time cost of ACMGA, AnchorWave accounted for approximately 70%, and cactus_consolidated accounted for approximately 30%. The time cost of ACMGA and Cactus was linearly associated with the number of input genome sequences. For each iteration, the computational cost of AnchorWave and LASTZ was squarely associated with genome sequence lengths. The time cost of AnchorWave is also related to genomic sequence diversity and high sequence diversity would cost more computational resources [21]. For large genomes, repeat masking is needed for the Cactus pipeline, and the annotation of repetitive elements (using EDTA [40], for example) would also cost extra computational resources.

## Discussion

### MGA identifies more variants than does short-read WGS

We compared the genomic variations obtained via multiple de novo assembled genome alignment and WGS. Multiple zero-gap de novo assembled genome sequences are being generated. Theoretically, the alignment of assembled sequences can identify all variations, a capability that surpasses what short-read WGS can achieve [9]. Compared with short-read WGS, MGA can identify more variants in repetitive regions, possibly due to read aligners exhibiting a limited ability to accurately map short-reads in repeat regions [5]. Additionally, to call a variant, short reads must be mapped to the reference genome. For highly discordant regions, this reduces SNV calls [41]. When an individual sample lacks read coverage at a specific variant site, this may reflect a structural variation. Short-read WGS often loses this information when imputation is applied to assign a reference allele or alternative allele to the missing site based on linkage disequilibrium [2]. Furthermore, MGA identifies many long INDELs, whereas short-read WGS does not exhibit the ability to identify long INDELs directly. ACMGA identified more long INDELs than did Cactus, possibly due to the pairwise alignment with AnchorWave optimized for the detection of long INDELs compared with LASTZ. For the INDELs specifically identified by ACMGA, we observed INDELs with length divisible by three were enriched in the CDS region, which makes biological sense [37]. Overall, MGA (especially when using ACMGA) reveals a more comprehensive set of genetic variations.

### AnchorWave has been optimized to align complex plant genomes

LASTZ [42] is used in Cactus to perform pairwise genome alignment using the seed-and-extend approach. This approach uses shared* k*-mers as seeds to trigger alignment and then extends the alignment from these shared sequences using dynamic algorithms. To increase sensitivity, LASTZ uses flexible seeds that allow mismatches [23], and it has been adjusted in Cactus to be more sensitive. To increase specificity, repeat elements are generally annotated and soft-masked [43]. If these masked sequences are not used as seeds, the alignment would not be initiated in repeat regions.

ACMGA uses the AnchorWave software to perform pairwise genome alignment. AnchorWave uses the global alignment approach to increase the sensitivity in highly diverse regions and repetitive elements and uses the 2-piece-affine-gap cost strategy to improve the accuracy of long INDEL identification [21].

In maize, ACMGA identified more SNVs and INDELs than Cactus. Additionally, ACMGA has aligned more bases in genic regions, repetitive regions, and across the whole genome relative to Cactus. Compared with maize, the genome size of Arabidopsis is much smaller, and there are fewer long INDELs and repetitive sequences. When applied to Arabidopsis, ACMGA identified fewer variants than Cactus, whereas it shows more overlaps with WGS, indicating enhanced precision. AnchorWave has been optimized to align plant genomes with dispersed repeats, long INDELs, and highly diverse sequences, with ACMGA preserving these attributes.

### MGA can identify more multi-allelic variants

Many population genetics models are built on assumptions of biallelic sites. When more than two alleles are commonly present at a locus, approaches to understanding their evolution become complicated. Meanwhile, some of the observed multi-allelic variants might result from assembly errors. Due to the high prevalence of long INDELs, as well as inversions and translocations in plant genomes, a large proportion of SNVs occur at positions that overlap with those long variants, resulting in multi-allelic variants. As INDELs, inversions, and translocations continue to accumulate, they often happen nestly [44], and nested variants are very common in plants [45]. One of the advantages of genome de novo assembly and MGA over short-read variant calling approaches is the ability to call long and nested variants [46]. Solutions to represent such multi-allelic variants may come from well-designed graph algorithm-based reference-free MGA tools.

### Alignment methods based on graph algorithms are efficient

Graph genomes encode genetic variants as nodes and edges, which preserves the continuity of the sequence and structural variation between individuals. In ACMGA, the cactus_consolidated part of Cactus is used. It uses the Cactus graph as the graph algorithm for MGA [19]. The graph model, due to its ability to handle the complexity of genome-scale sequence alignment, has become a prevalent data structure in numerous MGA tools. Graphs offer a simple method to depict the similarities and differences between genomes, facilitating the visualization and parallel computation of alignments. As the cost of genome assemblies continues to decrease, the importance of the graph data structure for executing efficient and precise MGA on population-scale assemblies will grow, particularly for highly complex plant genomes.

## Methods

### Genome sequences and preprocessing

We obtained the genome sequences of seven de novo assembled Arabidopsis accessions from a previous publication [10] (https://1001genomes.org/data/MPIPZ/MPIPZJiao2020/) and obtained the Col-0 TAIR10 genome assembly from Ensembl [47] (https://plants.ensembl.org/Arabidopsis_thaliana/Info/Index). We downloaded the de novo assembled genome sequences of 25 maize NAM founder lines [11] and B73 v5 from MaizeGDB (https://download.maizegdb.org/).

For Arabidopsis, we obtained WGS variants from the 1001 Genomes Project [35] (https://1001genomes.org/data/GMI-MPI/releases/v3.1/). Regarding maize, we used WGS variants from maize HapMapV3.2.1 [36](http://cbsusrv04.tc.cornell.edu/users/panzea/download.aspx?filegroupid=34). Subsequently, we liftovered the VCF file from AGPv4 coordinates to AGPv5 coordinates using CrossMap (v0.6.5) with a Chain file (https://download.maizegdb.org/Zm-B73-REFERENCE-NAM-5.0/chain_files/).

Additionally, we obtained the TE annotation file of maize B73 from MaizeGDB and converted it into the BED format using GFF2bed [48]. Finally, we applied softmasking to the genomes of 26 maize NAM founder lines using the maskfasta function of BEDTools [26].

### Variant calling from MGA results

The ACMGA pipeline generated an MGA result in the HAL format, the same as Cactus. To compare ACMGA, Cactus, and published short-read WGS-based variant callings, we divided the MGA results into multiple pairwise alignment results. To begin with, we used hal2fasta [33] and faToTwoBit [27] to reformat the reference and query genome sequences into the UCSC two-bit format and used halStats [33] to generate the query genome sequence in the BED format. Next, we used halLiftover [33], with the query genome sequence BED file and the result HAL file as input to create pairwise alignments, which were forced to the positive strand and generate the psl format result with pslPosTarget [27]. These pairwise alignments were then reformatted into chain format using axtChain [27]. Subsequently, we used chain2paf [49] and the paf2maf command of wgatools (https://github.com/wjwei-handsome/wgatools) to convert the chain format into the MAF format. Finally, we used the MAFToGVCF plugin of TASSEL [50] to generate variant calling in the GVCF format. Before comparing variants called by different methods, we used “vt normalize” [51] to normalize INDELs.

### Counting the numbers of aligned base pairs and position match base pairs

To count the number of position matches and aligned base pairs for each accession, we extracted the genome coordinate information of the CDS, genic, and whole-genome wide for Arabidopsis Col-0 and maize B73 and created BED files. Next, all the alignments in the MAF format were reformatted into BAM files using the “maf-convert sam” command of LAST [23] and SAMtools v1.11 [24]. We used the “depth” command of SAMtools to calculate how many base pairs were aligned in the CDS, genic, and whole-genome regions. We used the “samtools depth | awk '$3 > 0{print $0}' | wc -l” command to calculate how many base pairs of the reference genome have a matched position in the query genome.

### Counting multi-allelic variant sites

We compared the number and proportion of reference genome base pairs affected by multi-allelic variants for two reference-free MGA tools and WGS methods separately. For the cases of overlapping with deletions, we counted the cumulative length of these overlapping with deletions (Additional file 1: Fig. S110A) as the number of reference genome base pairs affected by multi-allelic variants. For deletion overlapping with the SNV or insertion, the length of the deletion was counted as the number of reference genome base pairs affected by multi-allelic variants. Each insertion was counted as impacting one base pair of the reference genome (A cartoon explanation can be found in Additional file 1: Fig. S110B and C).

## Availability and requirements

Project name: AnchorWave-Cactus Multiple Genome Alignment.

Project home page: https://github.com/HFzzzzzzz/ACMGA/

Operating system(s): Linux.

Programming languages: Shell, Python, and Snakemake.

Other requirements: Docker and Singularity.

License: MIT.

Any restrictions to use by non-academics: None.

### Supplementary Information

Supplementary Material 1.

## Data Availability

The ACMGA pipeline is available on GitHub at https://github.com/HFzzzzzzz/ACMGA/. Data is provided within the manuscript or supplementary information files.

## Abbreviations

WGS: The whole-genome sequencing
MGA: Multiple genome alignment
ACMGA: AnchorWave-Cactus Multiple Genome Alignment
SNV: Single nucleotide variants
INDEL: Deletions or insertions
MAF: Multiple Alignment Format
SAM: Sequence Alignment/Map Format
BED: Browser Extensible Data
BAM: Binary Alignment Map
PAF: Pairwise mApping Format
CDS: Coding sequence
GVCF: Genomic Variant Call Format
VCF: Variant Call Format
