# Supplementary material for: ACMGA: a reference-free multiple-genome alignment pipeline for plant species
Source: BMC Genomics. 2024 May 25;25:515. doi: 10.1186/s12864-024-10430-y (PMC11127342; doi:10.1186/s12864-024-10430-y)
Supplement: Supplementary file 1 — Supplementary Material 1. [file 12864_2024_10430_MOESM1_ESM.pdf]

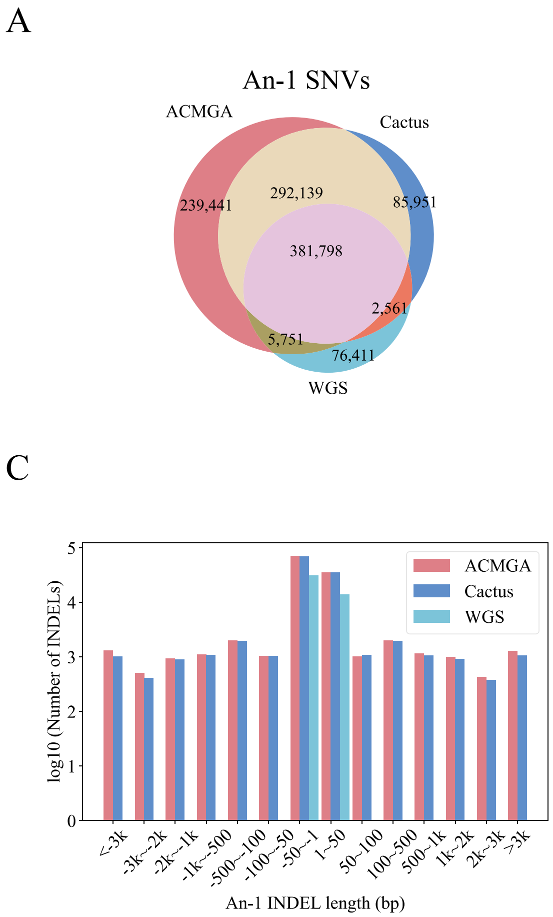

Fig. S1 Variant calling of different methods for Arabidopsis (An-1). (A) The SNVs identified between Col-0 and An-1 from the MGA of eight Arabidopsis accessions using ACMGA and Cactus, and comparing them with WGS SNVs called by the 1001 genomes project. **(B)** The INDELs (left alignment standardization) obtained by ACMGA, Cactus, and WGS. **(C)** The length distribution of INDELs obtained by ACMGA, Cactus, WGS.

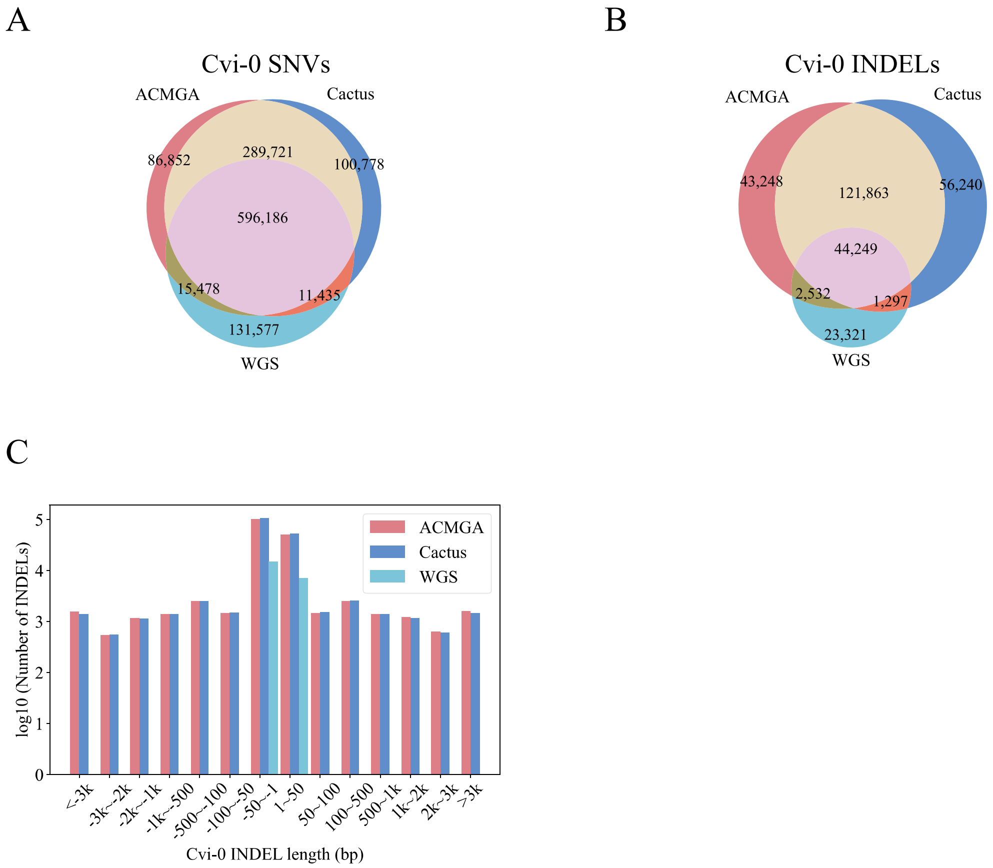

Fig. S2 Variant calling of different methods for Arabidopsis (Cvi-0). (A) The SNVs identified between Col-0 and Cvi-0 from the MGA of eight Arabidopsis accessions using ACMGA and Cactus, and comparing them with WGS SNVs called by the 1001 genomes project. **(B)** The INDELs (left alignment standardization) obtained by ACMGA, Cactus, and WGS. **(C)** The length distribution of INDELs obtained by ACMGA, Cactus, WGS.

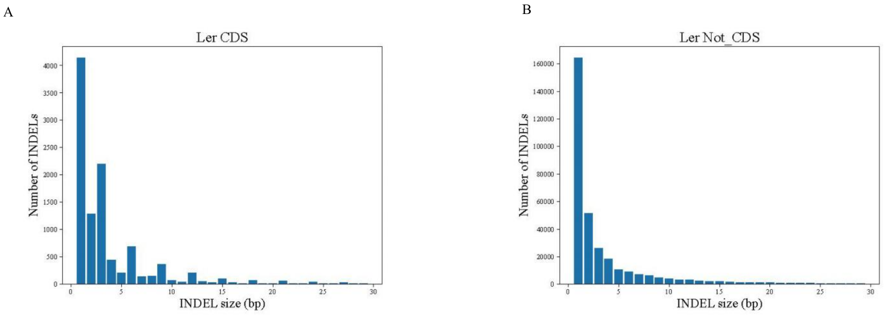

Fig. S3 Length distribution of INDELs identified by ACMGA and Cactus in CDS and Non-CDS regions (L*er-0*). Most frequent INDEL sizes differ between coding and non-coding regions. Multiple of three are much more common in coding sequences.

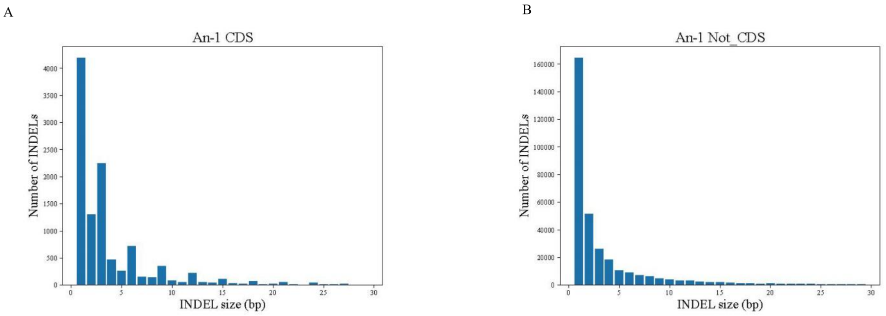

Fig. S4 Length distribution of INDELs identified by ACMGA and Cactus in CDS and Non-CDS regions (An-1). Most frequent INDEL sizes differ between coding and non-coding regions. Multiple of three are much more common in coding sequences.

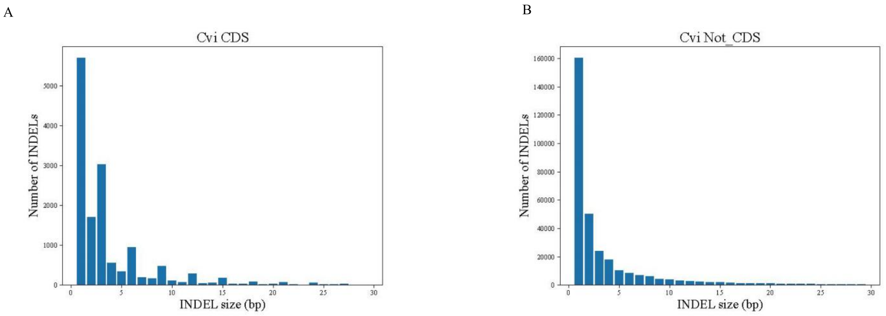

Fig. S5 Length distribution of INDELs identified by ACMGA and Cactus in CDS and Non-CDS regions (Cvi-0). Most frequent INDEL sizes differ between coding and non-coding regions. Multiple of three are much more common in coding sequences.

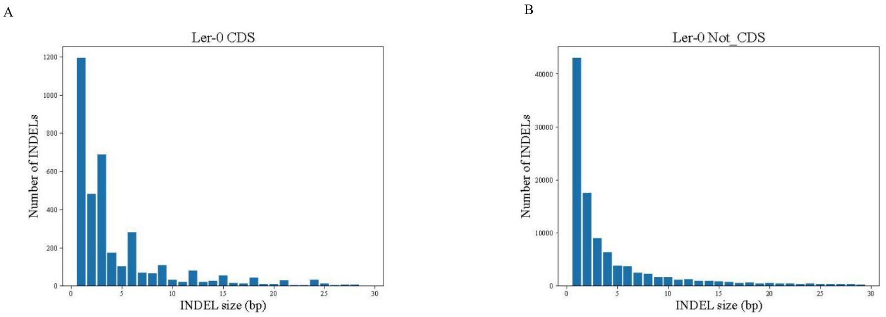

Fig. S6 Length distribution of INDELs identified solely by ACMGA in CDS and Non-CDS regions (L*er-0*). Most frequent INDEL sizes differ between coding and non-coding regions. Multiple of three are much more common in coding sequences.

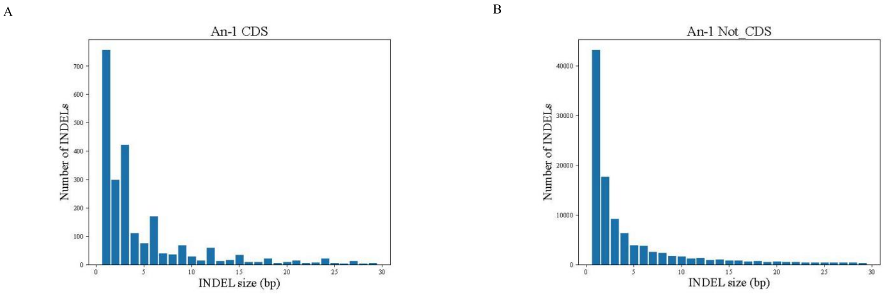

Fig. S7 Length distribution of INDELs identified solely by ACMGA in CDS and Non-CDS regions (An-1). Most frequent INDEL sizes differ between coding and non-coding regions. Multiple of three are much more common in coding sequences.

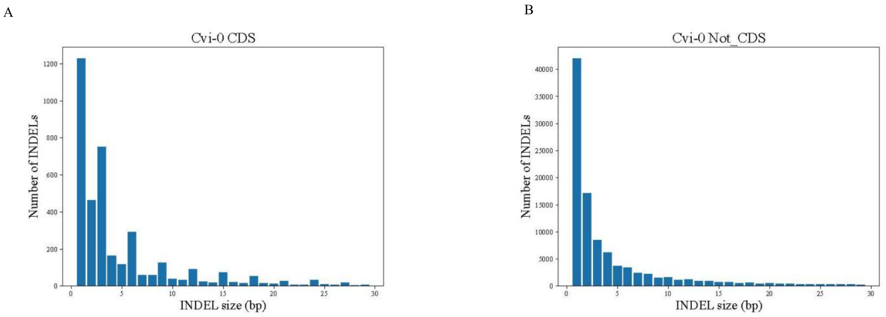

Fig. S8 Length distribution of INDELs identified solely by ACMGA in CDS and Non-CDS regions (Cvi-0). Most frequent INDEL sizes differ between coding and non-coding regions. Multiple of three are much more common in coding sequences.

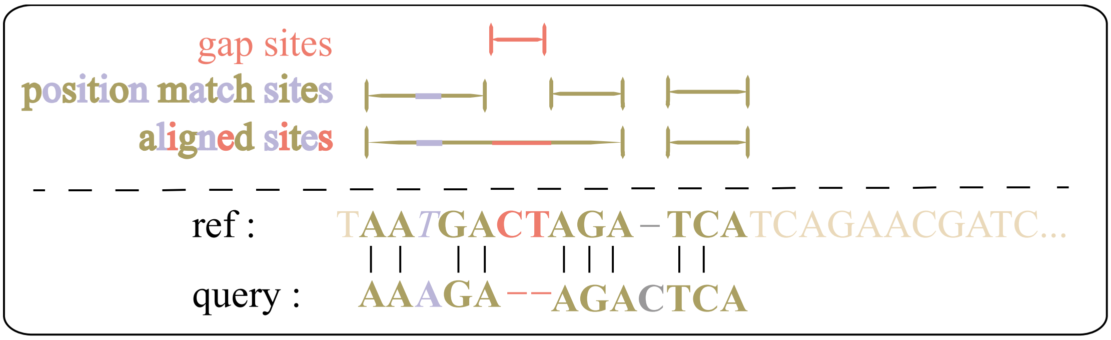

Fig. S9 An example illustrating the concepts of (position) match sites, aligned sites, gapsites of the reference sequence used in this study. The *T* site of the reference sequence(ref) in purple and italic font of the reference sequence is aligned as a nucleotide mismatch, but is a position match.

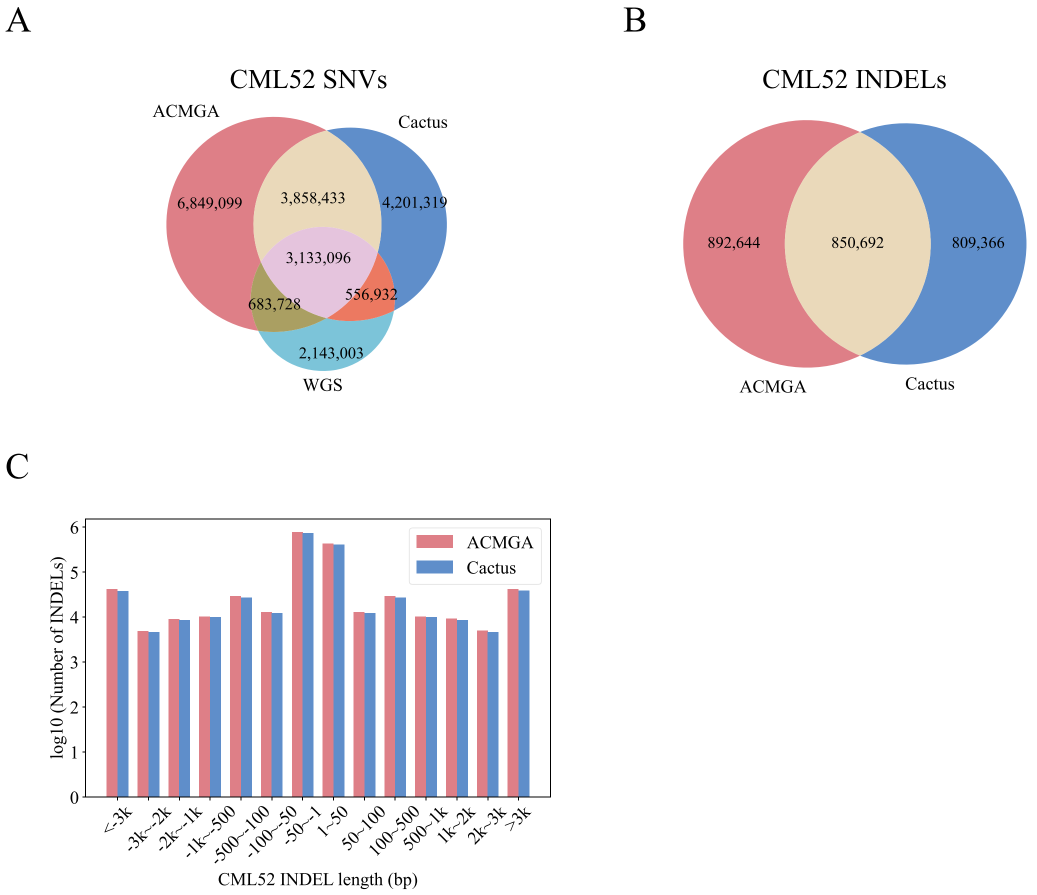

Fig. S10 Variant calling of different methods for maize (CML52). (A) The SNVs identified between B73 and CML52 from the MGA of 26 maize accessions using ACMGA and Cactus, and comparing them with WGS SNVs called by the Panzea project. **(B)** The INDELs (left alignment standardization) obtained by ACMGA, Cactus, and WGS. **(C)** The length distribution of INDELs obtained by ACMGA, Cactus, WGS.

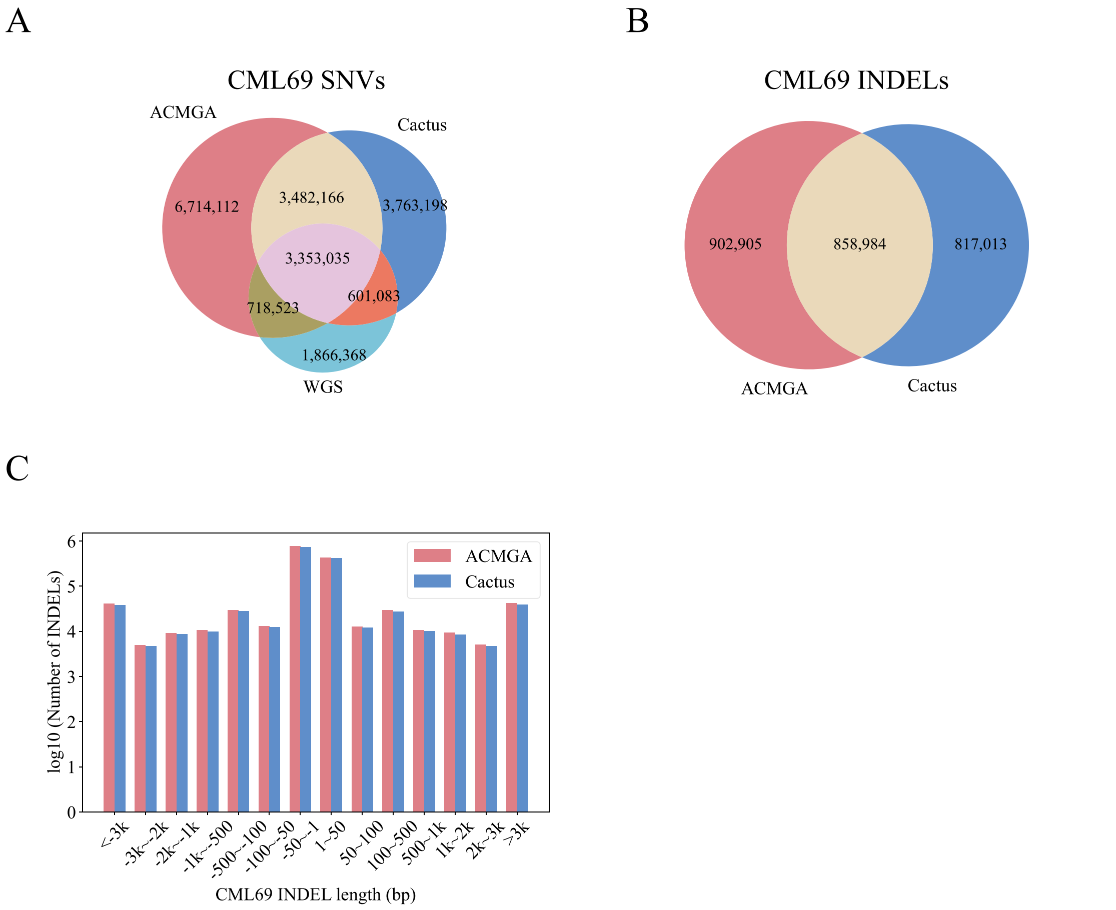

Fig. S11 Variant calling of different methods for maize (CML69). (A) The SNVs identified between B73 and CML69 from the MGA of 26 maize accessions using ACMGA and Cactus, and comparing them with WGS SNVs called by the Panzea project. **(B)** The INDELs (left alignment standardization) obtained by ACMGA, Cactus, and WGS. **(C)** The length distribution of INDELs obtained by ACMGA, Cactus, WGS.

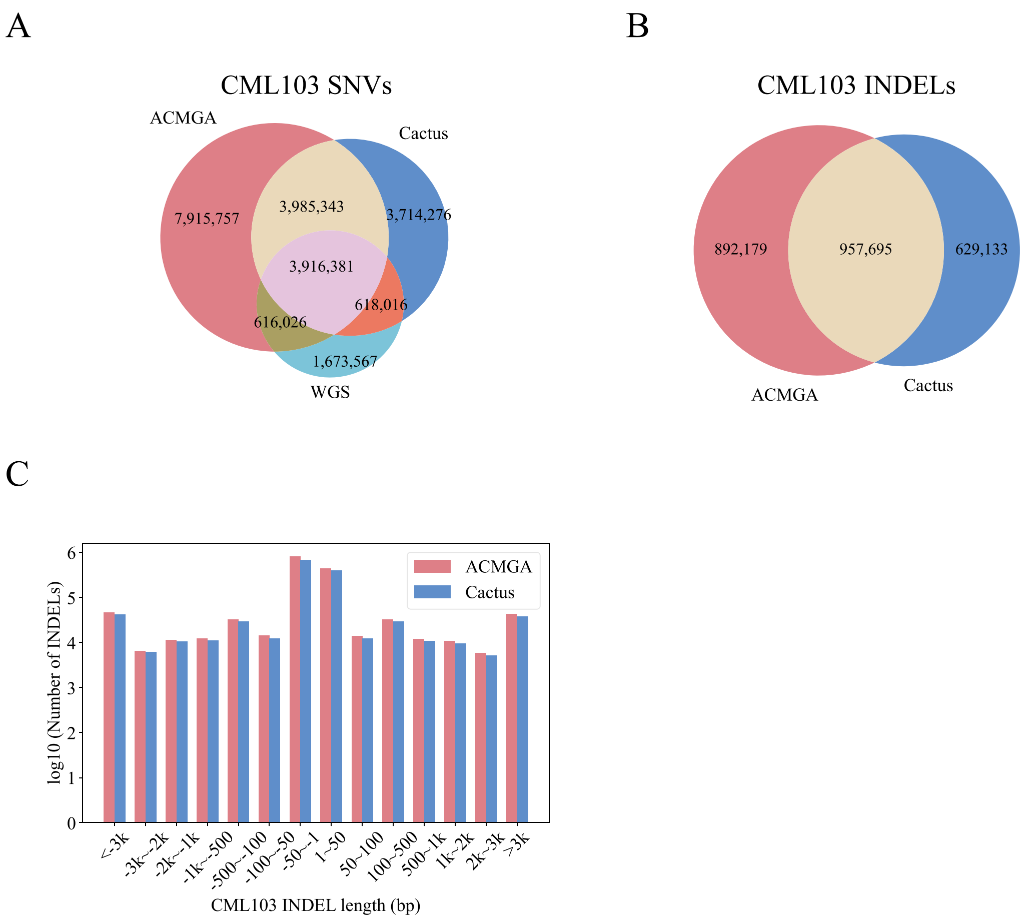

Fig. S12 Variant calling of different methods for maize (CML103). (A) The SNVs identified between B73 and CML103 from the MGA of 26 maize accessions using ACMGA and Cactus, and comparing them with WGS SNVs called by the Panzea project. **(B)** The INDELs (left alignment standardization) obtained by ACMGA, Cactus, and WGS. **(C)** The length distribution of INDELs obtained by ACMGA, Cactus, WGS.

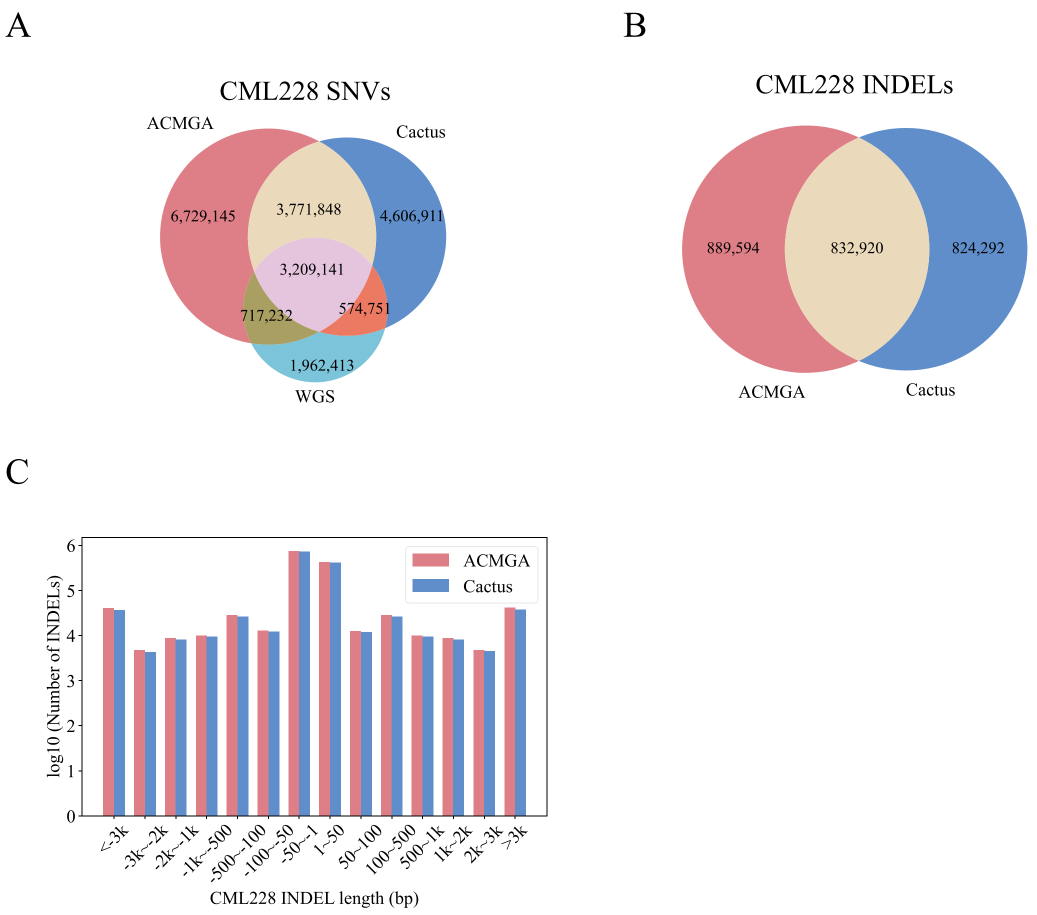

Fig. S13 Variant calling of different methods for maize (CML228). (A) The SNVs identified between B73 and CML228 from the MGA of 26 maize accessions using ACMGA and Cactus, and comparing them with WGS SNVs called by the Panzea project. **(B)** The INDELs (left alignment standardization) obtained by ACMGA, Cactus, and WGS. **(C)** The length distribution of INDELs obtained by ACMGA, Cactus, WGS.

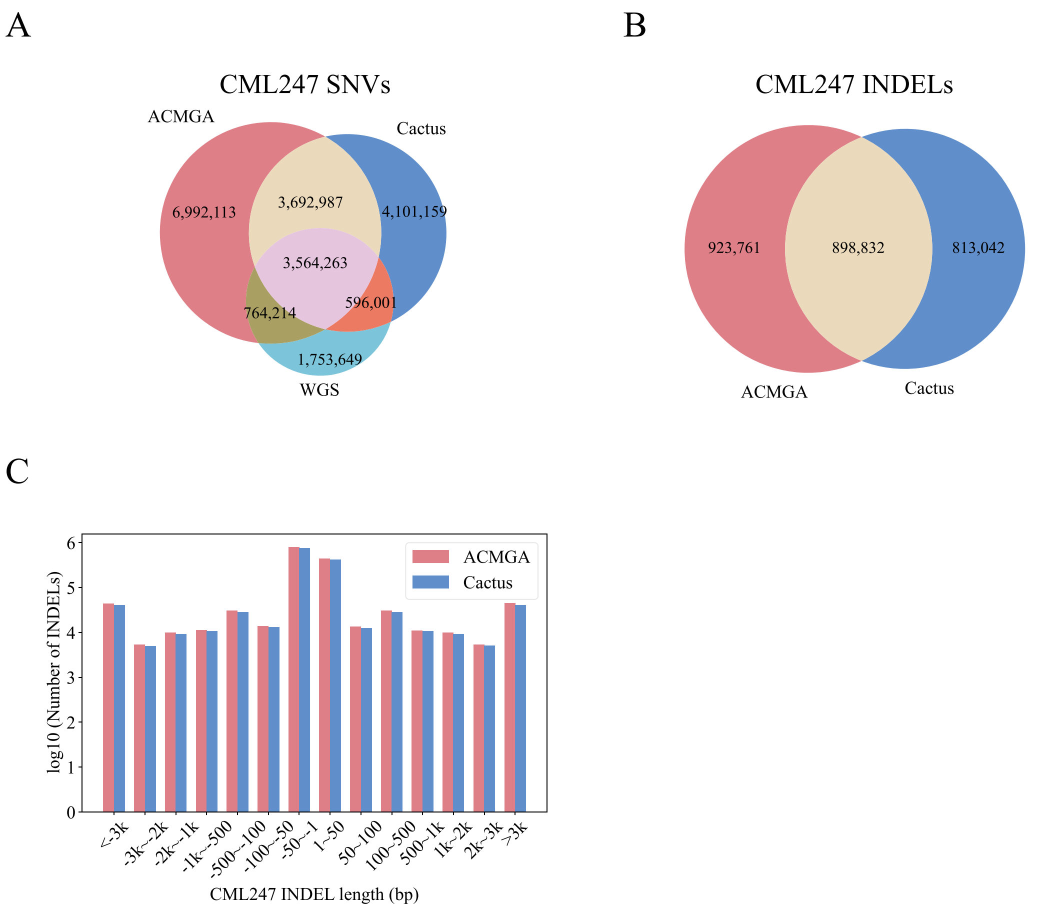

Fig. S14 Variant calling of different methods for maize (CML247). (A) The SNVs identified between B73 and CML247 from the MGA of 26 maize accessions using ACMGA and Cactus, and comparing them with WGS SNVs called by the Panzea project. **(B)** The INDELs (left alignment standardization) obtained by ACMGA, Cactus, and WGS. **(C)** The length distribution of INDELs obtained by ACMGA, Cactus, WGS.

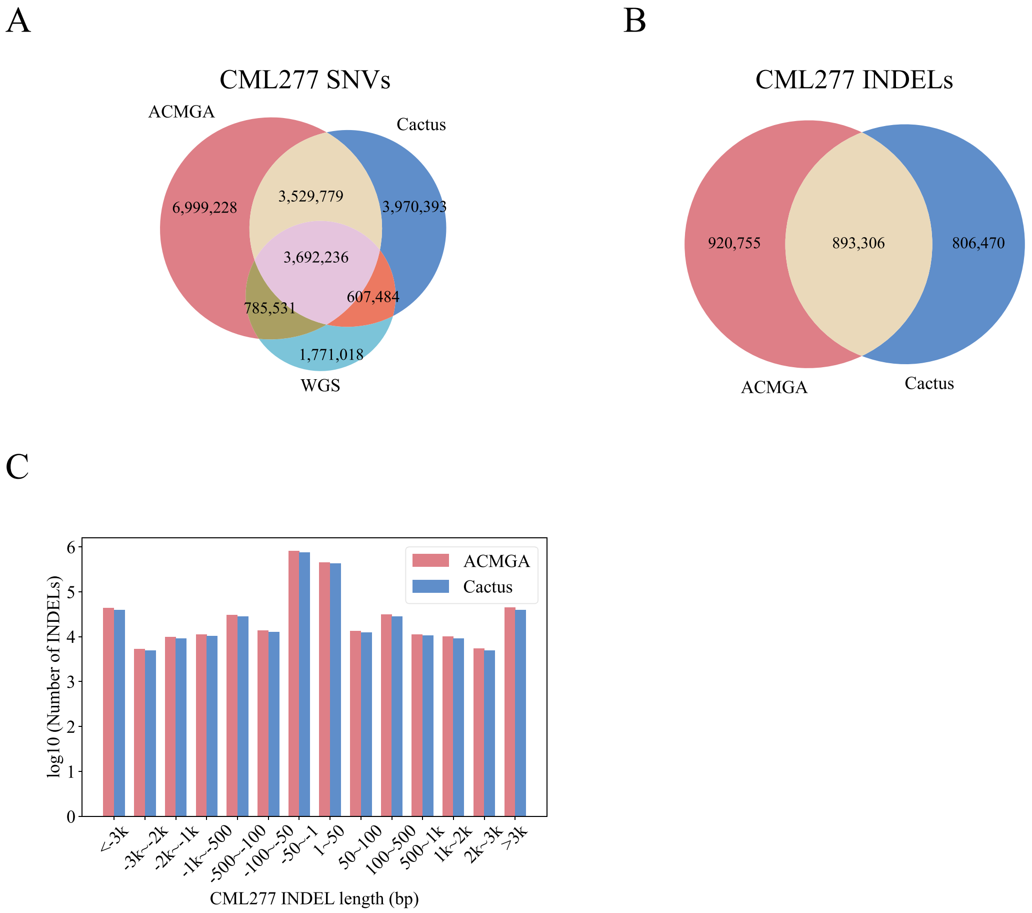

Fig. S15 Variant calling of different methods for maize (CML277). (A) The SNVs identified between B73 and CML277 from the MGA of 26 maize accessions using ACMGA and Cactus, and comparing them with WGS SNVs called by the Panzea project. **(B)** The INDELs (left alignment standardization) obtained by ACMGA, Cactus, and WGS. **(C)** The length distribution of INDELs obtained by ACMGA, Cactus, WGS.

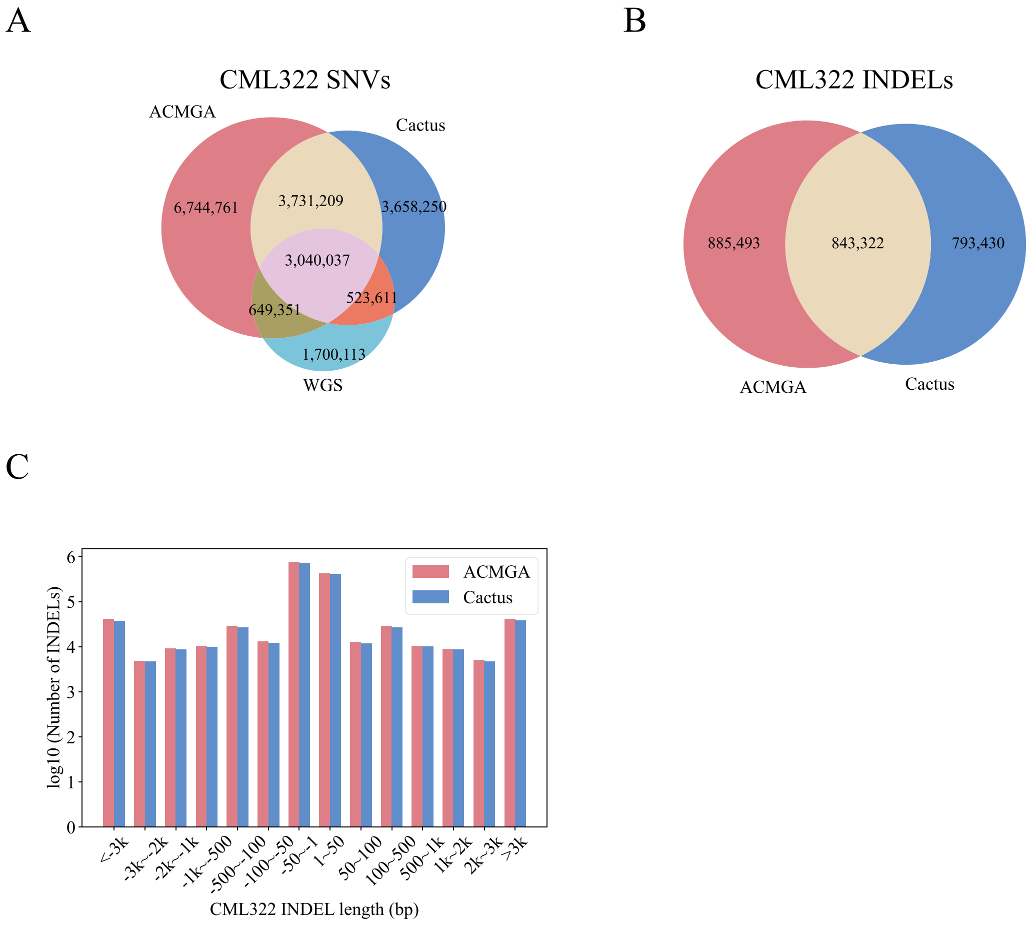

Fig. S16 Variant calling of different methods for maize (CML322). (A) The SNVs identified between B73 and CML322 from the MGA of 26 maize accessions using ACMGA and Cactus, and comparing them with WGS SNVs called by the Panzea project. **(B)** The INDELs (left alignment standardization) obtained by ACMGA, Cactus, and WGS. **(C)** The length distribution of INDELs obtained by ACMGA, Cactus, WGS.

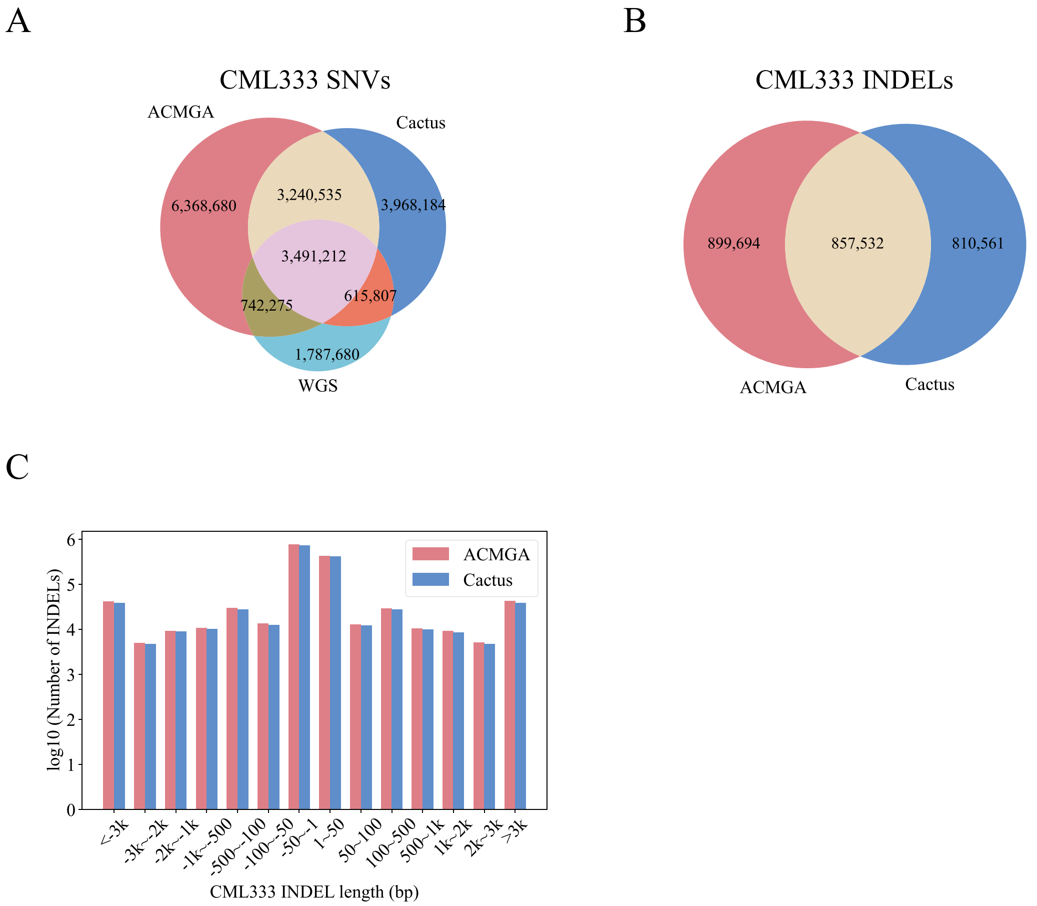

Fig. S17 Variant calling of different methods for maize (CML333). (A) The SNVs identified between B73 and CML333 from the MGA of 26 maize accessions using ACMGA and Cactus, and comparing them with WGS SNVs called by the Panzea project. **(B)** The INDELs (left alignment standardization) obtained by ACMGA, Cactus, and WGS. **(C)** The length distribution of INDELs obtained by ACMGA, Cactus, WGS.

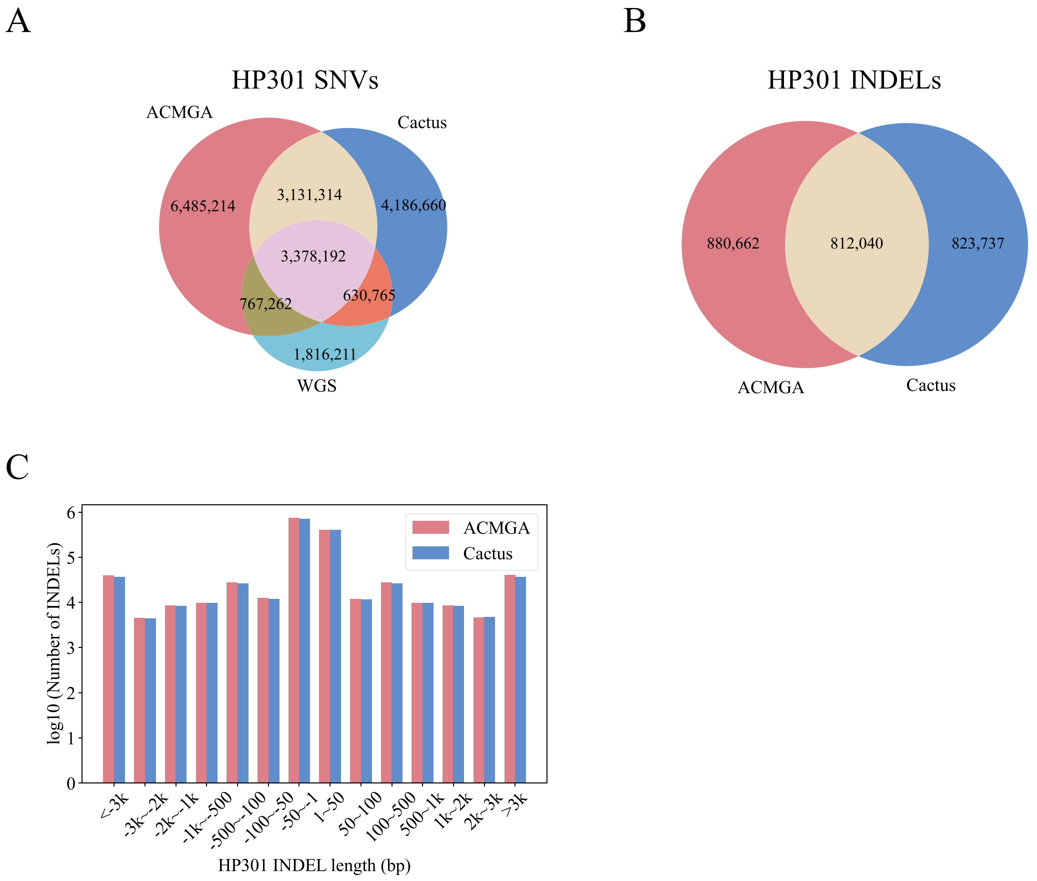

Fig. S18 Variant calling of different methods for maize (HP301). (A) The SNVs identified between B73 and HP301 from the MGA of 26 maize accessions using ACMGA and Cactus, and comparing them with WGS SNVs called by the Panzea project. **(B)** The INDELs (left alignment standardization) obtained by ACMGA, Cactus, and WGS. **(C)** The length distribution of INDELs obtained by ACMGA, Cactus, WGS.

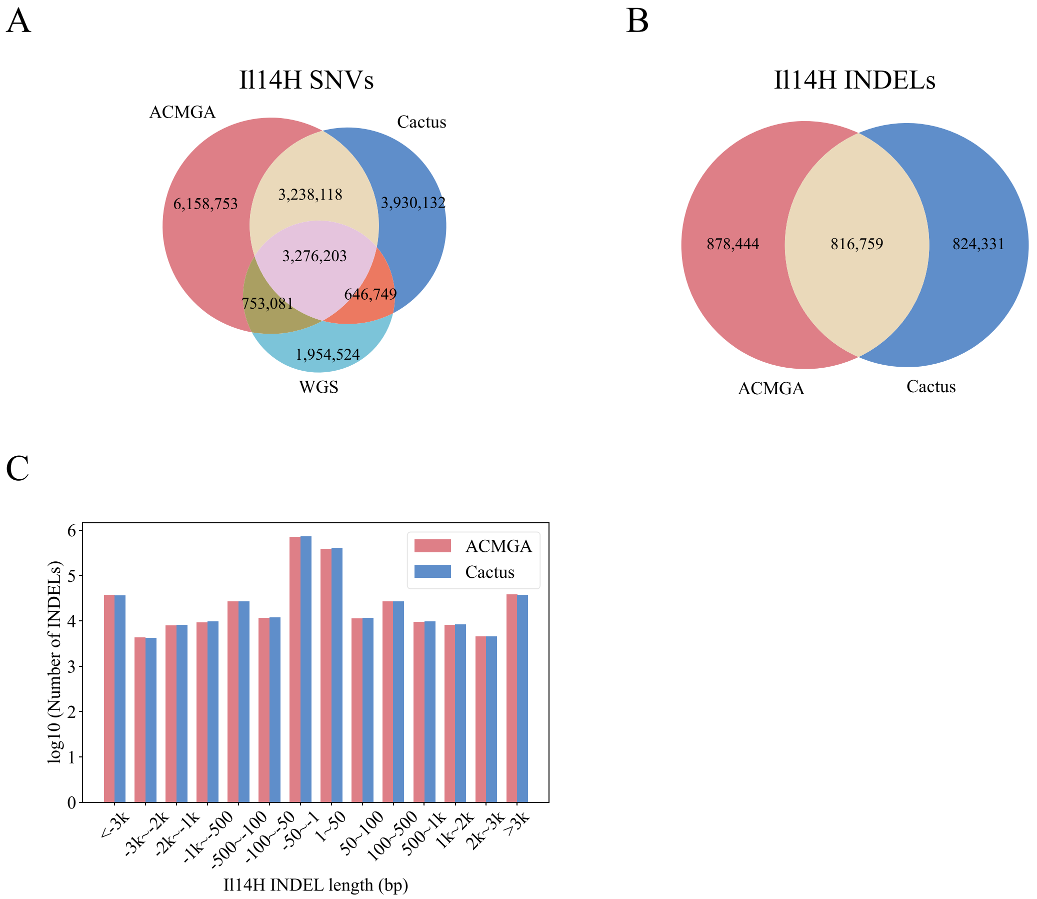

Fig. S19 Variant calling of different methods for maize (Il14H). (A) The SNVs identified between B73 and Il14H from the MGA of 26 maize accessions using ACMGA and Cactus, and comparing them with WGS SNVs called by the Panzea project. **(B)** The INDELs (left alignment standardization) obtained by ACMGA, Cactus, and WGS. **(C)** The length distribution of INDELs obtained by ACMGA, Cactus, WGS.

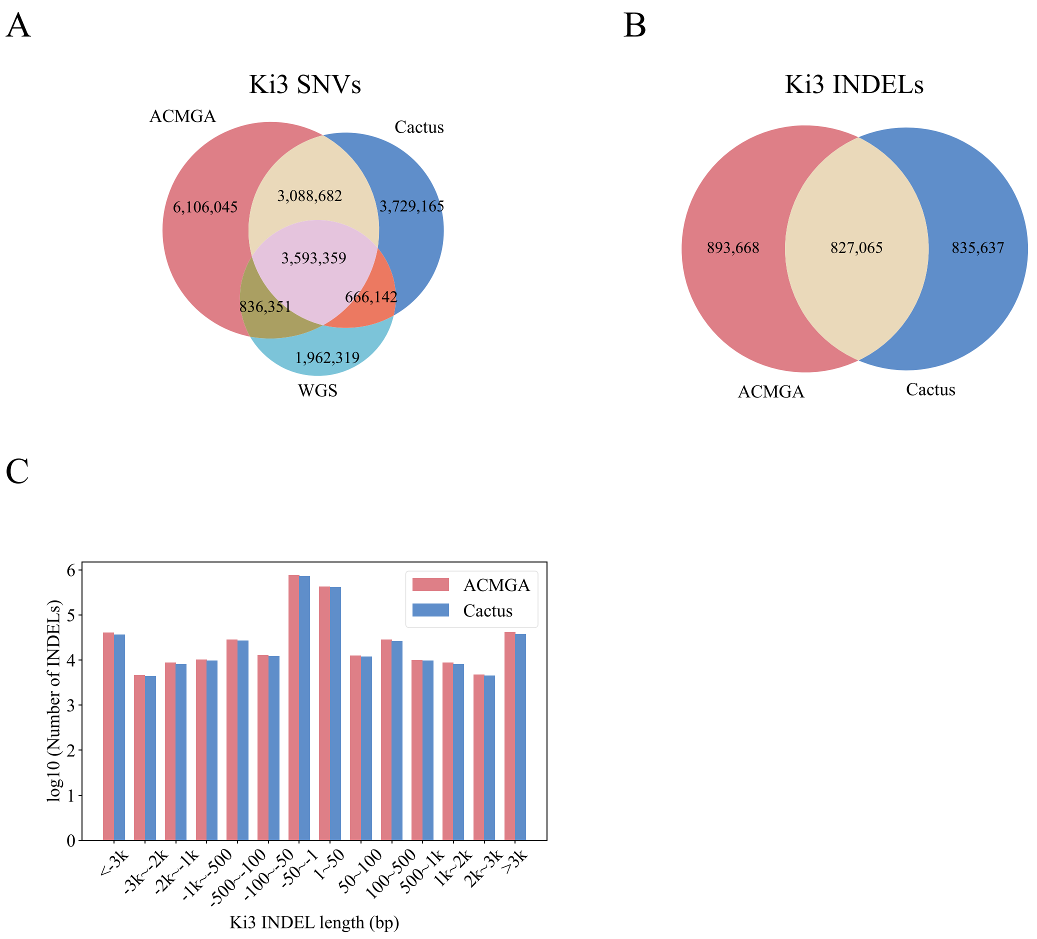

Fig. S20 Variant calling of different methods for maize (Ki3). (A) The SNVs identified between B73 and Ki3 from the MGA of 26 maize accessions using ACMGA and Cactus, and comparing them with WGS SNVs called by the Panzea project. **(B)** The INDELs (left alignment standardization) obtained by ACMGA, Cactus, and WGS. **(C)** The length distribution of INDELs obtained by ACMGA, Cactus, WGS.

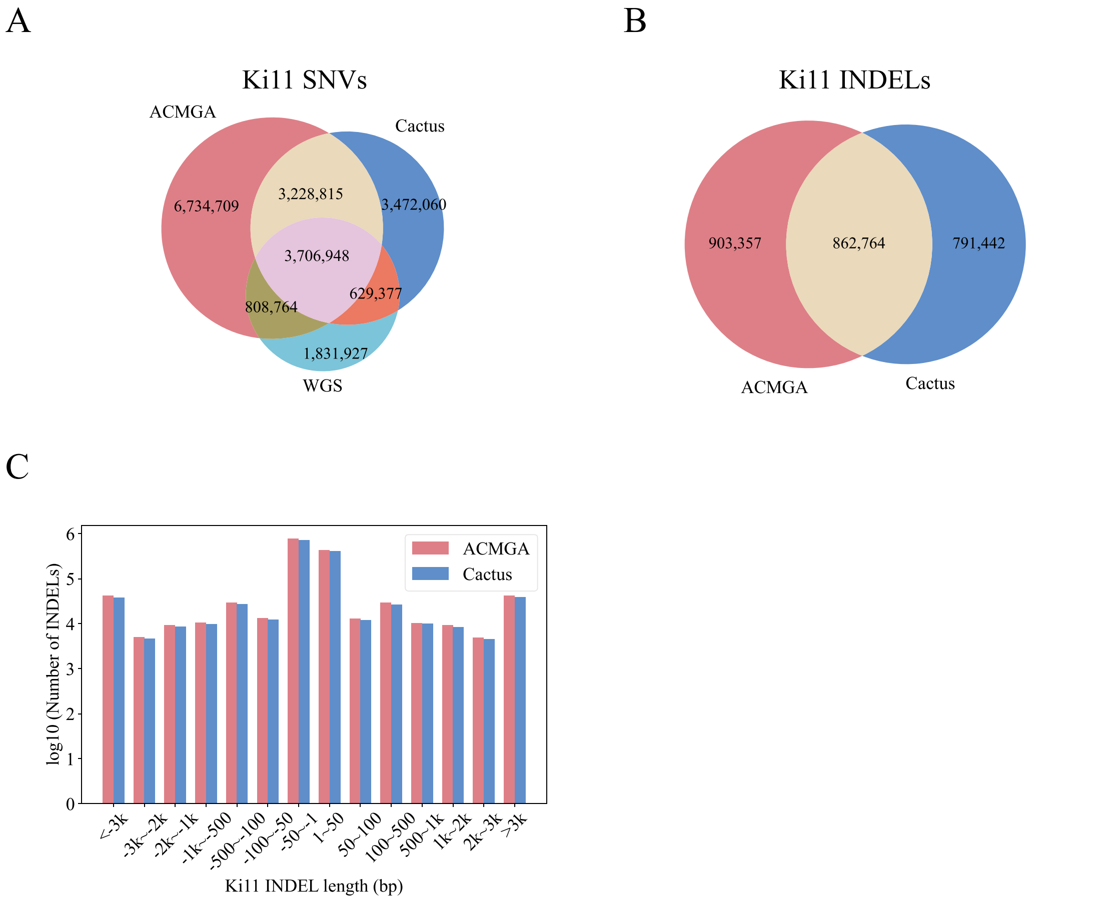

Fig. S21 Variant calling of different methods for maize (Kill). (A) The SNVs identified between B73 and Kill from the MGA of 26 maize accessions using ACMGA and Cactus, and comparing them with WGS SNVs called by the Panzea project. **(B)** The INDELs (left alignment standardization) obtained by ACMGA, Cactus, and WGS. **(C)** The length distribution of INDELs obtained by ACMGA, Cactus, WGS.

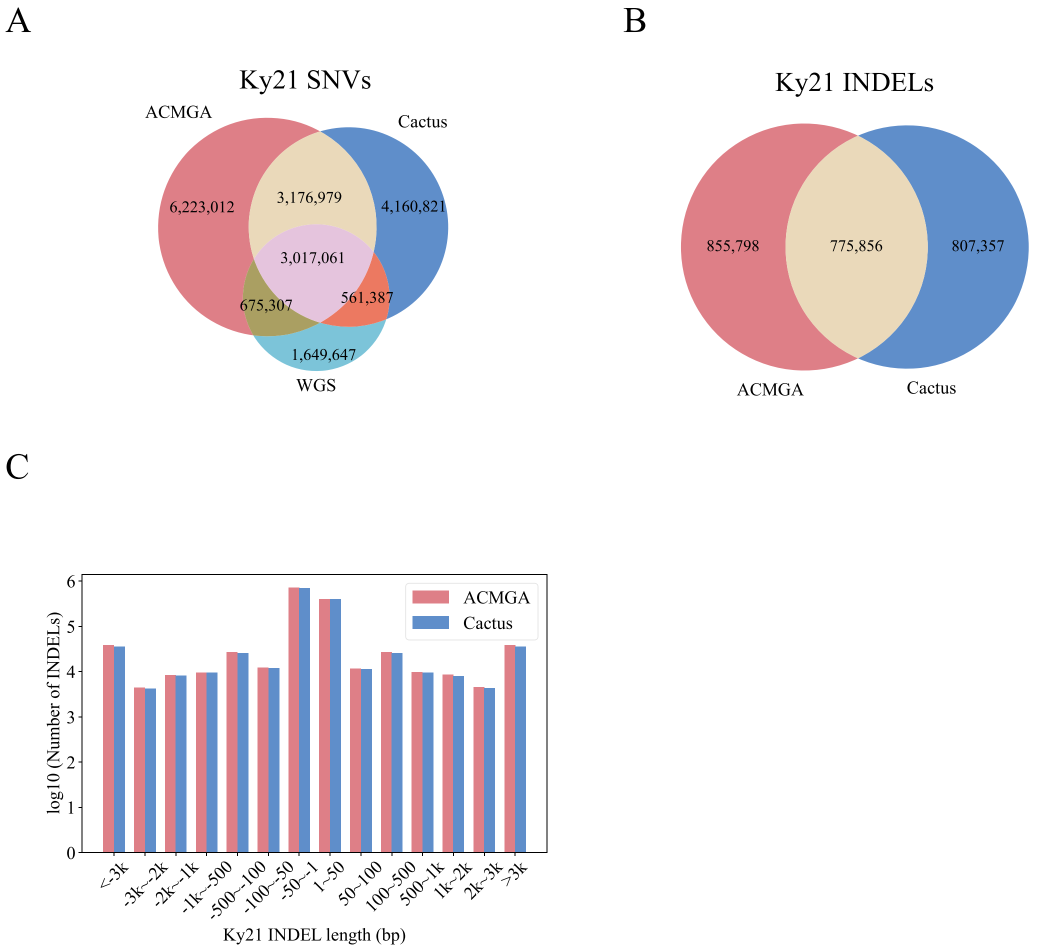

Fig. S22 Variant calling of different methods for maize (Ky21). (A) The SNVs identified between B73 and Ky21 from the MGA of 26 maize accessions using ACMGA and Cactus, and comparing them with WGS SNVs called by the Panzea project. **(B)** The INDELs (left alignment standardization) obtained by ACMGA, Cactus, and WGS. **(C)** The length distribution of INDELs obtained by ACMGA, Cactus, WGS.

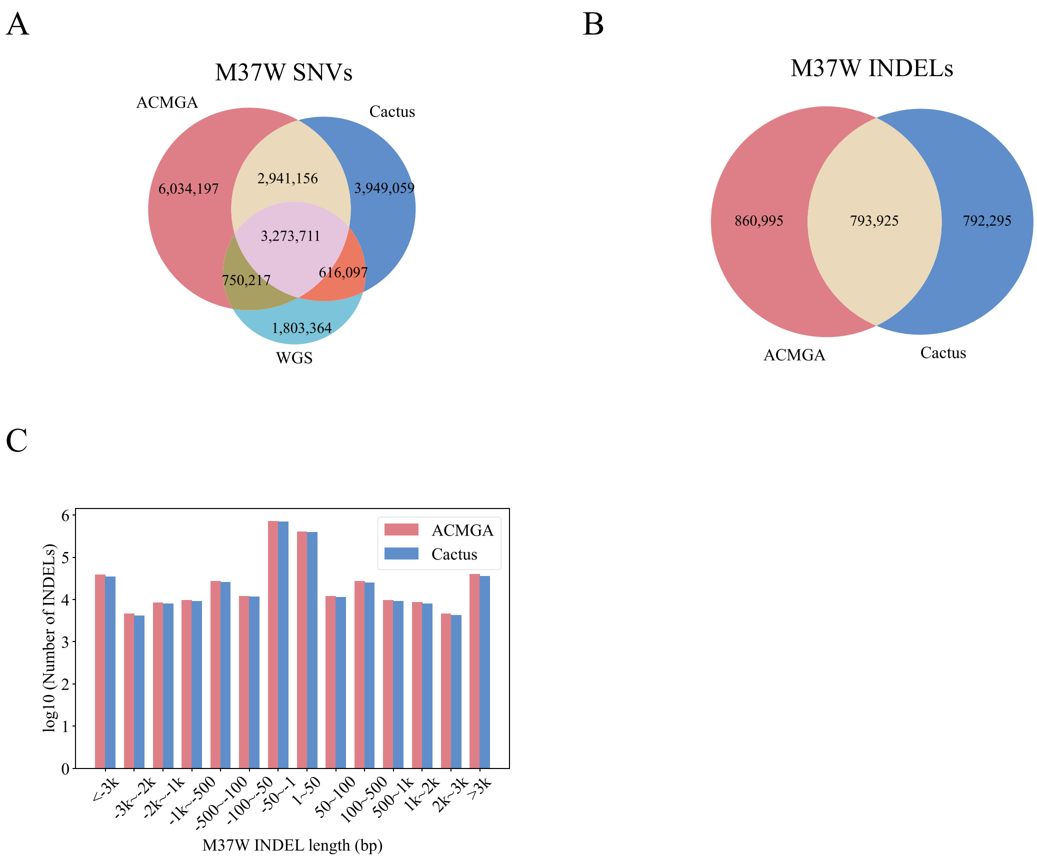

Fig. S23 Variant calling of different methods for maize (M37W). (A) The SNVs identified between B73 and M37W from the MGA of 26 maize accessions using ACMGA and Cactus, and comparing them with WGS SNVs called by the Panzea project. **(B)** The INDELs (left alignment standardization) obtained by ACMGA, Cactus, and WGS. **(C)** The length distribution of INDELs obtained by ACMGA, Cactus, WGS.

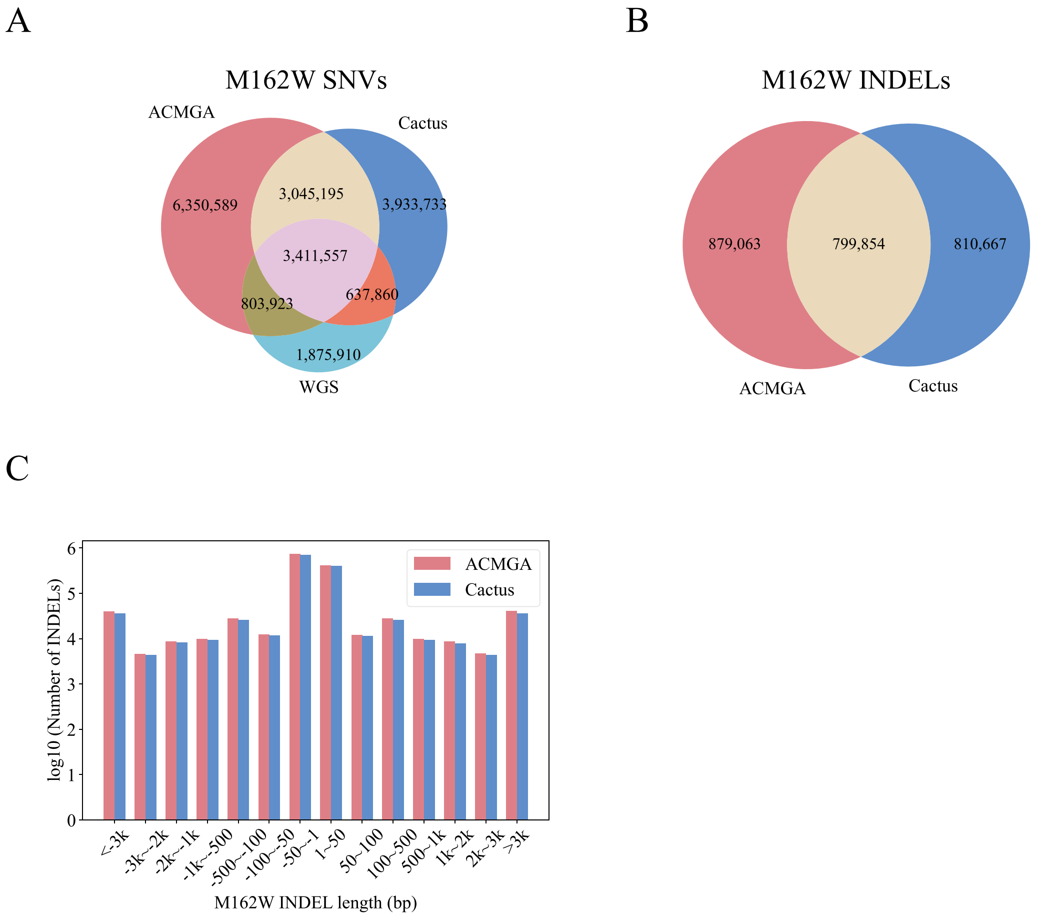

Fig. S24 Variant calling of different methods for maize (M162W). (A) The SNVs identified between B73 and M162W from the MGA of 26 maize accessions using ACMGA and Cactus, and comparing them with WGS SNVs called by the Panzea project. **(B)** The INDELs (left alignment standardization) obtained by ACMGA, Cactus, and WGS. **(C)** The length distribution of INDELs obtained by ACMGA, Cactus, WGS.

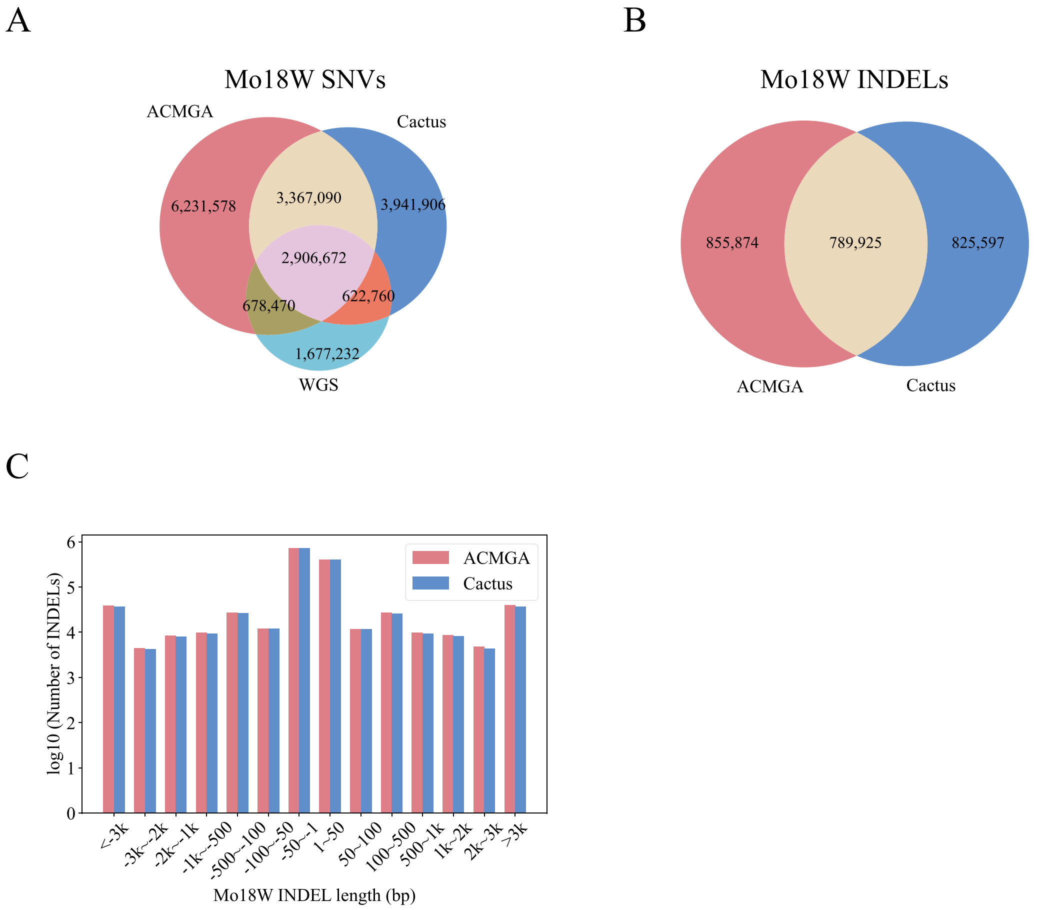

Fig. S25 Variant calling of different methods for maize (Mo18w). (A) The SNVs identified between B73 and Mo18w from the MGA of 26 maize accessions using ACMGA and Cactus, and comparing them with WGS SNVs called by the Panzea project. **(B)** The INDELs (left alignment standardization) obtained by ACMGA, Cactus, and WGS. **(C)** The length distribution of INDELs obtained by ACMGA, Cactus, WGS.

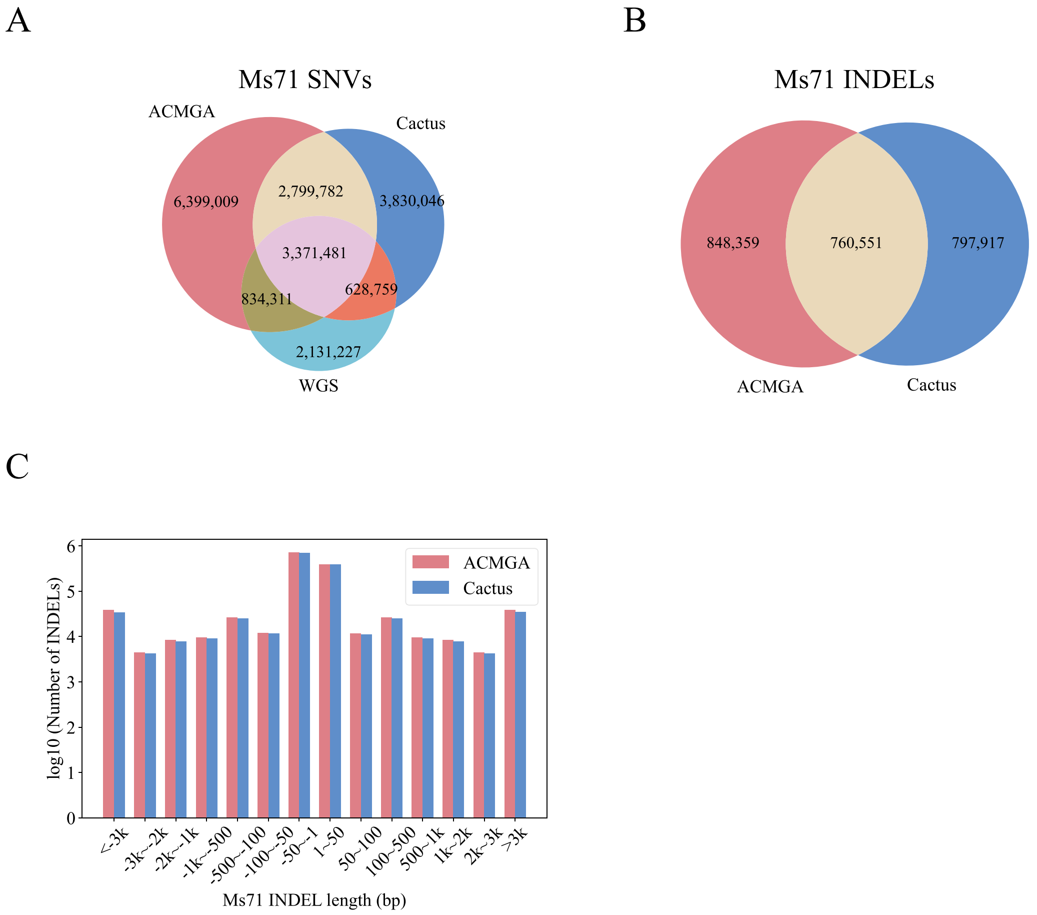

Fig. S26 Variant calling of different methods for maize (Ms71). (A) The SNVs identified between B73 and Ms71 from the MGA of 26 maize accessions using ACMGA and Cactus, and comparing them with WGS SNVs called by the Panzea project. **(B)** The INDELs (left alignment standardization) obtained by ACMGA, Cactus, and WGS. **(C)** The length distribution of INDELs obtained by ACMGA, Cactus, WGS.

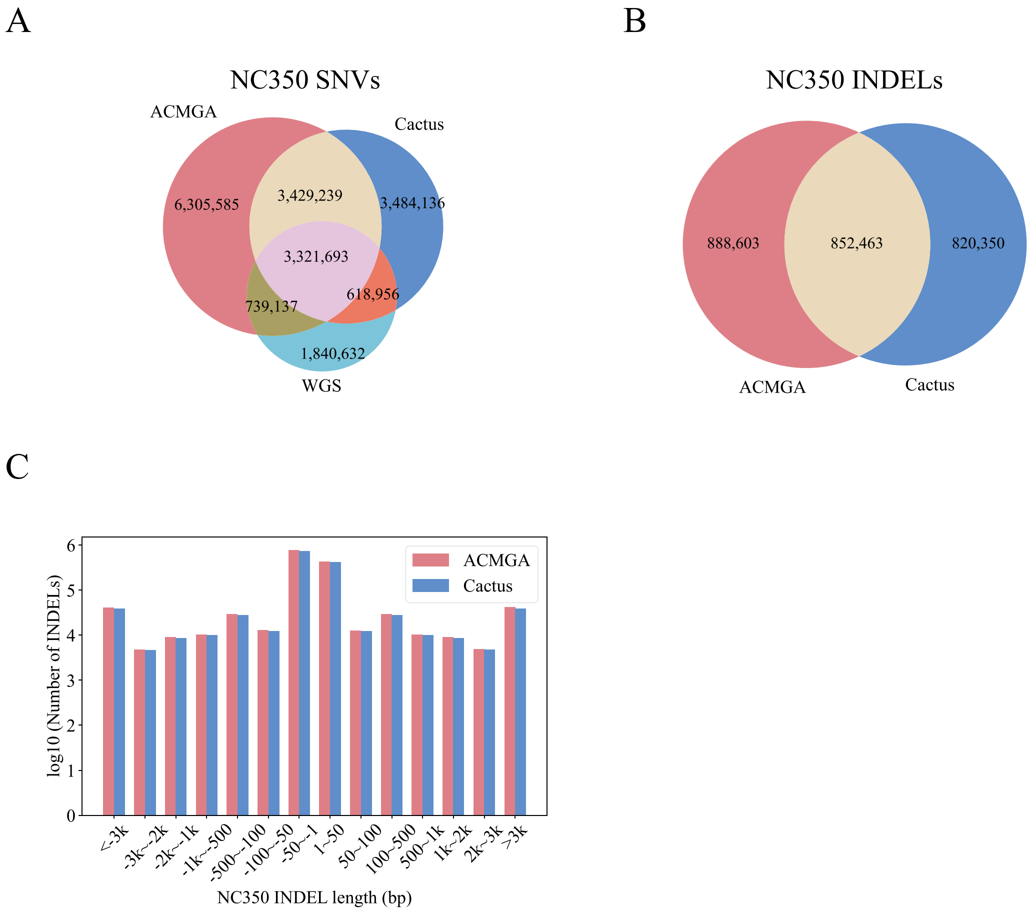

Fig. S27 Variant calling of different methods for maize (NC350). (A) The SNVs identified between B73 and NC350 from the MGA of 26 maize accessions using ACMGA and Cactus, and comparing them with WGS SNVs called by the Panzea project. **(B)** The INDELs (left alignment standardization) obtained by ACMGA, Cactus, and WGS. **(C)** The length distribution of INDELs obtained by ACMGA, Cactus, WGS.

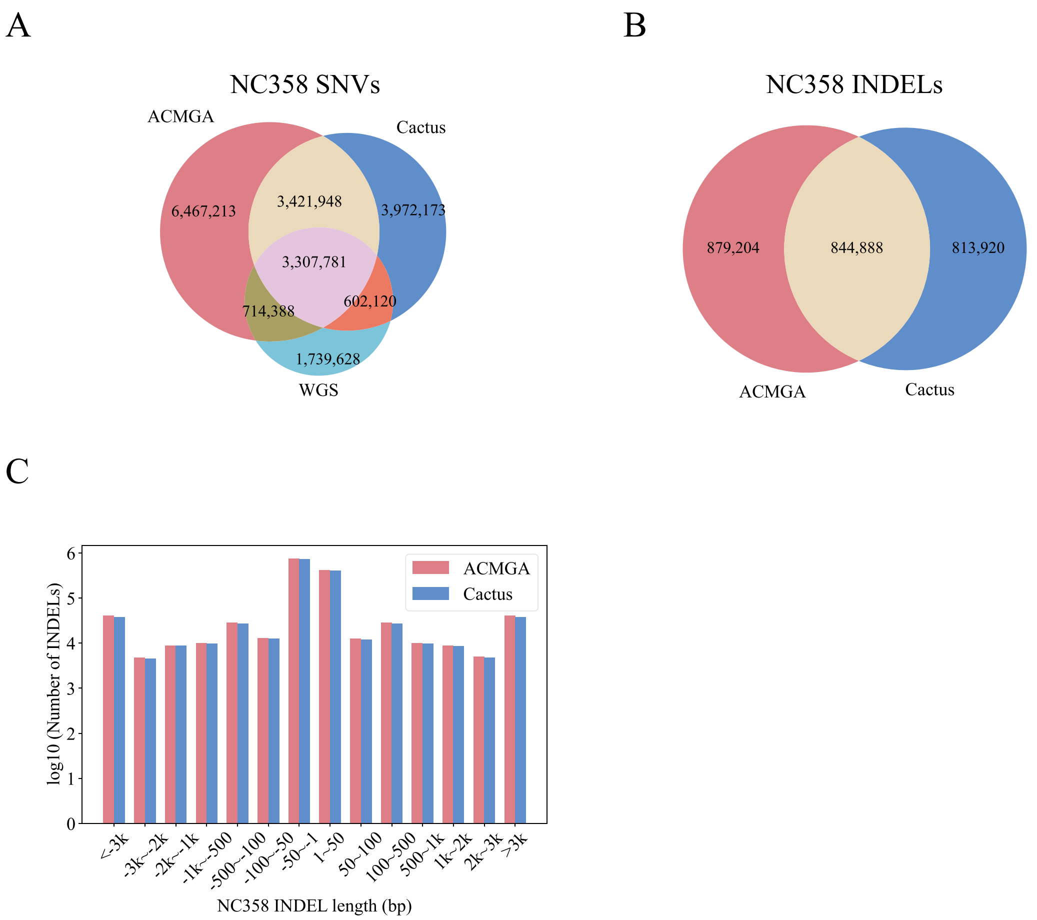

Fig. S28 Variant calling of different methods for maize (NC358). (A) The SNVs identified between B73 and NC358 from the MGA of 26 maize accessions using ACMGA and Cactus, and comparing them with WGS SNVs called by the Panzea project. **(B)** The INDELs (left alignment standardization) obtained by ACMGA, Cactus, and WGS. **(C)** The length distribution of INDELs obtained by ACMGA, Cactus, WGS.

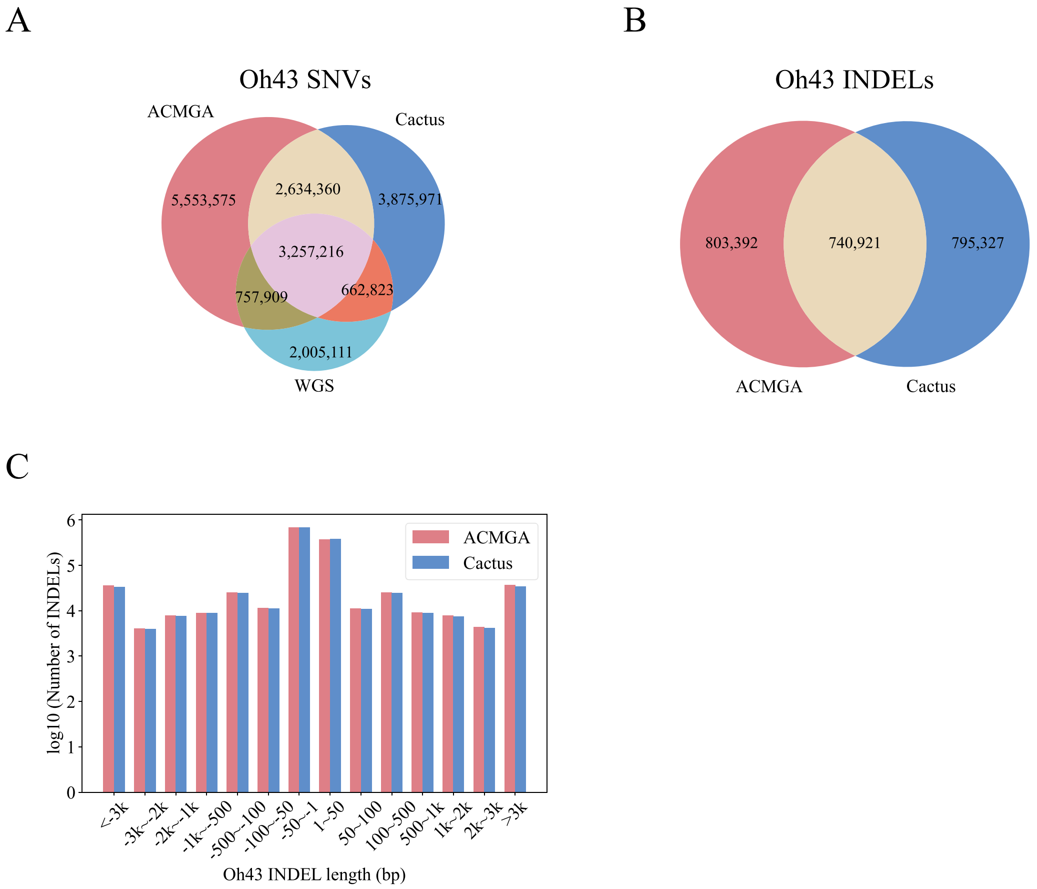

Fig. S29 Variant calling of different methods for maize (Oh43). (A) The SNVs identified between B73 and Oh43 from the MGA of 26 maize accessions using ACMGA and Cactus, and comparing them with WGS SNVs called by the Panzea project. **(B)** The INDELs (left alignment standardization) obtained by ACMGA, Cactus, and WGS. **(C)** The length distribution of INDELs obtained by ACMGA, Cactus, WGS.

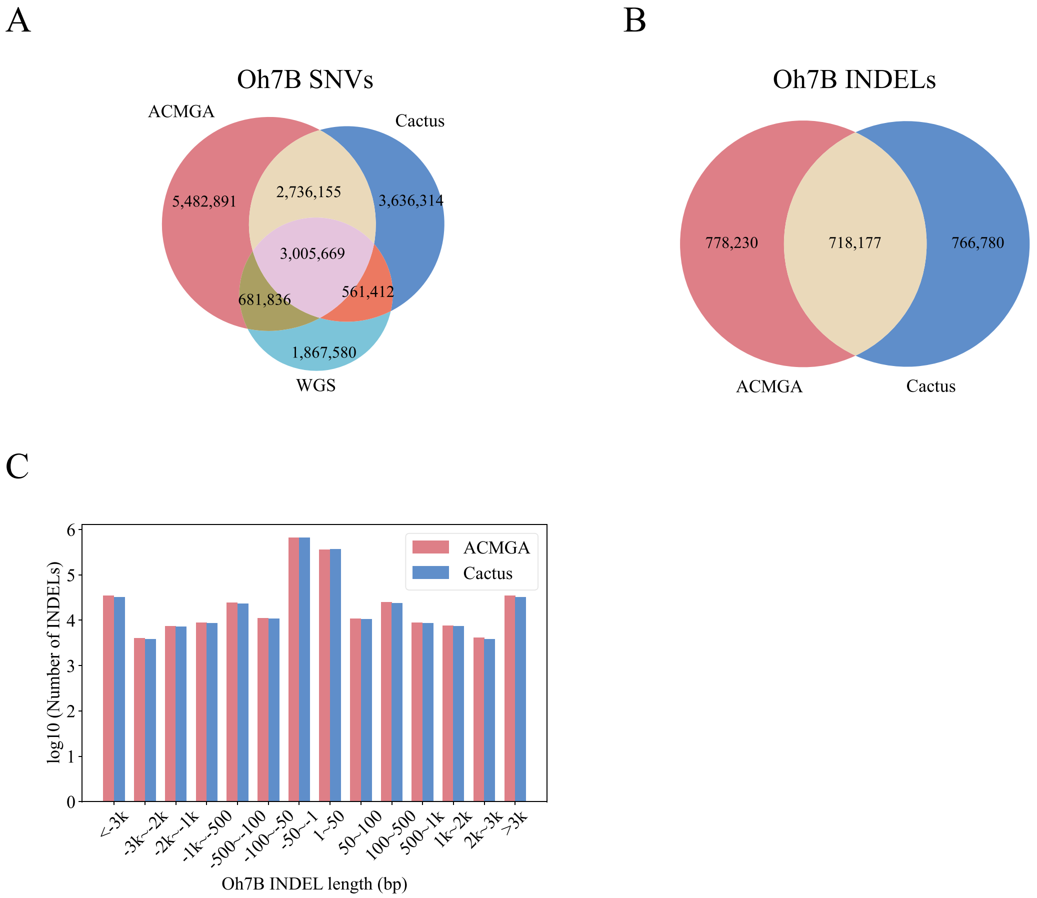

Fig. S30 Variant calling of different methods for maize (Oh7B). (A) The SNVs identified between B73 and Oh7B from the MGA of 26 maize accessions using ACMGA and Cactus, and comparing them with WGS SNVs called by the Panzea project. **(B)** The INDELs (left alignment standardization) obtained by ACMGA, Cactus, and WGS. **(C)** The length distribution of INDELs obtained by ACMGA, Cactus, WGS.

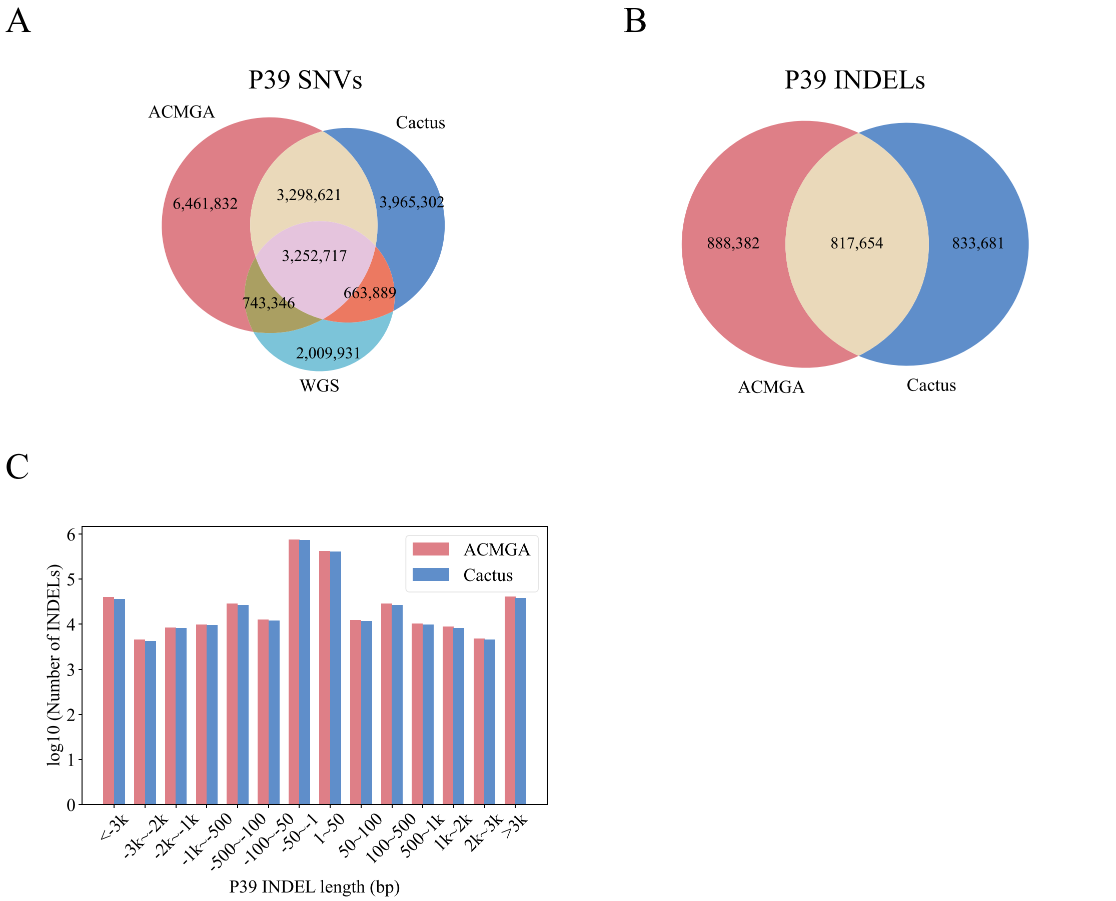

Fig. S31 Variant calling of different methods for maize (P39). (A) The SNVs identified between B73 and P39 from the MGA of 26 maize accessions using ACMGA and Cactus, and comparing them with WGS SNVs called by the Panzea project. **(B)** The INDELs (left alignment standardization) obtained by ACMGA, Cactus, and WGS. **(C)** The length distribution of INDELs obtained by ACMGA, Cactus, WGS.

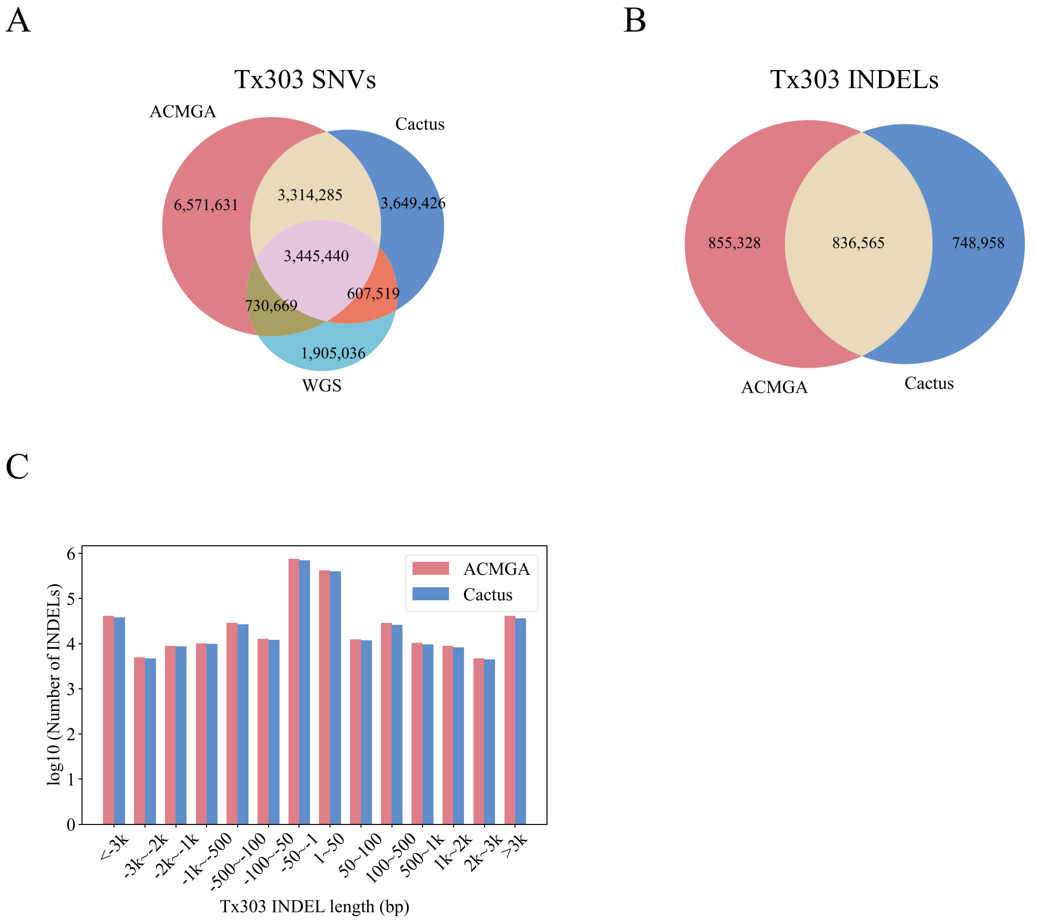

Fig. S32 Variant calling of different methods for maize (Tx303). (A) The SNVs identified between B73 and Tx303 from the MGA of 26 maize accessions using ACMGA and Cactus, and comparing them with WGS SNVs called by the Panzea project. **(B)** The INDELs (left alignment standardization) obtained by ACMGA, Cactus, and WGS. **(C)** The length distribution of INDELs obtained by ACMGA, Cactus, WGS.

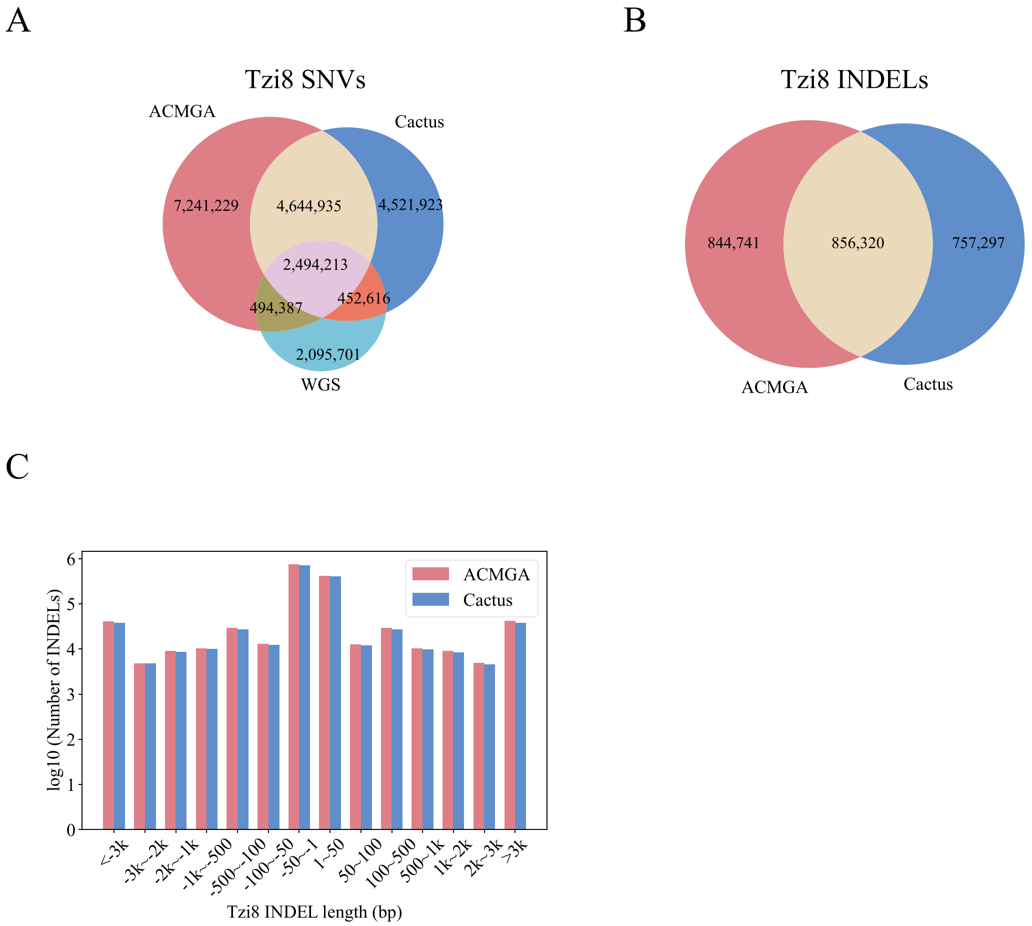

Fig. S33 Variant calling of different methods for maize (Tzi8). (A) The SNVs identified between B73 and Tzi8 from the MGA of 26 maize accessions using ACMGA and Cactus, and comparing them with WGS SNVs called by the Panzea project. **(B)** The INDELs (left alignment standardization) obtained by ACMGA, Cactus, and WGS. **(C)** The length distribution of INDELs obtained by ACMGA, Cactus, WGS.

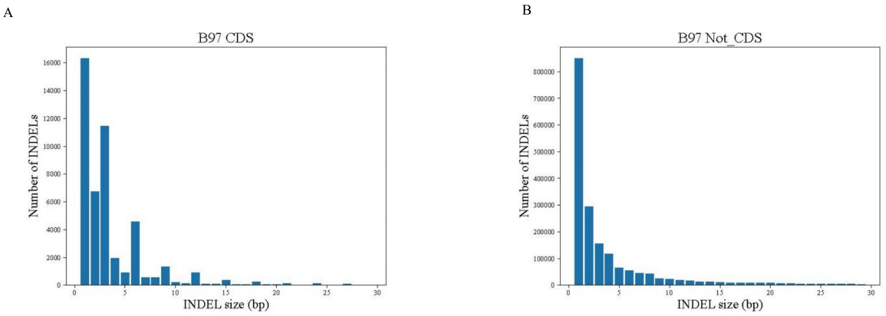

Fig. S34 Length distribution of INDELs identified by ACMGA and Cactus in CDS and Non-CDS regions (B97). Most frequent INDEL sizes differ between coding and non-coding regions. Multiple of three are much more common in coding sequences.

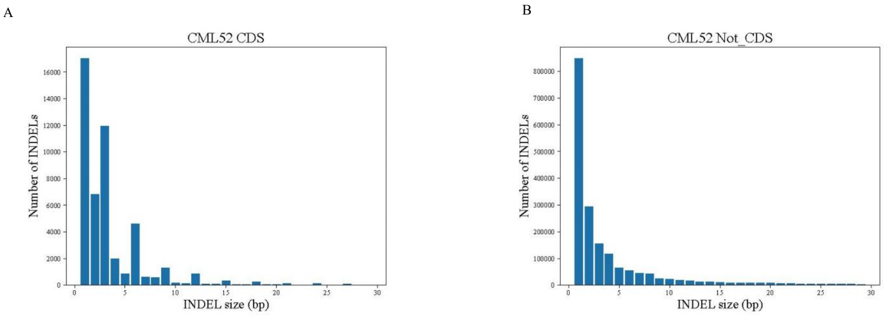

Fig. S35 Length distribution of INDELs identified by ACMGA and Cactus in CDS and Non-CDS regions (CML52). Most frequent INDEL sizes differ between coding and non-coding regions. Multiple of three are much more common in coding sequences.

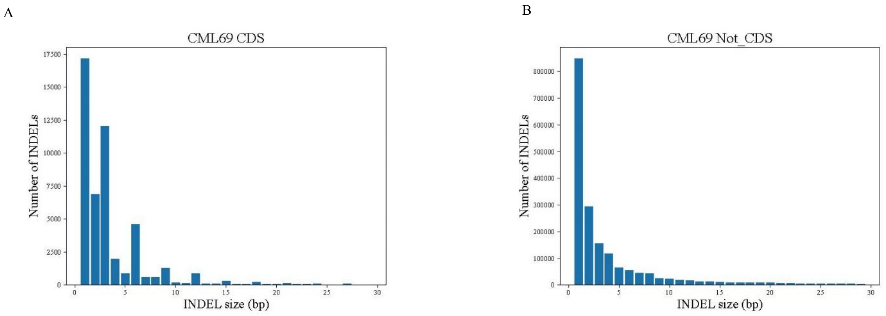

Fig. S36 Length distribution of INDELs identified by ACMGA and Cactus in CDS and Non-CDS regions (CML69). Most frequent INDEL sizes differ between coding and non-coding regions. Multiple of three are much more common in coding sequences.

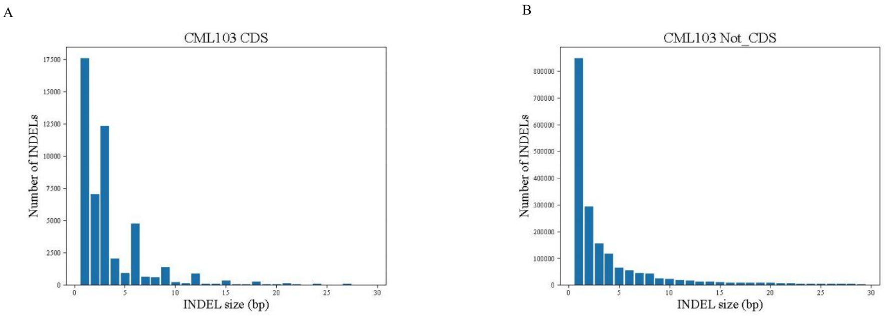

Fig. S37 Length distribution of INDELs identified by ACMGA and Cactus in CDS and Non-CDS regions (CML103). Most frequent INDEL sizes differ between coding and non-coding regions. Multiple of three are much more common in coding sequences.

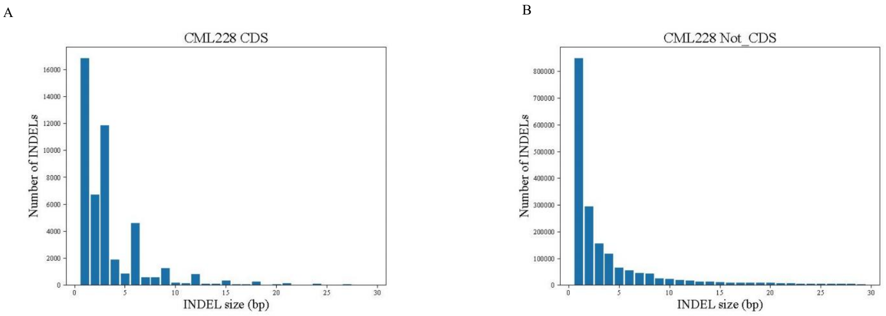

Fig. S38 Length distribution of INDELs identified by ACMGA and Cactus in CDS and Non-CDS regions (CML228). Most frequent INDEL sizes differ between coding and non-coding regions. Multiple of three are much more common in coding sequences.

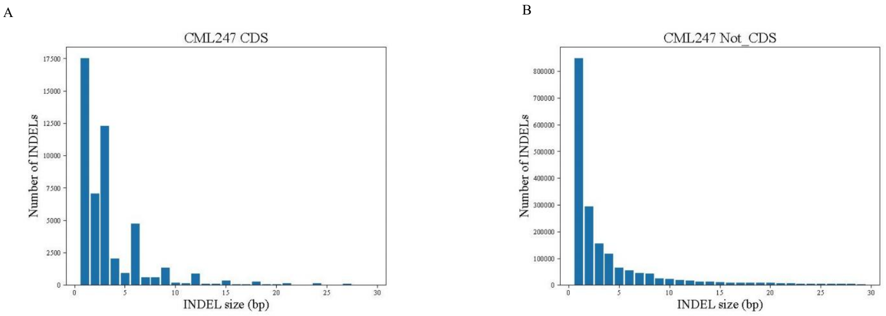

Fig. S39 Length distribution of INDELs identified by ACMGA and Cactus in CDS and Non-CDS regions (CML247). Most frequent INDEL sizes differ between coding and non-coding regions. Multiple of three are much more common in coding sequences.

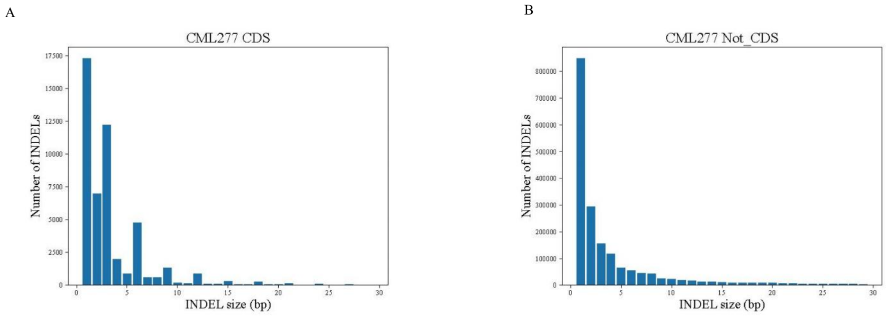

Fig. S40 Length distribution of INDELs identified by ACMGA and Cactus in CDS and Non-CDS regions (CML277). Most frequent INDEL sizes differ between coding and non-coding regions. Multiple of three are much more common in coding sequences.

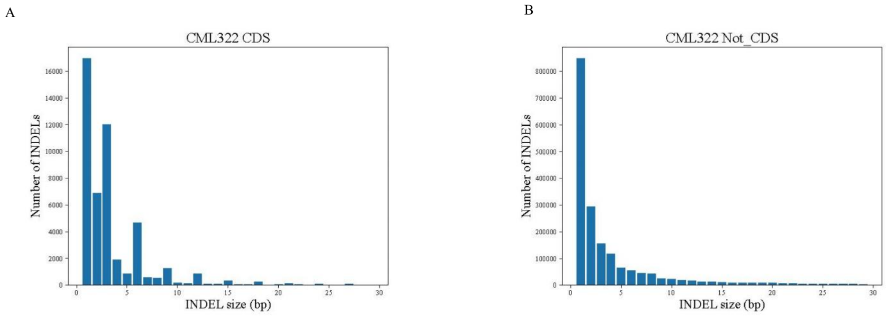

Fig. S41 Length distribution of INDELs identified by ACMGA and Cactus in CDS and Non-CDS regions (CML322). Most frequent INDEL sizes differ between coding and non-coding regions. Multiple of three are much more common in coding.

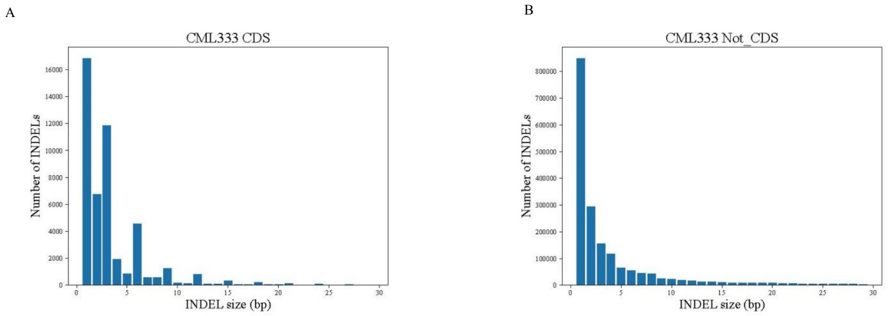

Fig. S42 Length distribution of INDELs identified by ACMGA and Cactus in CDS and Non-CDS regions (CML333). Most frequent INDEL sizes differ between coding and non-coding regions. Multiple of three are much more common in coding.

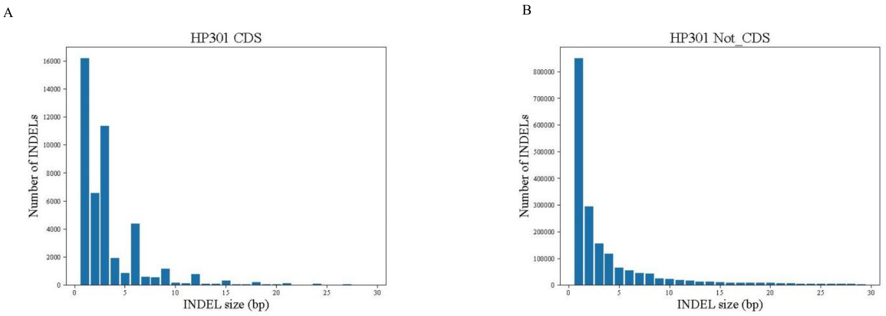

Fig. S43 Length distribution of INDELs identified by ACMGA and Cactus in CDS and Non-CDS regions (HP301). Most frequent INDEL sizes differ between coding and non-coding regions. Multiple of three are much more common in coding.

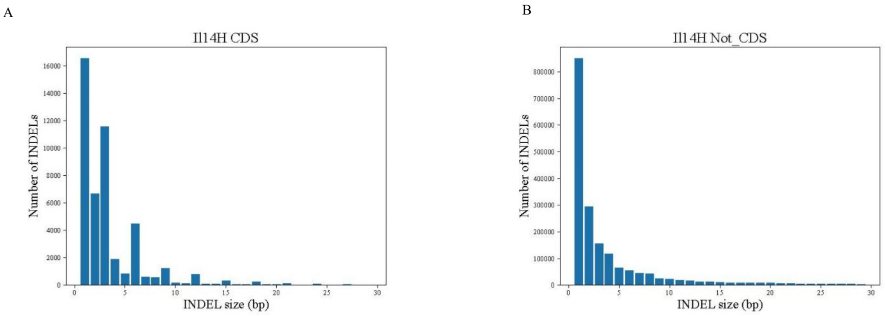

Fig. S44 Length distribution of INDELs identified by ACMGA and Cactus in CDS and Non-CDS regions (Il14H). Most frequent INDEL sizes differ between coding and non-coding regions. Multiple of three are much more common in coding.

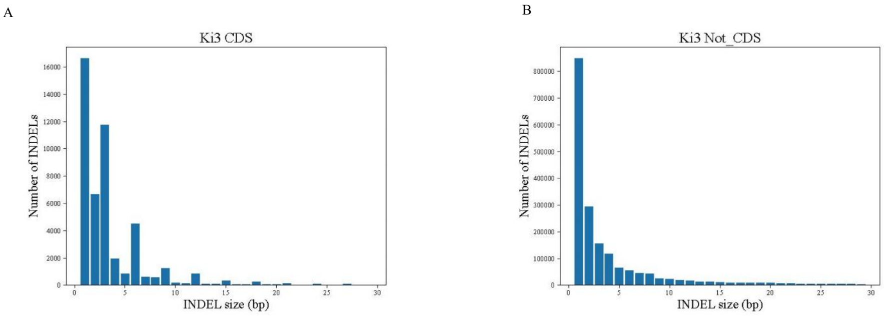

Fig. S45 Length distribution of INDELs identified by ACMGA and Cactus in CDS and Non-CDS regions (Ki3). Most frequent INDEL sizes differ between coding and non-coding regions. Multiple of three are much more common in coding

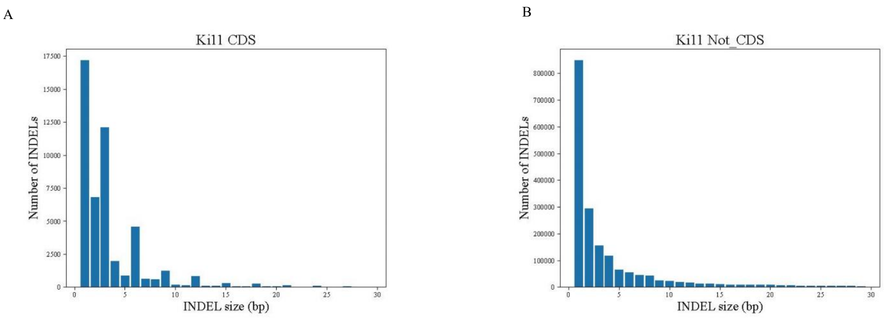

Fig. S46 Length distribution of INDELs identified by ACMGA and Cactus in CDS and Non-CDS regions (Kill). Most frequent INDEL sizes differ between coding and non-coding regions. Multiple of three are much more common in coding

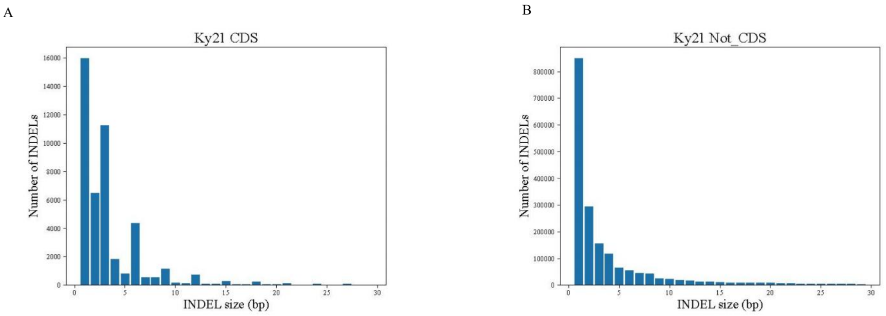

Fig. S47 Length distribution of INDELs identified by ACMGA and Cactus in CDS and Non-CDS regions (Ky21). Most frequent INDEL sizes differ between coding and non-coding regions. Multiple of three are much more common in coding

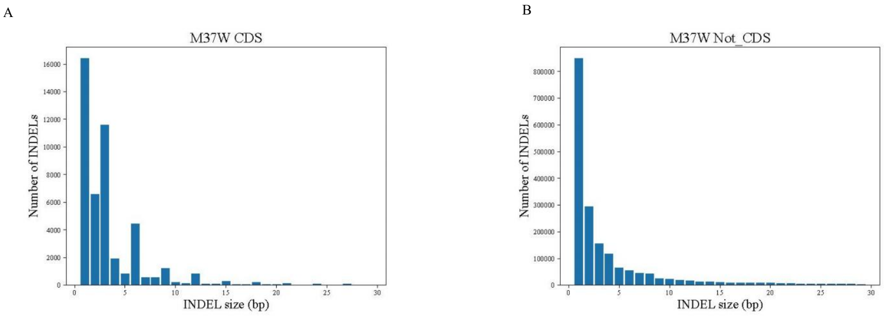

Fig. S48 Length distribution of INDELs identified by ACMGA and Cactus in CDS and Non-CDS regions (M37W). Most frequent INDEL sizes differ between coding and non-coding regions. Multiple of three are much more common in coding

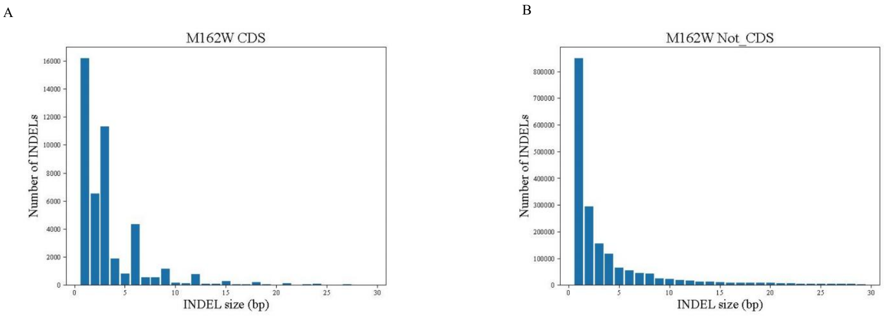

Fig. S49 Length distribution of INDELs identified by ACMGA and Cactus in CDS and Non-CDS regions (M162W). Most frequent INDEL sizes differ between coding and non-coding regions. Multiple of three are much more common in coding

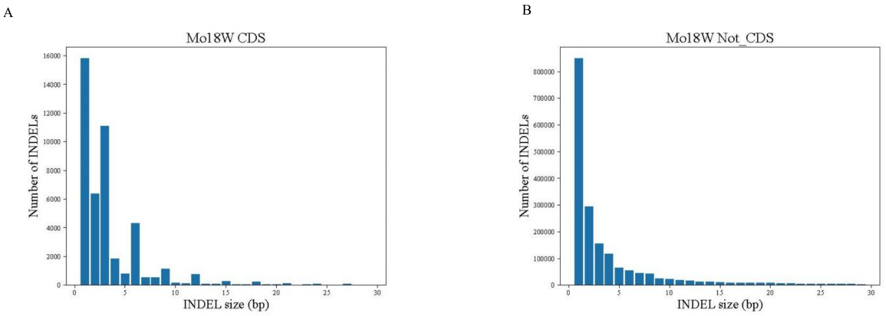

Fig. S50 Length distribution of INDELs identified by ACMGA and Cactus in CDS and Non-CDS regions (Mo18W). Most frequent INDEL sizes differ between coding and non-coding regions. Multiple of three are much more common in coding

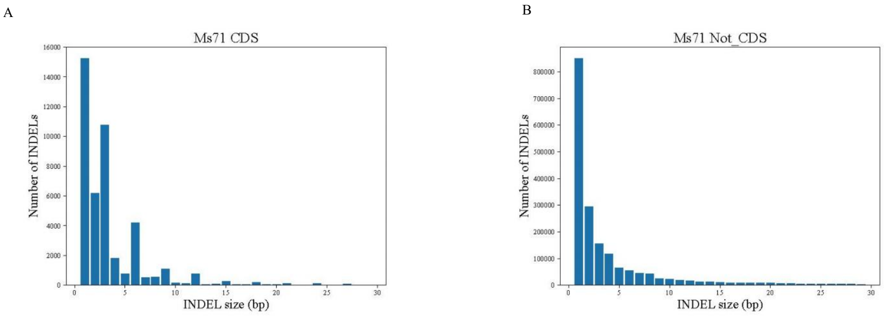

Fig. S51 Length distribution of INDELs identified by ACMGA and Cactus in CDS and Non-CDS regions (Ms71). Most frequent INDEL sizes differ between coding and non-coding regions. Multiple of three are much more common in coding

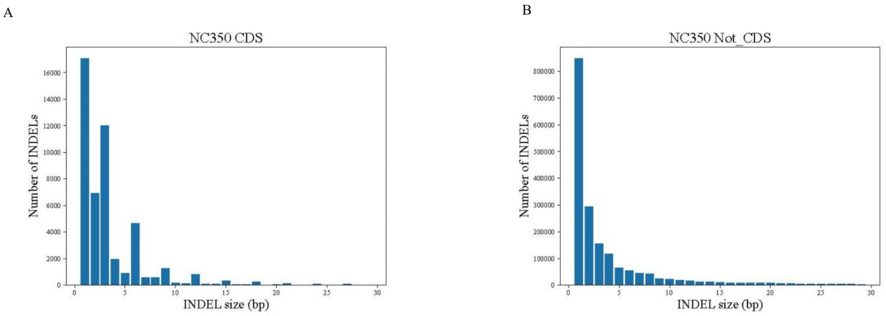

Fig. S52 Length distribution of INDELs identified by ACMGA and Cactus in CDS and Non-CDS regions (NC350). Most frequent INDEL sizes differ between coding and non-coding regions. Multiple of three are much more common in coding

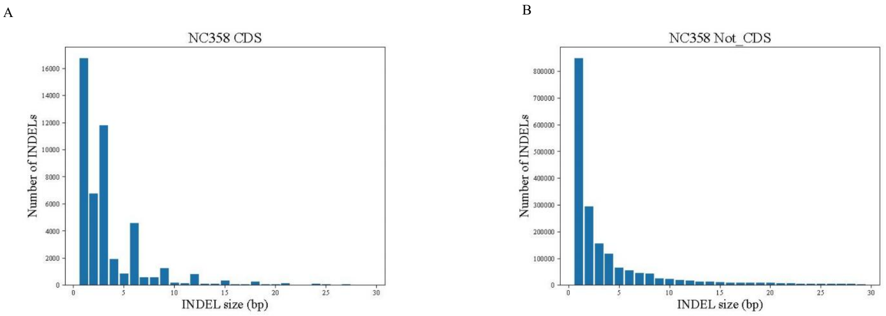

Fig. S53 Length distribution of INDELs identified by ACMGA and Cactus in CDS and Non-CDS regions (NC358). Most frequent INDEL sizes differ between coding and non-coding regions. Multiple of three are much more common in coding

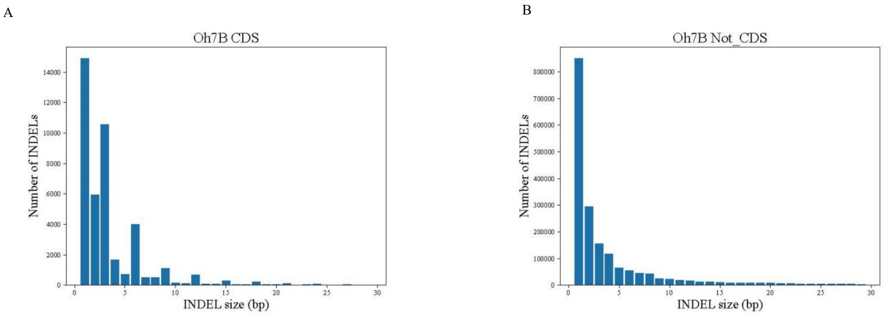

Fig. S54 Length distribution of INDELs identified by ACMGA and Cactus in CDS and Non-CDS regions (Oh7B). Most frequent INDEL sizes differ between coding and non-coding regions. Multiple of three are much more common in coding

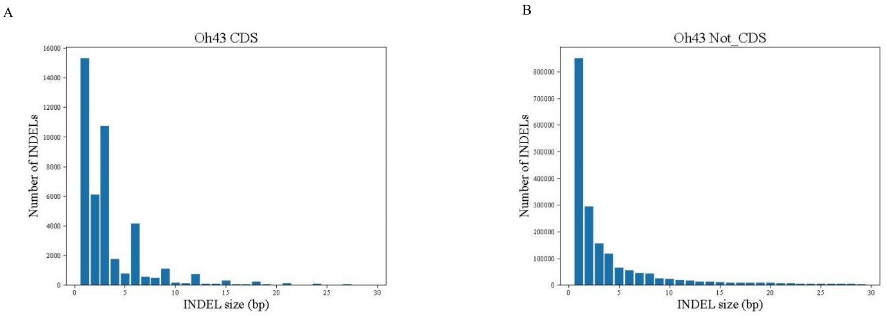

Fig. S55 Length distribution of INDELs identified by ACMGA and Cactus in CDS and Non-CDS regions (oh43). Most frequent INDEL sizes differ between coding and non-coding regions. Multiple of three are much more common in coding

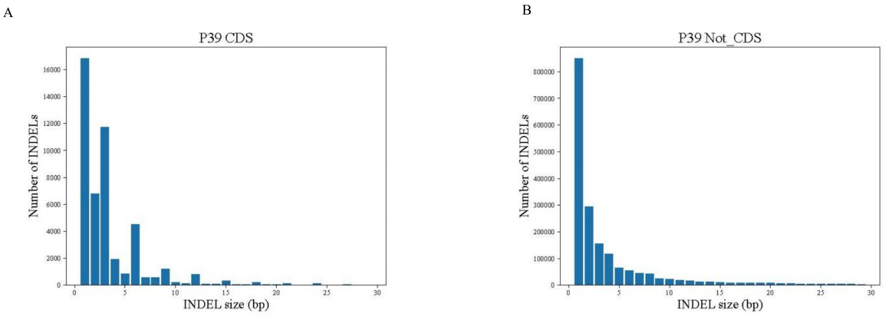

Fig. S56 Length distribution of INDELs identified by ACMGA and Cactus in CDS and Non-CDS regions (P39). Most frequent INDEL sizes differ between coding and non-coding regions. Multiple of three are much more common in coding

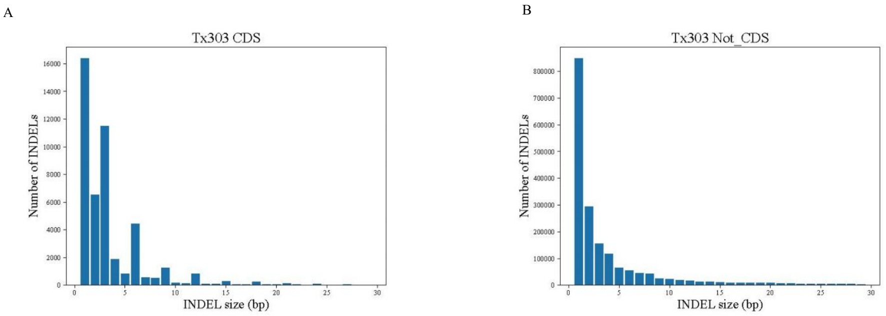

Fig. S57 Length distribution of INDELs identified by ACMGA and Cactus in CDS and Non-CDS regions (Tx303). Most frequent INDEL sizes differ between coding and non-coding regions. Multiple of three are much more common in coding

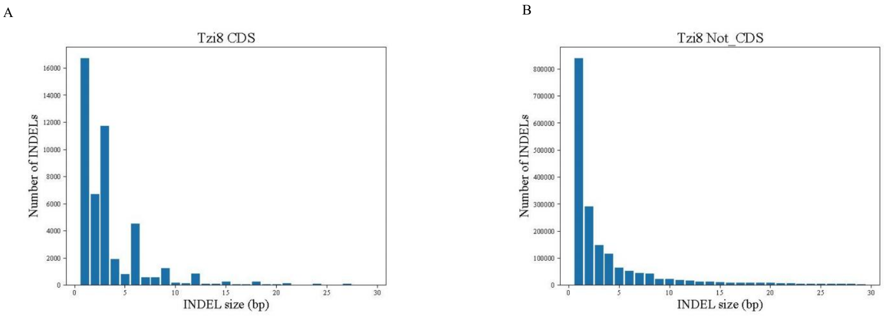

Fig. S58 Length distribution of INDELs identified by ACMGA and Cactus in CDS and Non-CDS regions (Tzi8). Most frequent INDEL sizes differ between coding and non-coding regions. Multiple of three are much more common in coding

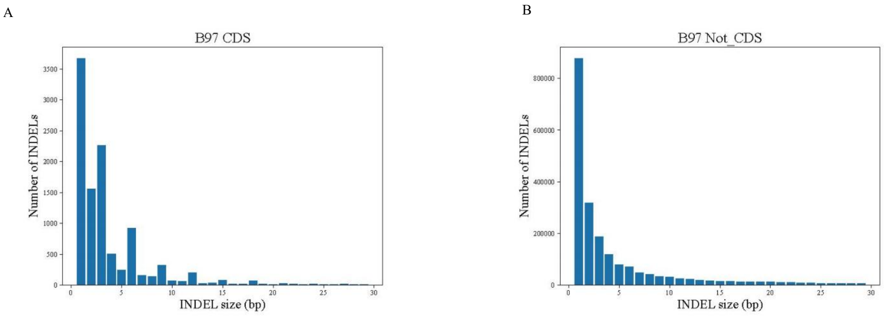

Fig. 59 Length distribution of INDELs identified solely by ACMGA in CDS and Non-CDS regions (B97). Most frequent INDEL sizes differ between coding and non-coding regions. Multiple of three are much more common in coding sequences.

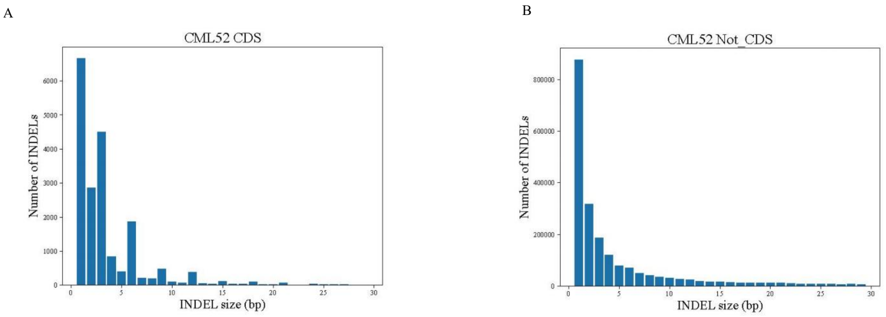

Fig. S60 Length distribution of INDELs identified solely by ACMGA in CDS and Non-CDS regions (CML52). Most frequent INDEL sizes differ between coding and non-coding regions. Multiple of three are much more common in coding sequences.

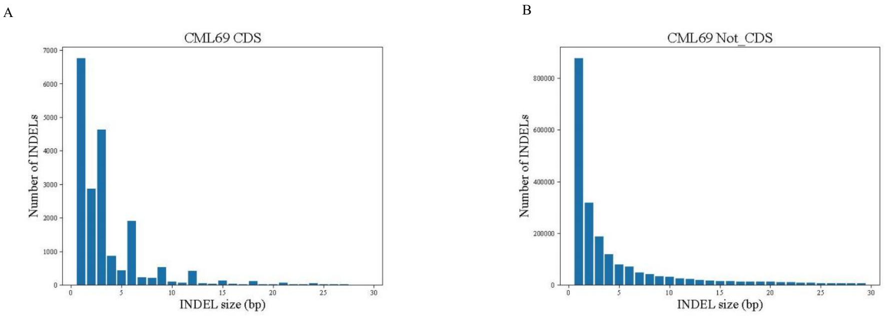

Fig. S61 Length distribution of INDELs identified solely by ACMGA in CDS and Non-CDS regions (CML69). Most frequent INDEL sizes differ between coding and non-coding regions. Multiple of three are much more common in coding sequences.

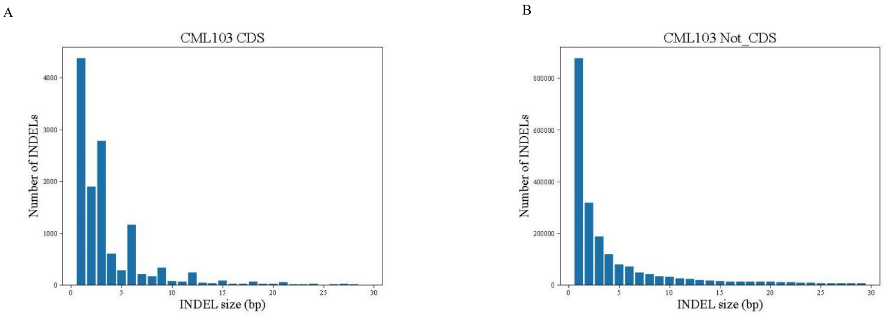

Fig. S62 Length distribution of INDELs identified solely by ACMGA in CDS and Non-CDS regions (CML103). Most frequent INDEL sizes differ between coding and non-coding regions. Multiple of three are much more common in coding sequences.

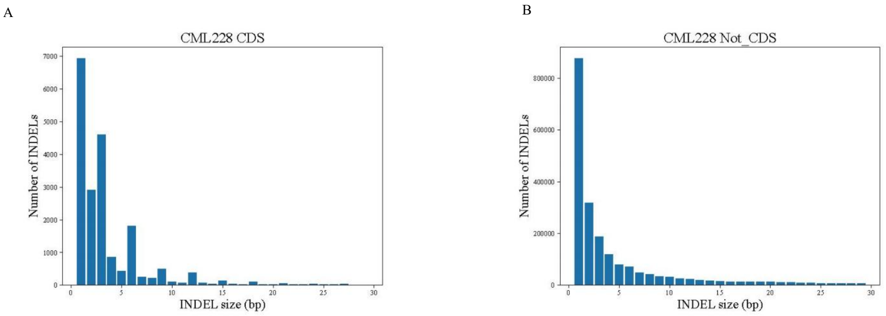

Fig. S63 Length distribution of INDELs identified solely by ACMGA in CDS and Non-CDS regions (CML228). Most frequent INDEL sizes differ between coding and non-coding regions. Multiple of three are much more common in coding sequences.

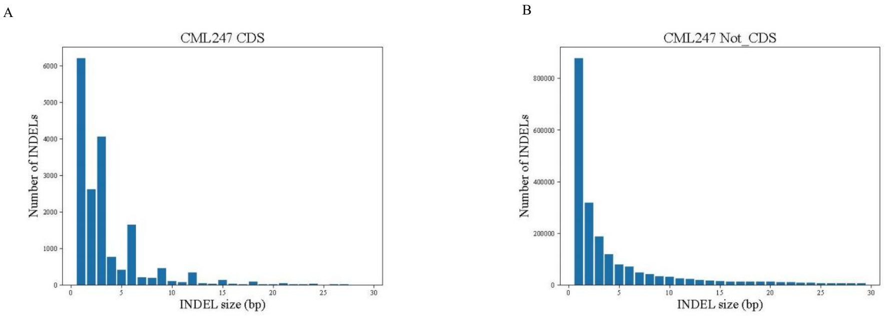

Fig. S64 Length distribution of INDELs identified solely by ACMGA in CDS and Non-CDS regions (CML247). Most frequent INDEL sizes differ between coding and non-coding regions. Multiple of three are much more common in coding sequences.

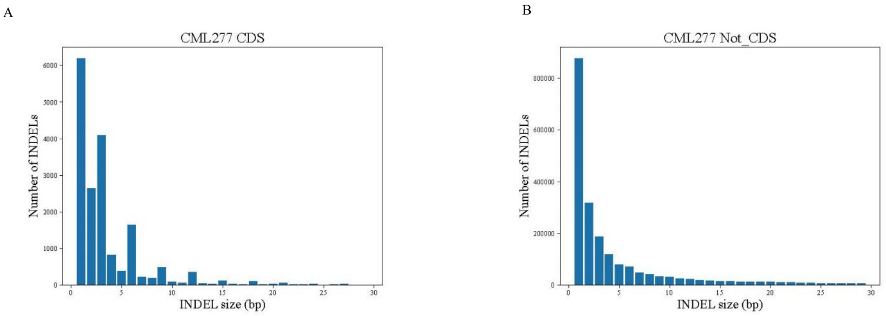

Fig. S65 Length distribution of INDELs identified solely by ACMGA in CDS and Non-CDS regions (CML277). Most frequent INDEL sizes differ between coding and non-coding regions. Multiple of three are much more common in coding sequences.

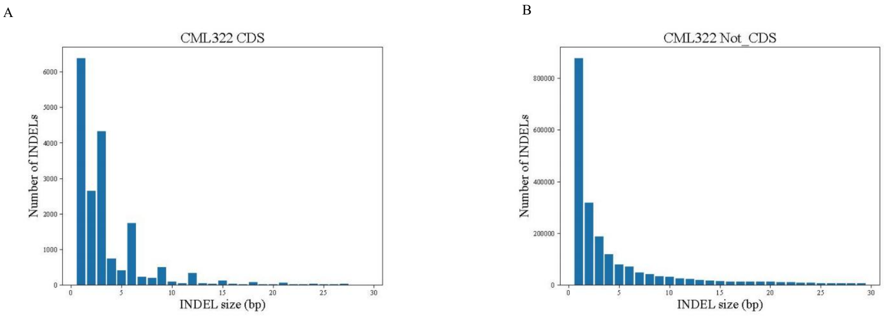

Fig. S66 Length distribution of INDELs identified solely by ACMGA in CDS and Non-CDS regions (CML322). Most frequent INDEL sizes differ between coding and non-coding regions. Multiple of three are much more common in coding sequences.

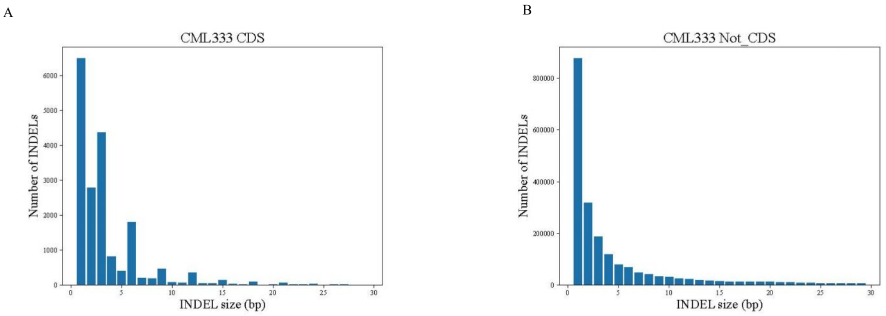

Fig. S67 Length distribution of INDELs identified solely by ACMGA in CDS and Non-CDS regions (CML333). Most frequent INDEL sizes differ between coding and non-coding regions. Multiple of three are much more common in coding sequences.

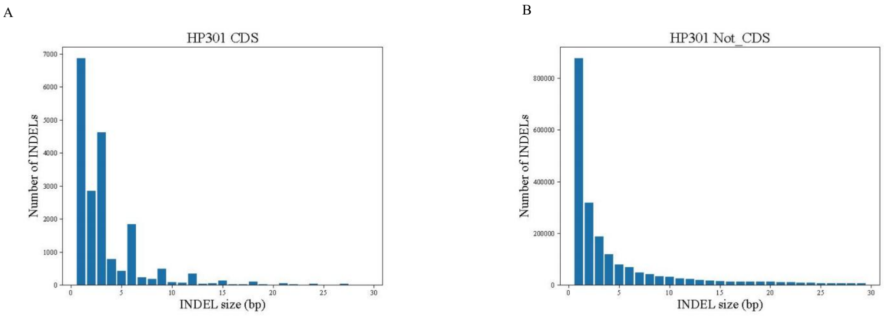

Fig. S68 Length distribution of INDELs identified solely by ACMGA in CDS and Non-CDS regions (HP301). Most frequent INDEL sizes differ between coding and non-coding regions. Multiple of three are much more common in coding sequences.

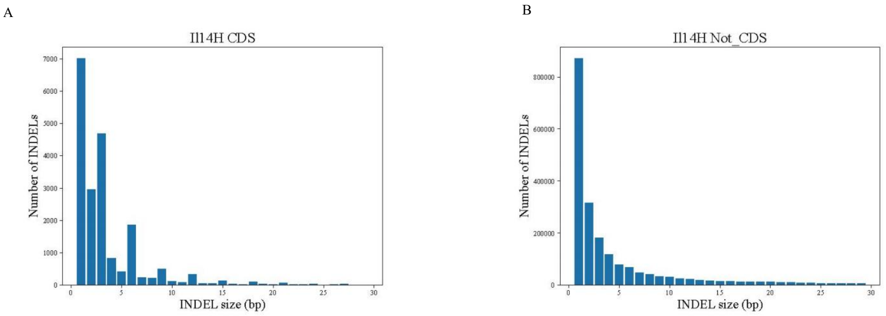

Fig. S69 Length distribution of INDELs identified solely by ACMGA in CDS and Non-CDS regions (Il14H). Most frequent INDEL sizes differ between coding and non-coding regions. Multiple of three are much more common in coding sequences.

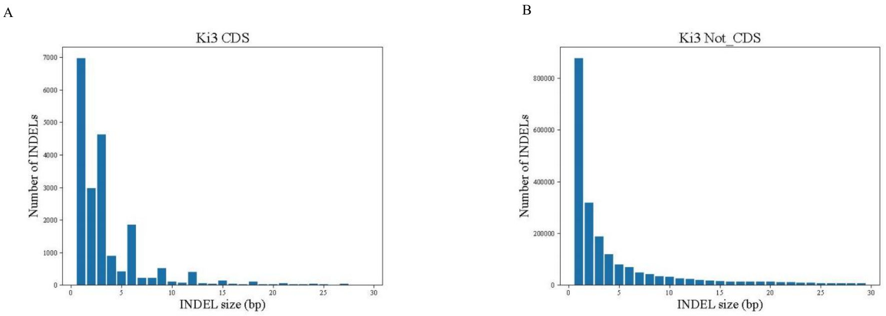

Fig. S70 Length distribution of INDELs identified solely by ACMGA in CDS and Non-CDS regions (Ki3). Most frequent INDEL sizes differ between coding and non-coding regions. Multiple of three are much more common in coding sequences.

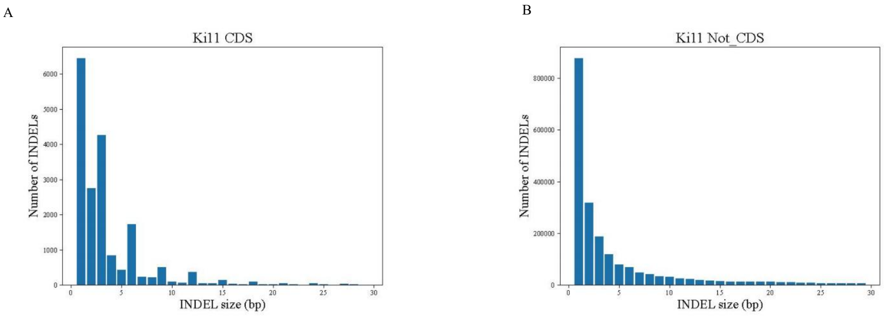

Fig. S71 Length distribution of INDELs identified solely by ACMGA in CDS and Non-CDS regions (Kill). Most frequent INDEL sizes differ between coding and non-coding regions. Multiple of three are much more common in coding sequences.

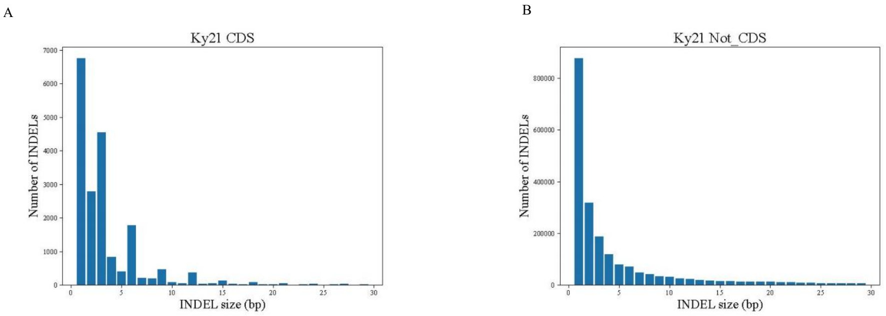

Fig. S72 Length distribution of INDELs identified solely by ACMGA in CDS and Non-CDS regions (Ky21). Most frequent INDEL sizes differ between coding and non-coding regions. Multiple of three are much more common in coding sequences.

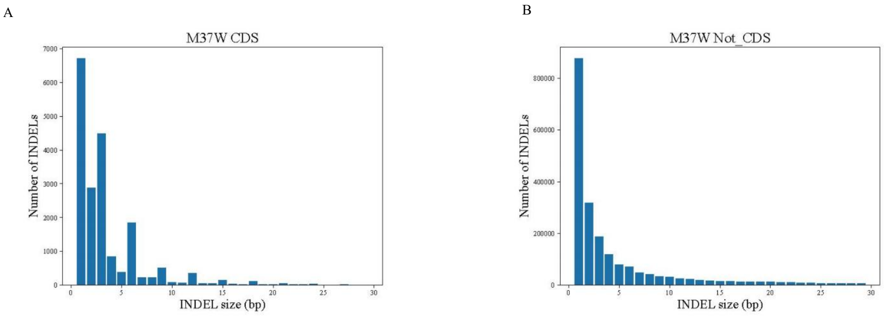

Fig. S73 Length distribution of INDELs identified solely by ACMGA in CDS and Non-CDS regions (M37W). Most frequent INDEL sizes differ between coding and non-coding regions. Multiple of three are much more common in coding sequences.

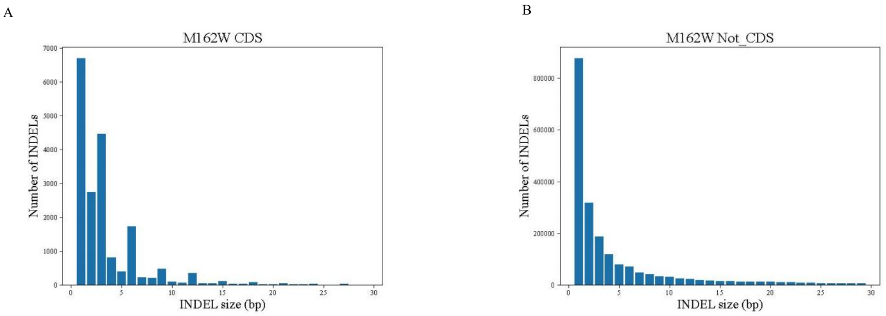

Fig. S74 Length distribution of INDELs identified solely by ACMGA in CDS and Non-CDS regions (M162W). Most frequent INDEL sizes differ between coding and non-coding regions. Multiple of three are much more common in coding sequences.

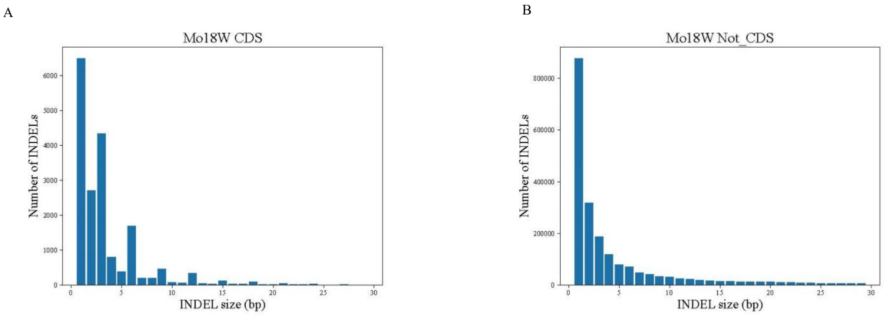

Fig. S75 Length distribution of INDELs identified solely by ACMGA in CDS and Non-CDS regions (Mo18W). Most frequent INDEL sizes differ between coding and non-coding regions. Multiple of three are much more common in coding sequences.

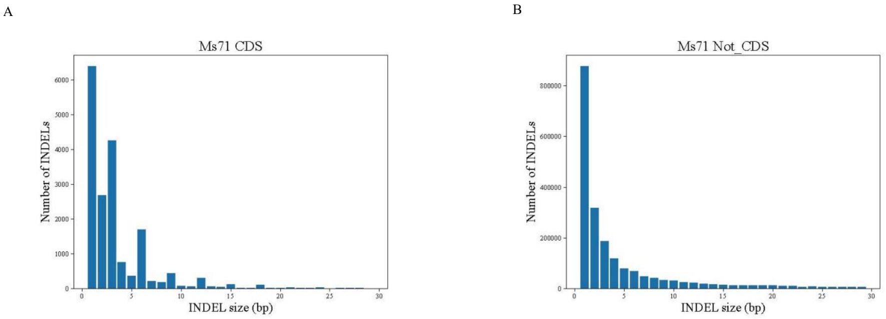

Fig. S76 Length distribution of INDELs identified solely by ACMGA in CDS and Non-CDS regions (MS71). Most frequent INDEL sizes differ between coding and non-coding regions. Multiple of three are much more common in coding sequences.

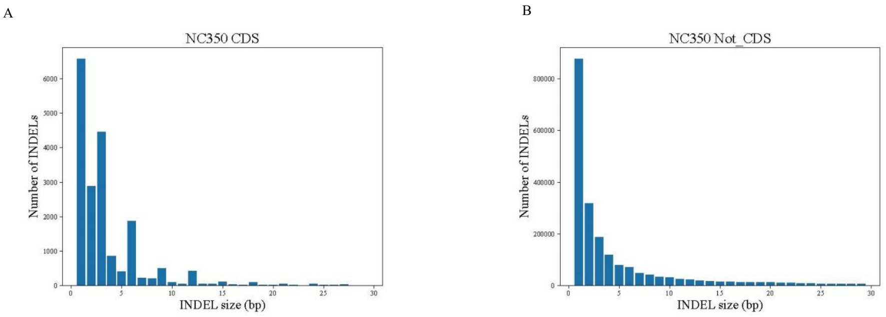

Fig. S77 Length distribution of INDELs identified solely by ACMGA in CDS and Non-CDS regions (NC350). Most frequent INDEL sizes differ between coding and non-coding regions. Multiple of three are much more common in coding sequences.

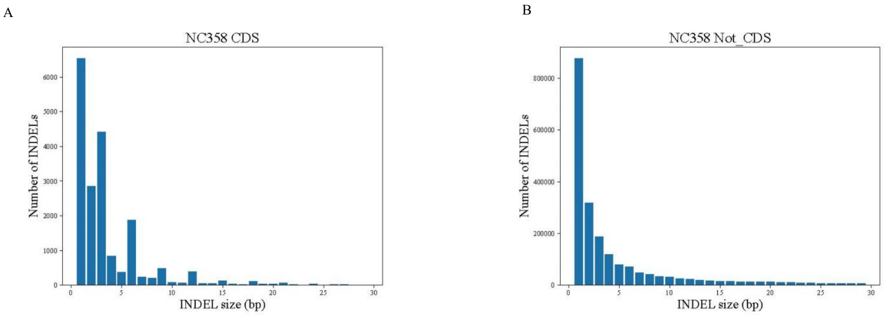

Fig. S78 Length distribution of INDELs identified solely by ACMGA in CDS and Non-CDS regions (NC358). Most frequent INDEL sizes differ between coding and non-coding regions. Multiple of three are much more common in coding sequences.

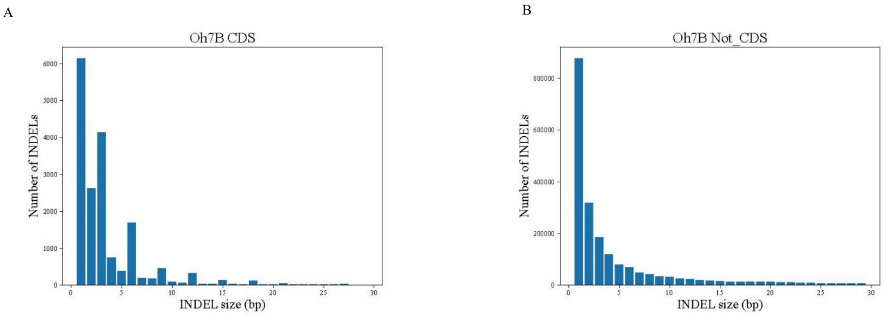

Fig. S79 Length distribution of INDELs identified solely by ACMGA in CDS and Non-CDS regions (Oh7B). Most frequent INDEL sizes differ between coding and non-coding regions. Multiple of three are much more common in coding sequences.

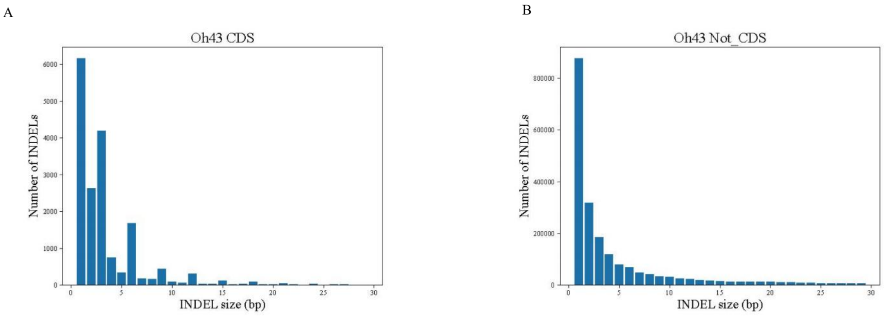

Fig. S80 Length distribution of INDELs identified solely by ACMGA in CDS and Non-CDS regions (Oh43). Most frequent INDEL sizes differ between coding and non-coding regions. Multiple of three are much more common in coding sequences.

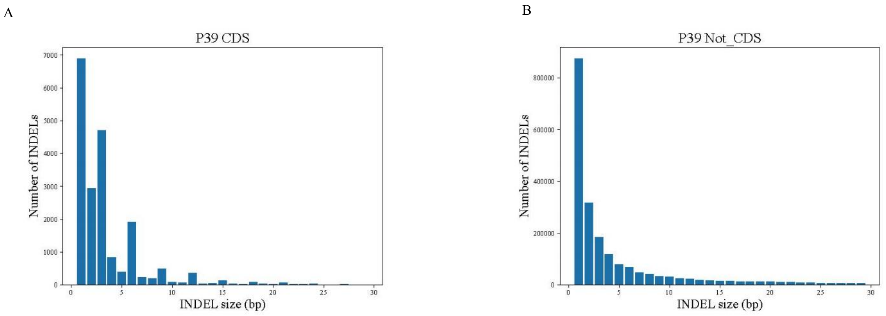

Fig. S81 Length distribution of INDELs identified solely by ACMGA in CDS and Non-CDS regions (P39). Most frequent INDEL sizes differ between coding and non-coding regions. Multiple of three are much more common in coding sequences.

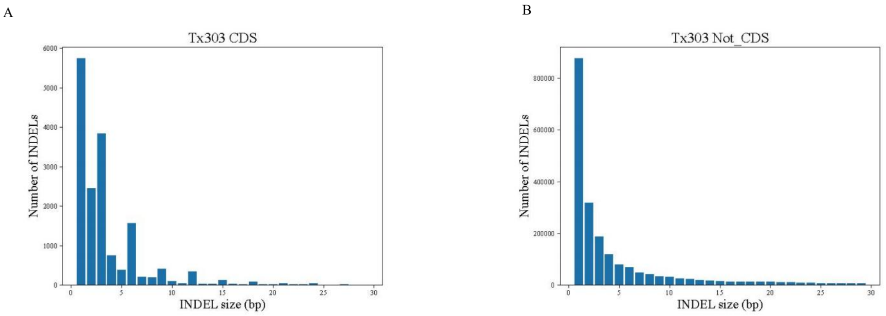

Fig. S82 Length distribution of INDELs identified solely by ACMGA in CDS and Non-CDS regions (Tx303). Most frequent INDEL sizes differ between coding and non-coding regions. Multiple of three are much more common in coding sequences.

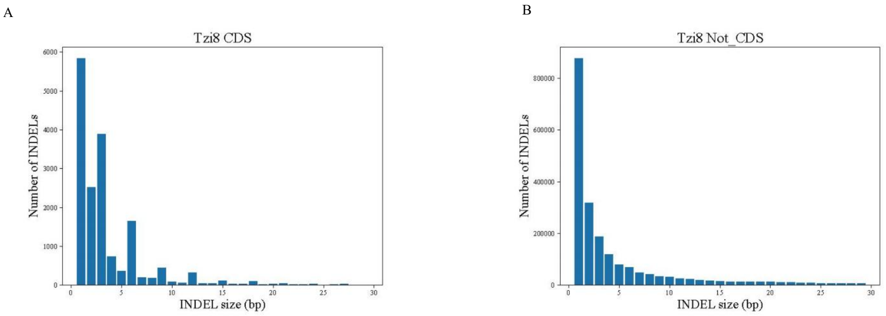

Fig. S83 Length distribution of INDELs identified solely by ACMGA in CDS and Non-CDS regions (Tzi8). Most frequent INDEL sizes differ between coding and non-coding regions. Multiple of three are much more common in coding sequences.

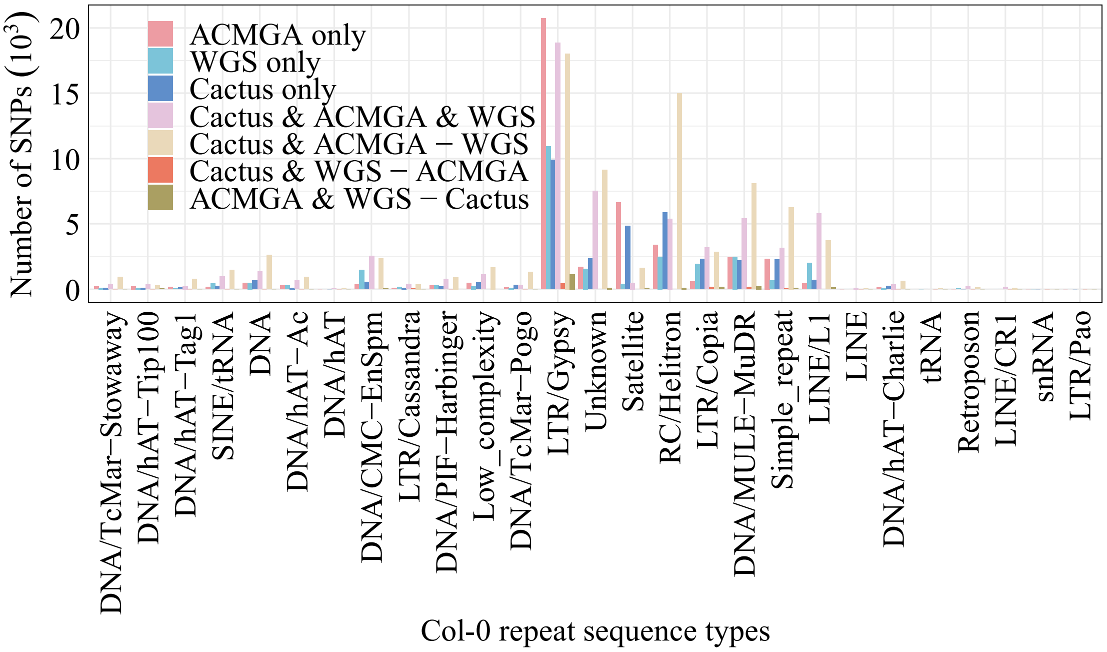

Fig. S84 The number of SNVs originating from repeated sequences between An-1 and Col-0 in different methods. The legends that correspond to the seven independent parts of the Venn diagram in Fig. 1A. The 'ACMGA only' category denotes SNVs that are exclusively identifiable by the ACMGA, remaining undetected by alternative methodologies. The 'WGS only' category denotes SNVs that are exclusively identifiable by the WGS, remaining undetected by alternative methodologies. The 'Cactus only' category denotes SNVs that are exclusively identifiable by the Cactus, remaining undetected by alternative methodologies. The 'Cactus&ACMGA&WGS' category denotes SNVs that are commonly identified by all three methods. The 'Cactus&ACMGA-WGS' category denotes SNVs discerned by both Cactus and ACMGA, but remain undetected by WGS. The 'ACMGA&WGS-Cactus' category denotes SNVs discerned by both ACMGA and WGS, but remain undetected by Cactus.

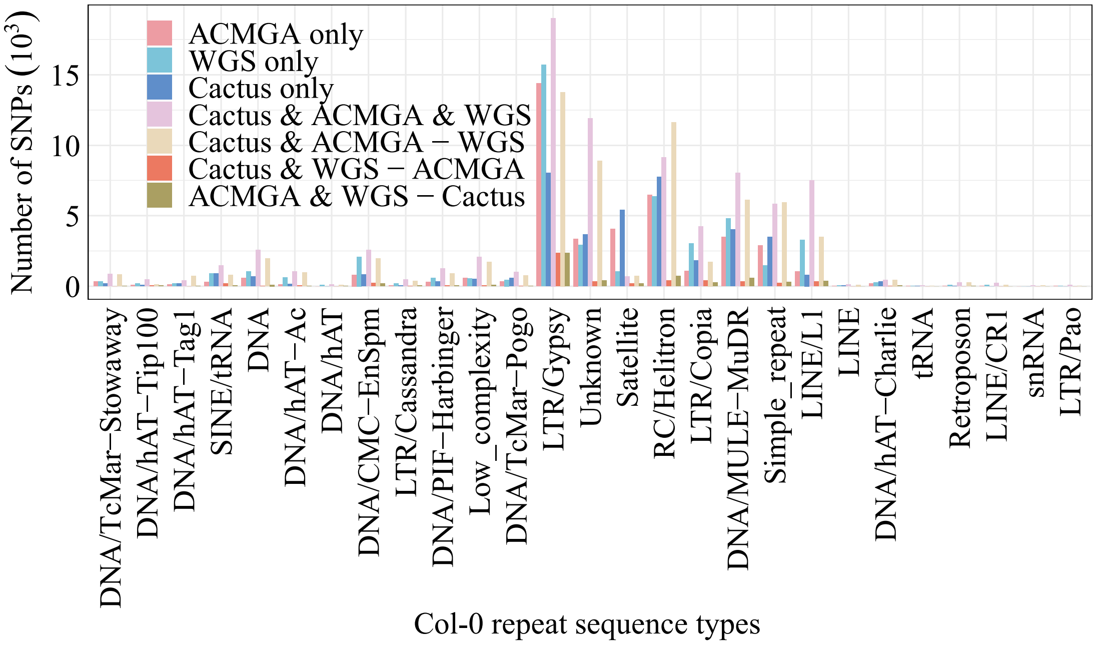

Fig. S85 The number of SNVs originating from repeated sequences between Cvi-0 and Col-0 in different methods. The legends that correspond to the seven independent parts of the Venn diagram in Fig. 2A. The 'ACMGA only' category denotes SNVs that are exclusively identifiable by the ACMGA, remaining undetected by alternative methodologies. The 'WGS only' category denotes SNVs that are exclusively identifiable by the WGS, remaining undetected by alternative methodologies. The 'Cactus only' category denotes SNVs that are exclusively identifiable by the Cactus, remaining undetected by alternative methodologies. The 'Cactus&ACMGA&WGS' category denotes SNVs that are commonly identified by all three methods. The 'Cactus&ACMGA-WGS' category denotes SNVs discerned by both Cactus and ACMGA, but remain undetected by WGS. The 'Cactus&WGS-ACMGA' category denotes SNVs discerned by both ACMGA and WGS, but remain undetected by Cactus.

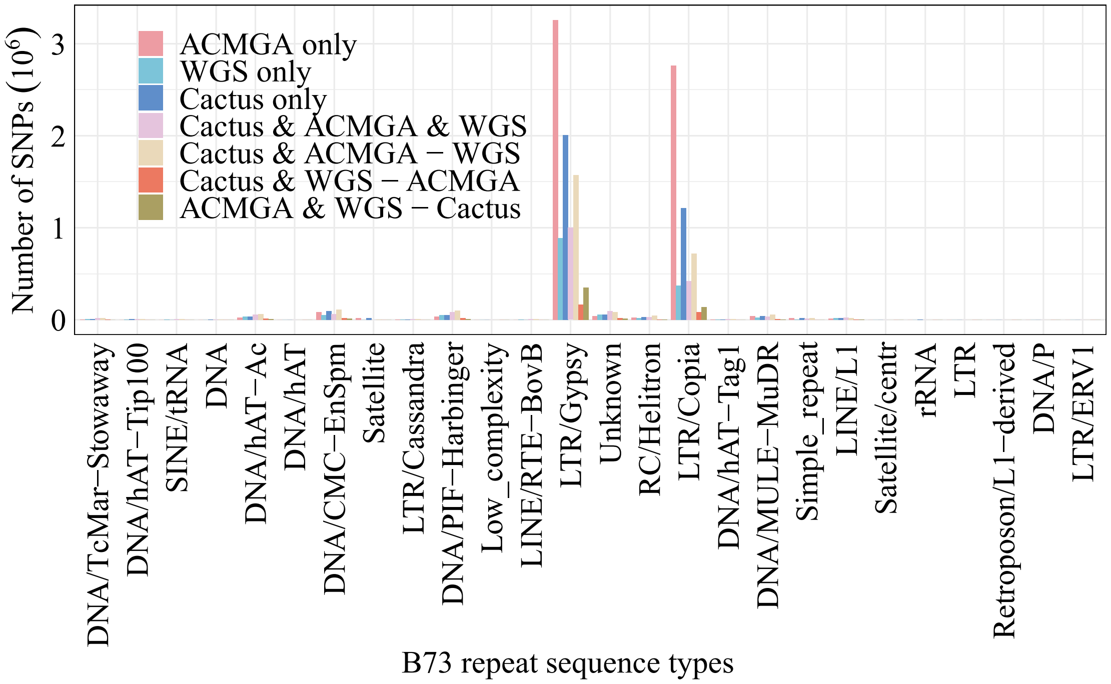

Fig. S86 The number of SNVs originating from repeated sequences between CML52 and B73 in different methods. The legends that correspond to the seven independent parts of the Venn diagram in Fig. 3A. The ‘ACMGA only’ category denotes SNVs that are exclusively identifiable by the ACMGA, remaining undetected by alternative methodologies. The ‘WGS only’ category denotes SNVs that are exclusively identifiable by the WGS, remaining undetected by alternative methodologies. The ‘Cactus only’ category denotes SNVs that are exclusively identifiable by the Cactus, remaining undetected by alternative methodologies. The ‘Cactus&ACMGA&WGS’ category denotes SNVs that are commonly identified by all three methods. The ‘Cactus&ACMGA-WGS’ category denotes SNVs discerned by both Cactus and ACMGA, but remain undetected by WGS. The ‘Cactus&WGS-ACMGA’ category denotes SNVs discerned by both ACMGA and WGS, but remain undetected by Cactus.

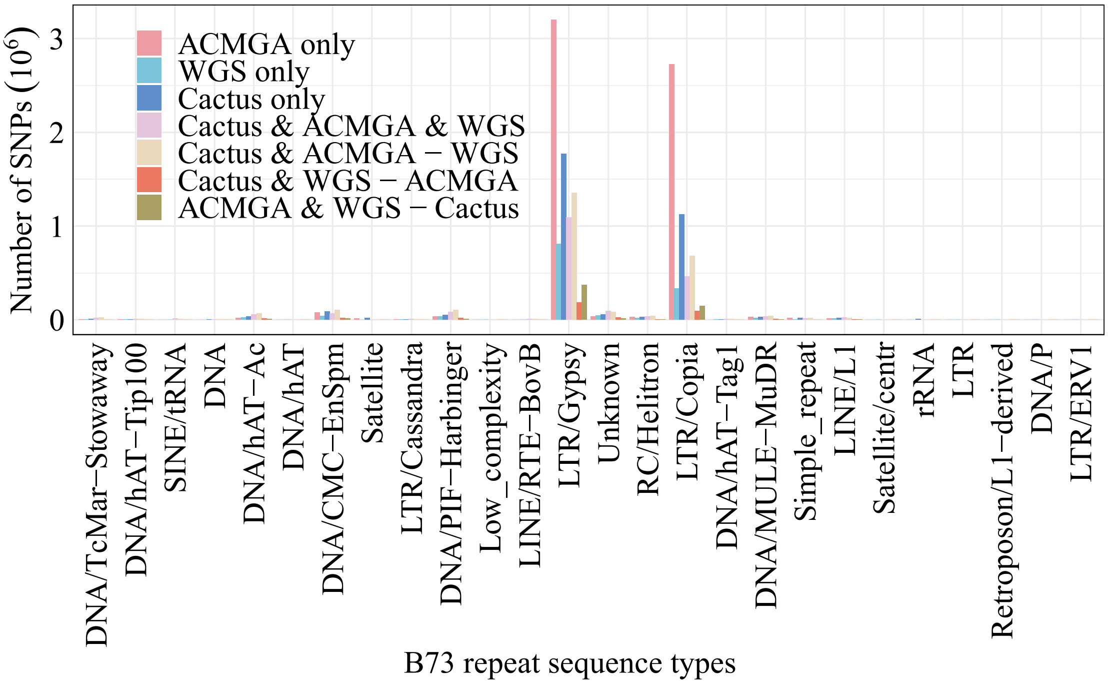

Fig. S87 The number of SNVs originating from repeated sequences between CML69 and B73 in different methods. The legends that correspond to the seven independent parts of the Venn diagram in Fig. 4A. The 'ACMGA only' category denotes SNVs that are exclusively identifiable by the ACMGA, remaining undetected by alternative methodologies. The 'WGS only' category denotes SNVs that are exclusively identifiable by the WGS, remaining undetected by alternative methodologies. The 'Cactus only' category denotes SNVs that are exclusively identifiable by the Cactus, remaining undetected by alternative methodologies. The 'Cactus&ACMGA&WGS' category denotes SNVs that are commonly identified by all three methods. The 'Cactus&ACMGA-WGS' category denotes SNVs discerned by both Cactus and ACMGA, but remain undetected by WGS. The 'Cactus&WGS-ACMGA' category denotes SNVs discerned by both ACMGA and WGS, but remain undetected by Cactus.

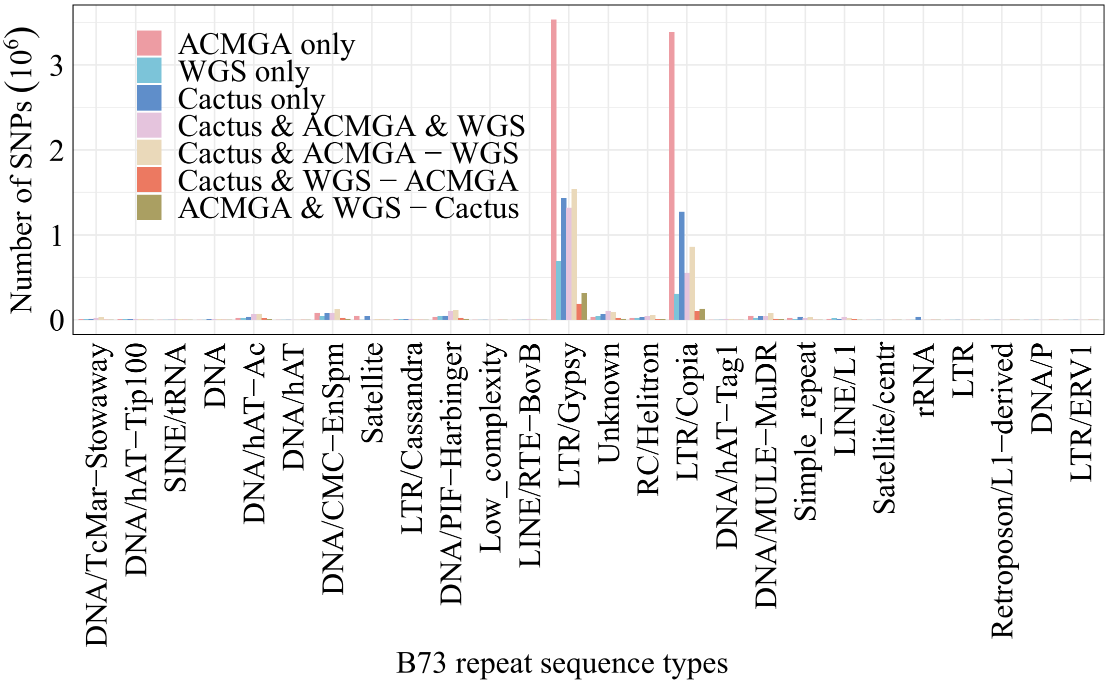

Fig. S88 The number of SNVs originating from repeated sequences between CML103 and B73 in different methods. The legends that correspond to the seven independent parts of the Venn diagram in Fig. 5A. The 'ACMGA only' category denotes SNVs that are exclusively identifiable by the ACMGA, remaining undetected by alternative methodologies. The 'WGS only' category denotes SNVs that are exclusively identifiable by the WGS, remaining undetected by alternative methodologies. The 'Cactus only' category denotes SNVs that are exclusively identifiable by the Cactus, remaining undetected by alternative methodologies. The 'Cactus&ACMGA&WGS' category denotes SNVs that are commonly identified by all three methods. The 'Cactus&ACMGA-WGS' category denotes SNVs discerned by both Cactus and ACMGA, but remain undetected by WGS. The 'Cactus&WGS-ACMGA' category denotes SNVs discerned by both ACMGA and WGS, but remain undetected by Cactus.

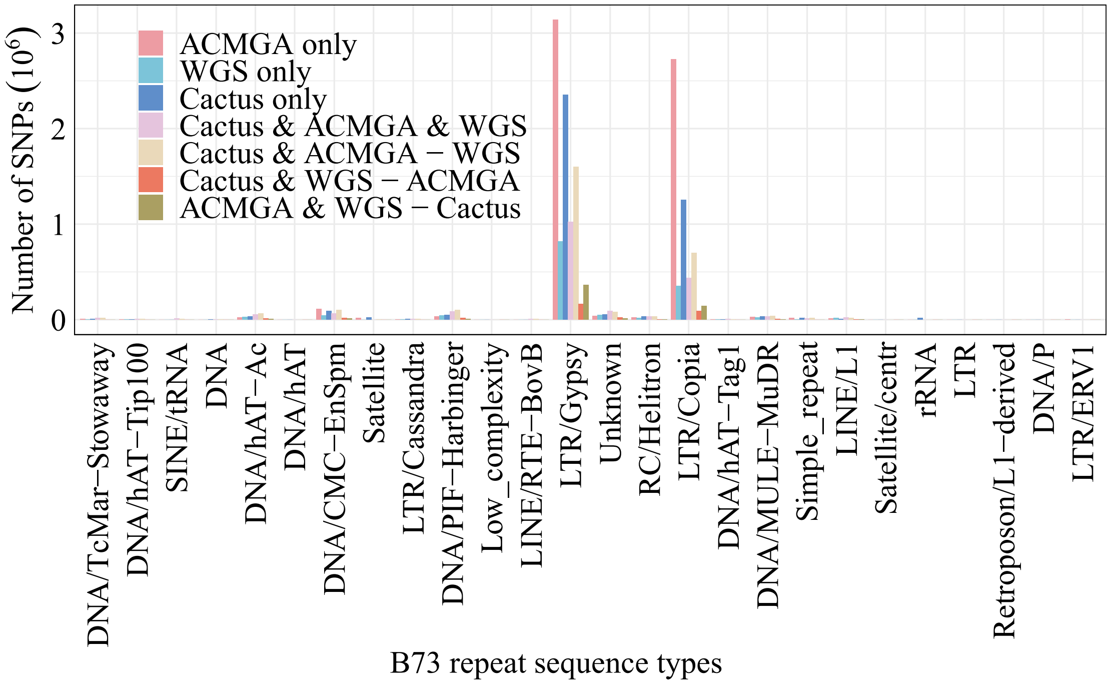

Fig. S89 The number of SNVs originating from repeated sequences between CML228 and B73 in different methods. The legends that correspond to the seven independent parts of the Venn diagram in Fig. 6A. The 'ACMGA only' category denotes SNVs that are exclusively identifiable by the ACMGA, remaining undetected by alternative methodologies. The 'WGS only' category denotes SNVs that are exclusively identifiable by the WGS, remaining undetected by alternative methodologies. The 'Cactus only' category denotes SNVs that are exclusively identifiable by the Cactus, remaining undetected by alternative methodologies. The 'Cactus&ACMGA&WGS' category denotes SNVs that are commonly identified by all three methods. The 'Cactus&ACMGA-WGS' category denotes SNVs discerned by both Cactus and ACMGA, but remain undetected by WGS. The 'Cactus&WGS-ACMGA' category denotes SNVs discerned by both ACMGA and WGS, but remain undetected by Cactus.

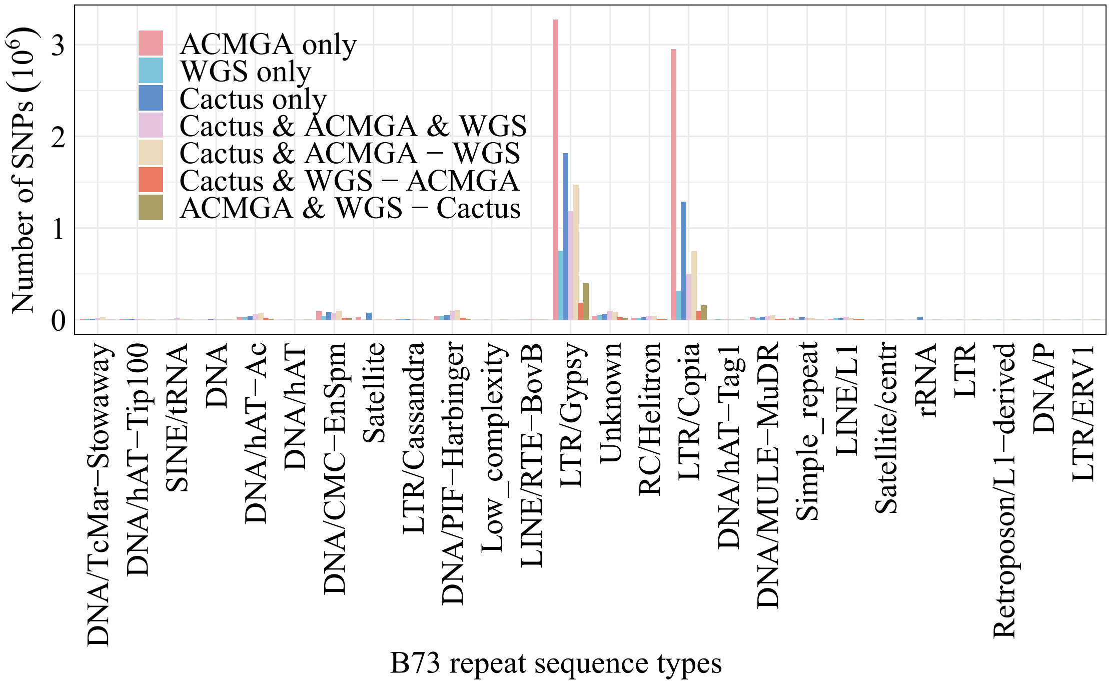

Fig. S90 The number of SNVs originating from repeated sequences between CML247 and B73 in different methods. The legends that correspond to the seven independent parts of the Venn diagram in Fig. 7A. The 'ACMGA only' category denotes SNVs that are exclusively identifiable by the ACMGA, remaining undetected by alternative methodologies. The 'WGS only' category denotes SNVs that are exclusively identifiable by the WGS, remaining undetected by alternative methodologies. The 'Cactus only' category denotes SNVs that are exclusively identifiable by the Cactus, remaining undetected by alternative methodologies. The 'Cactus&ACMGA&WGS' category denotes SNVs that are commonly identified by all three methods. The 'Cactus&ACMGA-WGS' category denotes SNVs discerned by both Cactus and ACMGA, but remain undetected by WGS. The 'Cactus&WGS-ACMGA' category denotes SNVs discerned by both ACMGA and WGS, but remain undetected by Cactus.

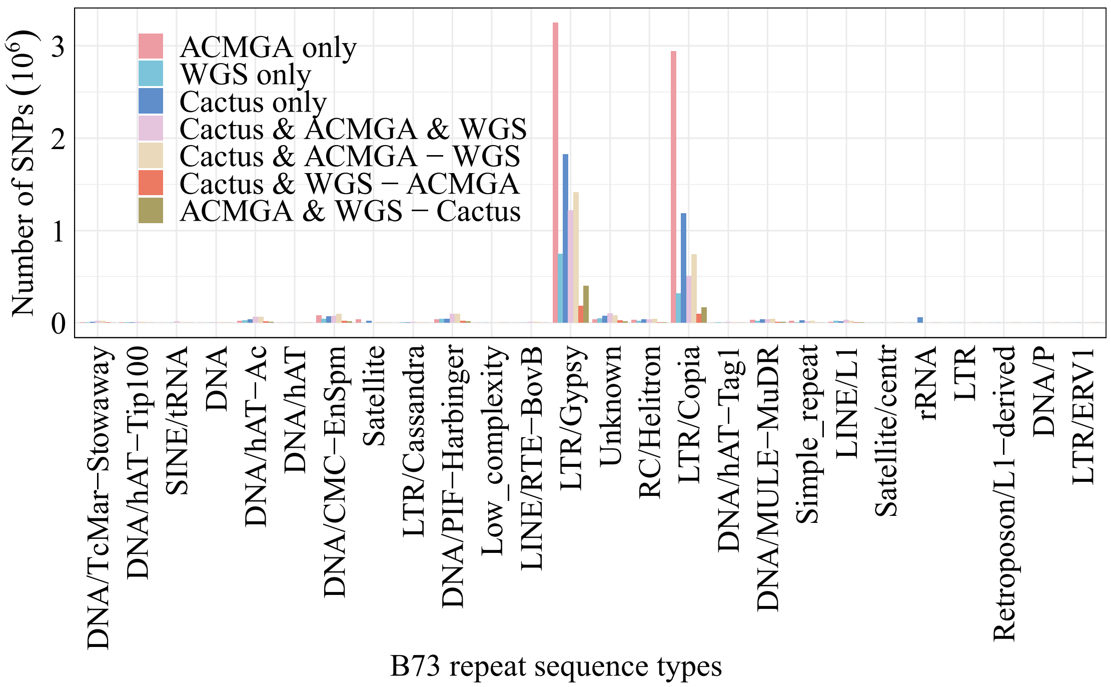

Fig. S91 The number of SNVs originating from repeated sequences between CML277 and B73 in different methods. The legends that correspond to the seven independent parts of the Venn diagram in Fig. 8A. The 'ACMGA only' category denotes SNVs that are exclusively identifiable by the ACMGA, remaining undetected by alternative methodologies. The 'WGS only' category denotes SNVs that are exclusively identifiable by the WGS, remaining undetected by alternative methodologies. The 'Cactus only' category denotes SNVs that are exclusively identifiable by the Cactus, remaining undetected by alternative methodologies. The 'Cactus&ACMGA&WGS' category denotes SNVs that are commonly identified by all three methods. The 'Cactus&ACMGA-WGS' category denotes SNVs discerned by both Cactus and ACMGA, but remain undetected by WGS. The 'Cactus&WGS-ACMGA' category denotes SNVs discerned by both ACMGA and WGS, but remain undetected by Cactus.

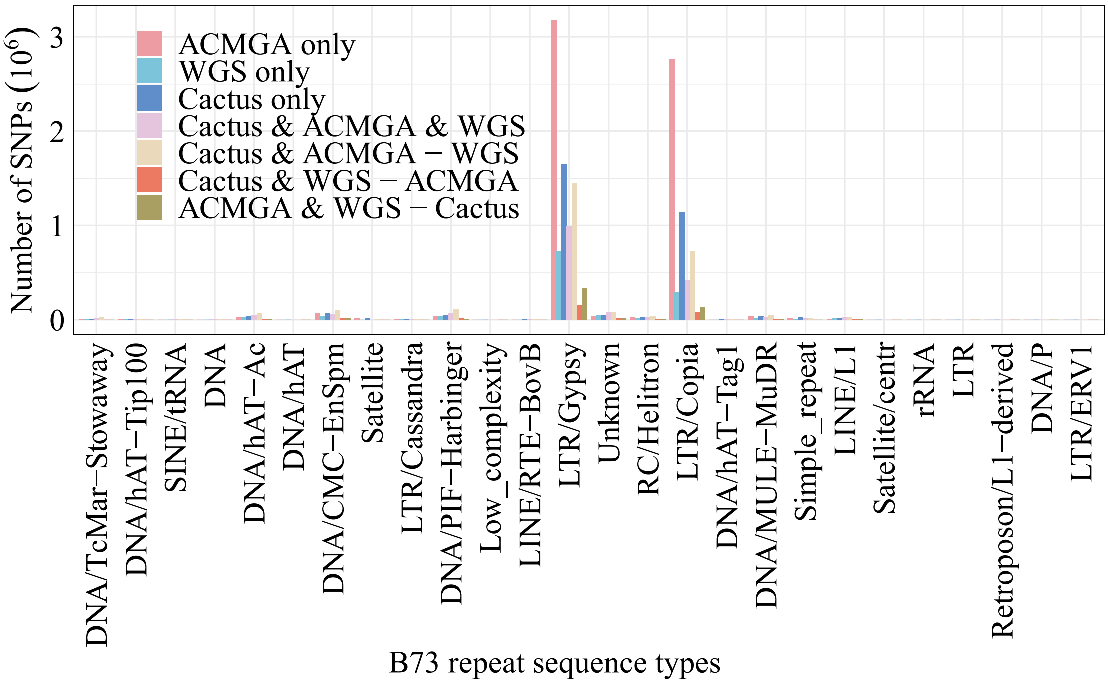

Fig. S92 The number of SNVs originating from repeated sequences between CML322 and B73 in different methods.. The legends that correspond to the seven independent parts of the Venn diagram in Fig. 9A. The ‘ACMGA only’ category denotes SNVs that are exclusively identifiable by the ACMGA, remaining undetected by alternative methodologies. The ‘WGS only’ category denotes SNVs that are exclusively identifiable by the WGS, remaining undetected by alternative methodologies. The ‘Cactus only’ category denotes SNVs that are exclusively identifiable by the Cactus, remaining undetected by alternative methodologies. The ‘Cactus&ACMGA&WGS’ category denotes SNVs that are commonly identified by all three methods. The ‘Cactus&ACMGA-WGS’ category denotes SNVs discerned by both Cactus and ACMGA, but remain undetected by WGS. The ‘Cactus&WGS-ACMGA’ category denotes SNVs discerned by both ACMGA and WGS, but remain undetected by Cactus.

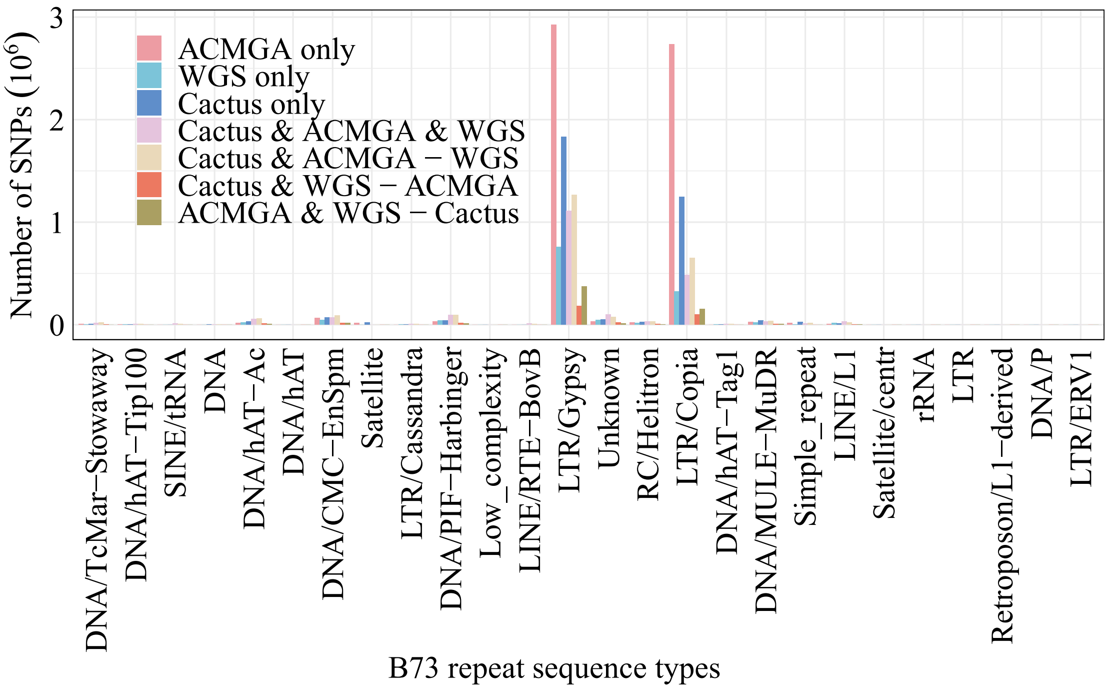

Fig. S93 The number of SNVs originating from repeated sequences between CML333 and B73 in different methods. The legends that correspond to the seven independent parts of the Venn diagram in Fig. 10A. The 'ACMGA only' category denotes SNVs that are exclusively identifiable by the ACMGA, remaining undetected by alternative methodologies. The 'WGS only' category denotes SNVs that are exclusively identifiable by the WGS, remaining undetected by alternative methodologies. The 'Cactus only' category denotes SNVs that are exclusively identifiable by the Cactus, remaining undetected by alternative methodologies. The 'Cactus&ACMGA&WGS' category denotes SNVs that are commonly identified by all three methods. The 'Cactus&ACMGA-WGS' category denotes SNVs discerned by both Cactus and ACMGA, but remain undetected by WGS. The 'Cactus&WGS-ACMGA' category denotes SNVs discerned by both ACMGA and WGS, but remain undetected by Cactus.

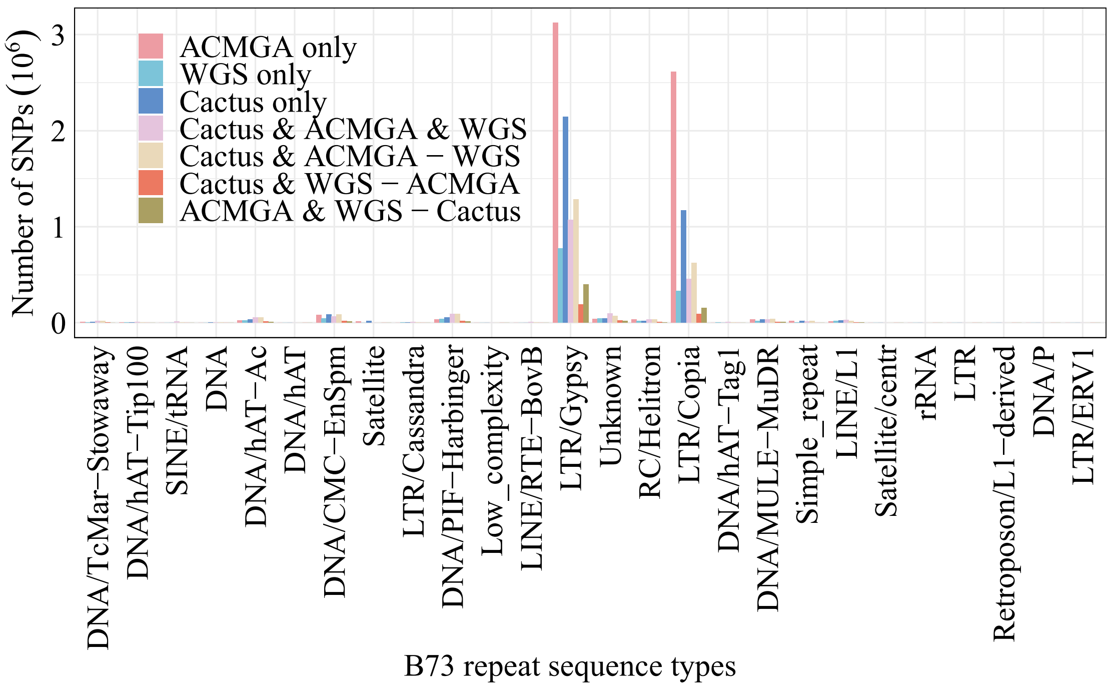

Fig. S94 The number of SNVs originating from repeated sequences between HP301 and B73 in different methods. The legends that correspond to the seven independent parts of the Venn diagram in Fig. 11A. The 'ACMGA only' category denotes SNVs that are exclusively identifiable by the ACMGA, remaining undetected by alternative methodologies. The 'WGS only' category denotes SNVs that are exclusively identifiable by the WGS, remaining undetected by alternative methodologies. The 'Cactus only' category denotes SNVs that are exclusively identifiable by the Cactus, remaining undetected by alternative methodologies. The 'Cactus&ACMGA&WGS' category denotes SNVs that are commonly identified by all three methods. The 'Cactus&ACMGA-WGS' category denotes SNVs discerned by both Cactus and ACMGA, but remain undetected by WGS. The 'Cactus&WGS-ACMGA' category denotes SNVs discerned by both ACMGA and WGS, but remain undetected by Cactus.

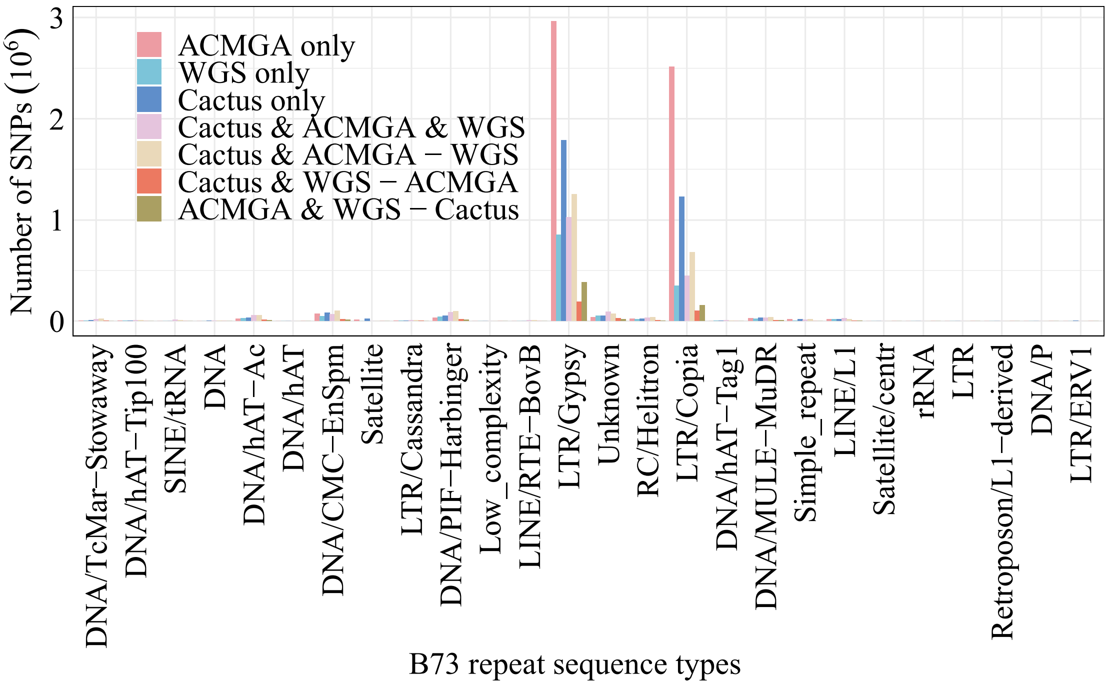

Fig. S95 The number of SNVs originating from repeated sequences between Il14H and B73 in different methods. The legends that correspond to the seven independent parts of the Venn diagram in Fig. 12A. The 'ACMGA only' category denotes SNVs that are exclusively identifiable by the ACMGA, remaining undetected by alternative methodologies. The 'WGS only' category denotes SNVs that are exclusively identifiable by the WGS, remaining undetected by alternative methodologies. The 'Cactus only' category denotes SNVs that are exclusively identifiable by the Cactus, remaining undetected by alternative methodologies. The 'Cactus&ACMGA&WGS' category denotes SNVs that are commonly identified by all three methods. The 'Cactus&ACMGA-WGS' category denotes SNVs discerned by both Cactus and ACMGA, but remain undetected by WGS. The 'Cactus&WGS-ACMGA' category denotes SNVs discerned by both ACMGA and WGS, but remain undetected by Cactus.

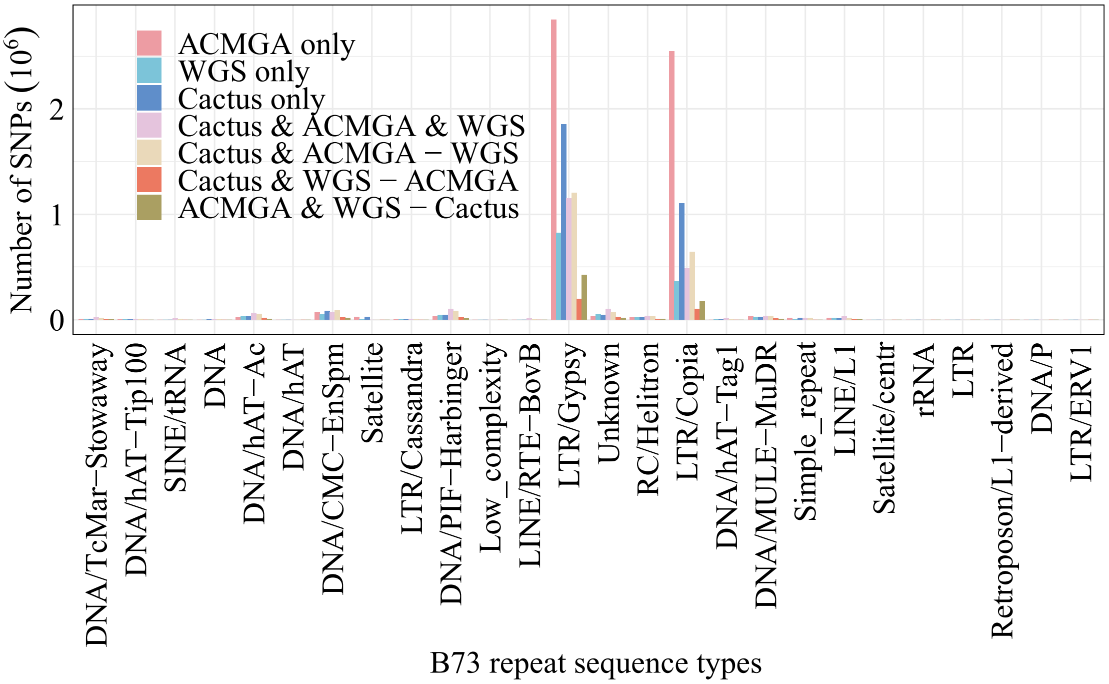

Fig. S96 The number of SNVs originating from repeated sequences between Ki3 and B73 in different methods. The legends that correspond to the seven independent parts of the Venn diagram in Fig. 13A. The 'ACMGA only' category denotes SNVs that are exclusively identifiable by the ACMGA, remaining undetected by alternative methodologies. The 'WGS only' category denotes SNVs that are exclusively identifiable by the WGS, remaining undetected by alternative methodologies. The 'Cactus only' category denotes SNVs that are exclusively identifiable by the Cactus, remaining undetected by alternative methodologies. The 'Cactus&ACMGA&WGS' category denotes SNVs that are commonly identified by all three methods. The 'Cactus&ACMGA-WGS' category denotes SNVs discerned by both Cactus and ACMGA, but remain undetected by WGS. The 'Cactus&WGS-ACMGA' category denotes SNVs discerned by both ACMGA and WGS, but remain undetected by Cactus.

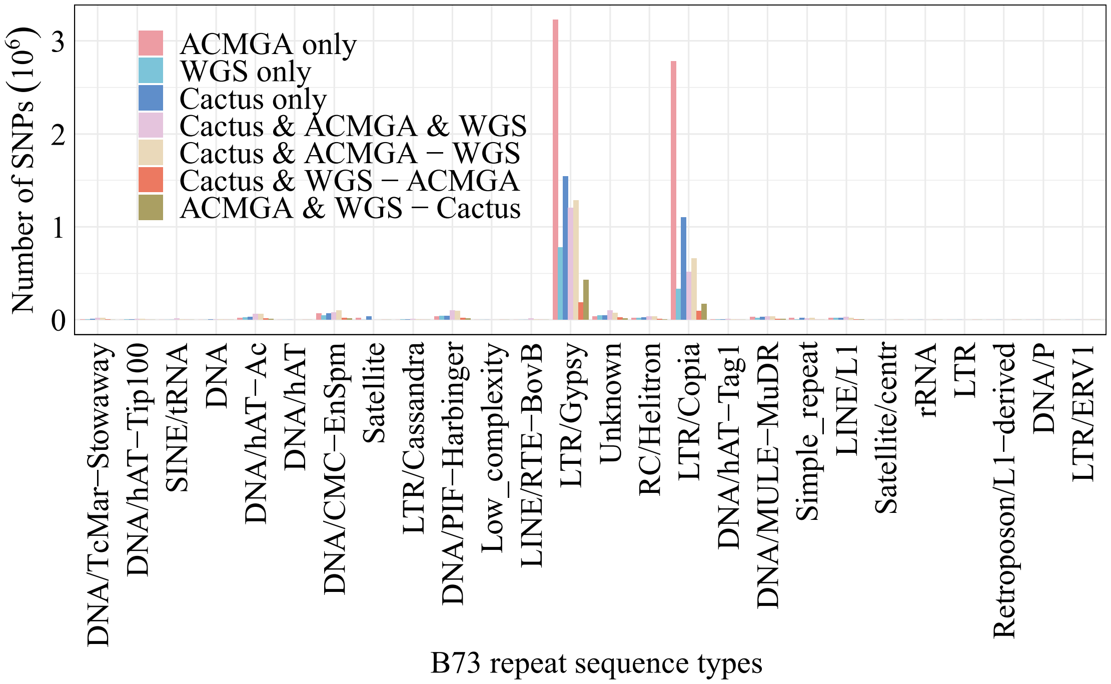

Fig. S97 The number of SNVs originating from repeated sequences between Kill and B73 in different methods. The legends that correspond to the seven independent parts of the Venn diagram in Fig. 14A. The ‘ACMGA only’ category denotes SNVs that are exclusively identifiable by the ACMGA, remaining undetected by alternative methodologies. The ‘WGS only’ category denotes SNVs that are exclusively identifiable by the WGS, remaining undetected by alternative methodologies. The ‘Cactus only’ category denotes SNVs that are exclusively identifiable by the Cactus, remaining undetected by alternative methodologies. The ‘Cactus&ACMGA&WGS’ category denotes SNVs that are commonly identified by all three methods. The ‘Cactus&ACMGA-WGS’ category denotes SNVs discerned by both Cactus and ACMGA, but remain undetected by WGS. The ‘Cactus&WGS-ACMGA’ category denotes SNVs discerned by both ACMGA and WGS, but remain undetected by Cactus.

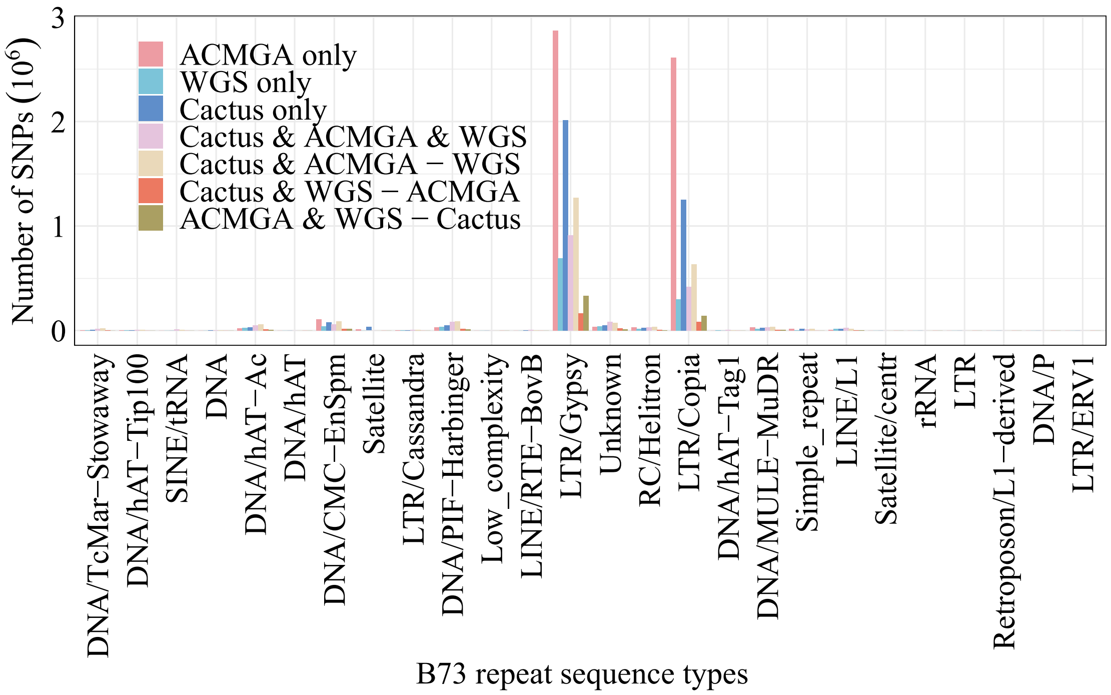

Fig. S98 The number of SNVs originating from repeated sequences between Ky21 and B73 in different methods.. The legends that correspond to the seven independent parts of the Venn diagram in Fig. 15A. The 'ACMGA only' category denotes SNVs that are exclusively identifiable by the ACMGA, remaining undetected by alternative methodologies. The 'WGS only' category denotes SNVs that are exclusively identifiable by the WGS, remaining undetected by alternative methodologies. The 'Cactus only' category denotes SNVs that are exclusively identifiable by the Cactus, remaining undetected by alternative methodologies. The 'Cactus&ACMGA&WGS' category denotes SNVs that are commonly identified by all three methods. The 'Cactus&ACMGA-WGS' category denotes SNVs discerned by both Cactus and ACMGA, but remain undetected by WGS. The 'Cactus&WGS-ACMGA' category denotes SNVs discerned by both ACMGA and WGS, but remain undetected by Cactus.

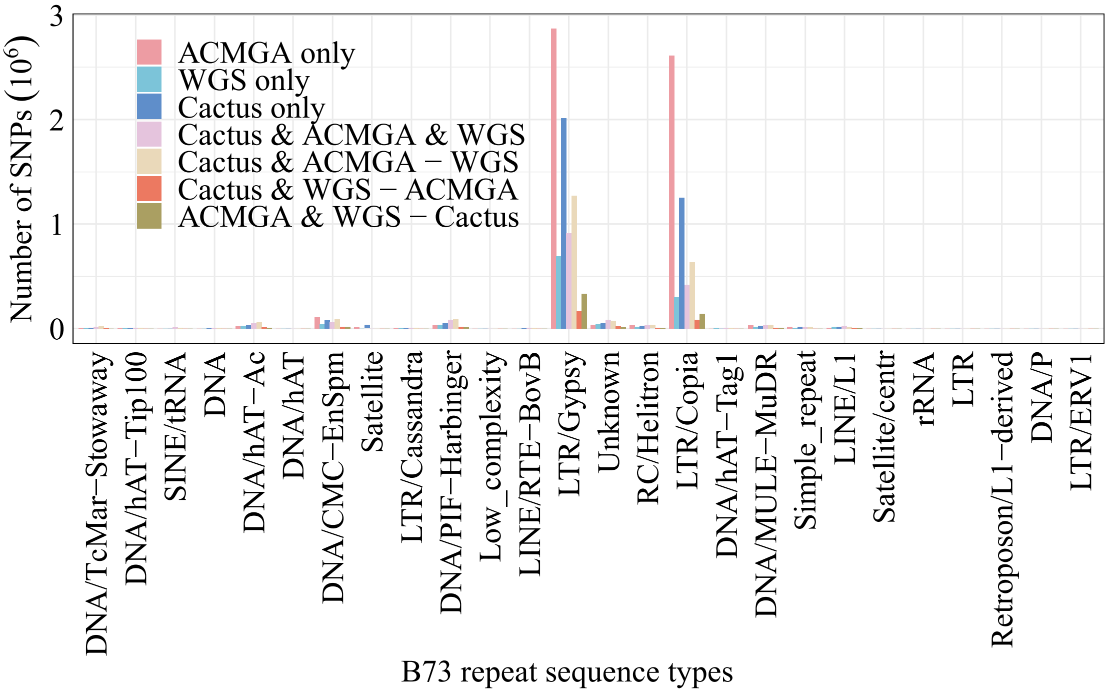

Fig. S99 The number of SNVs originating from repeated sequences between M37W and B73 in different methods. The legends that correspond to the seven independent parts of the Venn diagram in Fig. 16A. The 'ACMGA only' category denotes SNVs that are exclusively identifiable by the ACMGA, remaining undetected by alternative methodologies. The 'WGS only' category denotes SNVs that are exclusively identifiable by the WGS, remaining undetected by alternative methodologies. The 'Cactus only' category denotes SNVs that are exclusively identifiable by the Cactus, remaining undetected by alternative methodologies. The 'Cactus&ACMGA&WGS' category denotes SNVs that are commonly identified by all three methods. The 'Cactus&ACMGA-WGS' category denotes SNVs discerned by both Cactus and ACMGA, but remain undetected by WGS. The 'Cactus&WGS-ACMGA' category denotes SNVs discerned by both ACMGA and WGS, but remain undetected by Cactus.

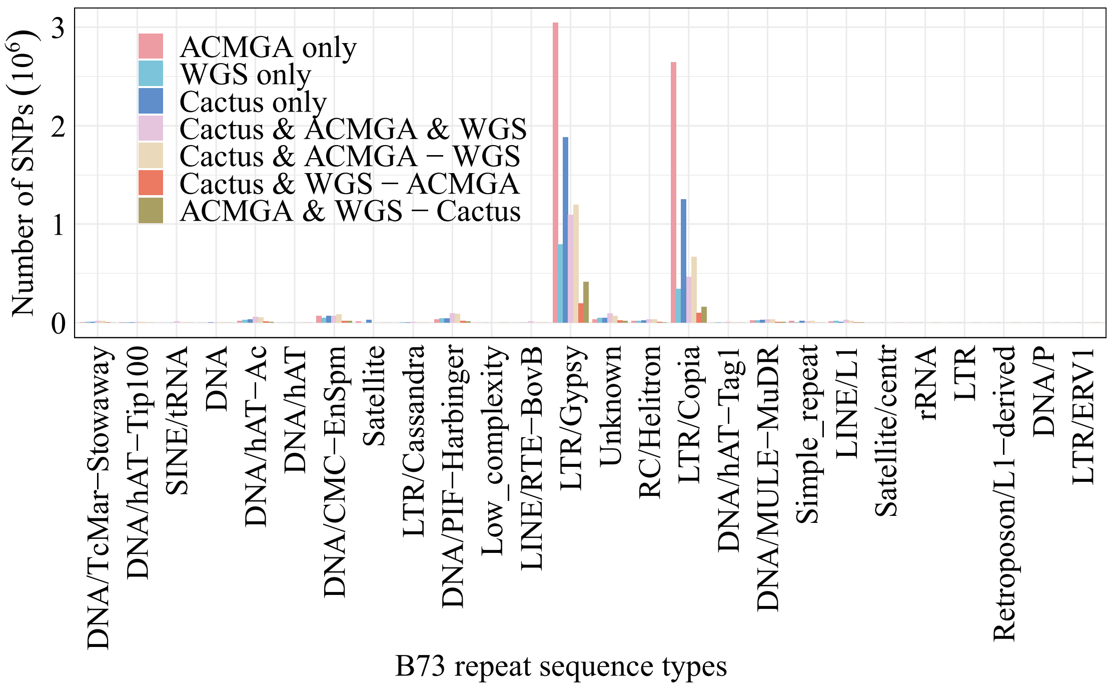

Fig. S100 The number of SNVs originating from repeated sequences between M162W and B73 in different methods.. The legends that correspond to the seven independent parts of the Venn diagram in Fig. 17A. The 'ACMGA only' category denotes SNVs that are exclusively identifiable by the ACMGA, remaining undetected by alternative methodologies. The 'WGS only' category denotes SNVs that are exclusively identifiable by the WGS, remaining undetected by alternative methodologies. The 'Cactus only' category denotes SNVs that are exclusively identifiable by the Cactus, remaining undetected by alternative methodologies. The 'Cactus&ACMGA&WGS' category denotes SNVs that are commonly identified by all three methods. The 'Cactus&ACMGA-WGS' category denotes SNVs discerned by both Cactus and ACMGA, but remain undetected by WGS. The 'Cactus&WGS-ACMGA' category denotes SNVs discerned by both ACMGA and WGS, but remain undetected by Cactus.

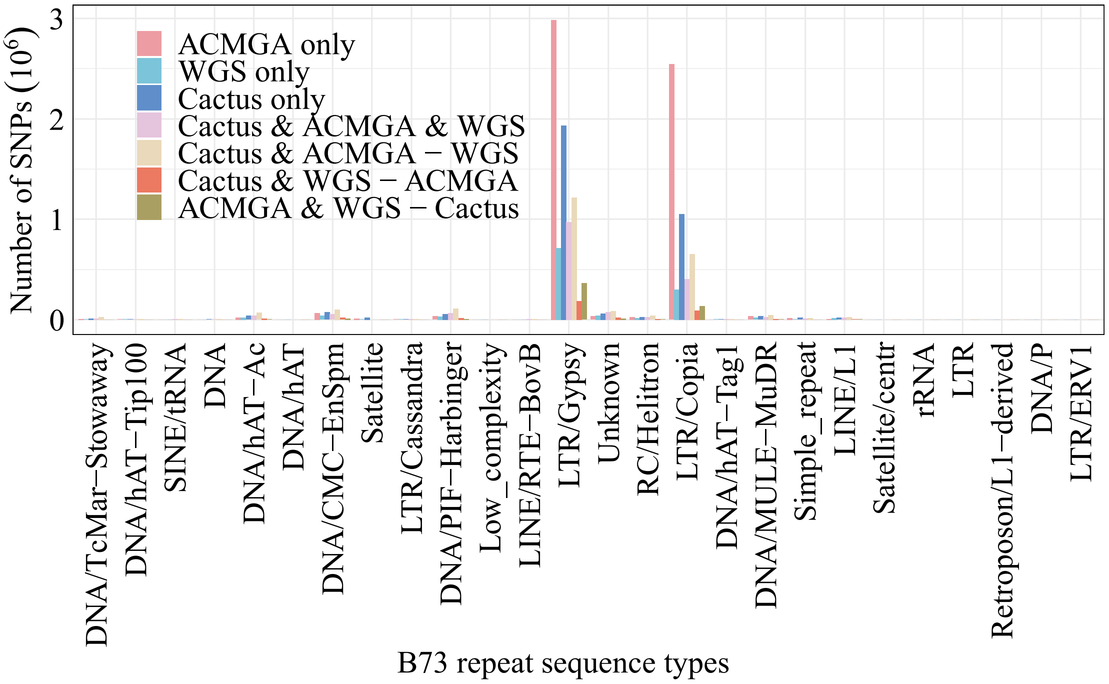

Fig. S101 The number of SNVs originating from repeated sequences between Mo18W and B73 in different methods. The legends that correspond to the seven independent parts of the Venn diagram in Fig. 18A. The 'ACMGA only' category denotes SNVs that are exclusively identifiable by the ACMGA, remaining undetected by alternative methodologies. The 'WGS only' category denotes SNVs that are exclusively identifiable by the WGS, remaining undetected by alternative methodologies. The 'Cactus only' category denotes SNVs that are exclusively identifiable by the Cactus, remaining undetected by alternative methodologies. The 'Cactus&ACMGA&WGS' category denotes SNVs that are commonly identified by all three methods. The 'Cactus&ACMGA-WGS' category denotes SNVs discerned by both Cactus and ACMGA, but remain undetected by WGS. The 'Cactus&WGS-ACMGA' category denotes SNVs discerned by both ACMGA and WGS, but remain undetected by Cactus.

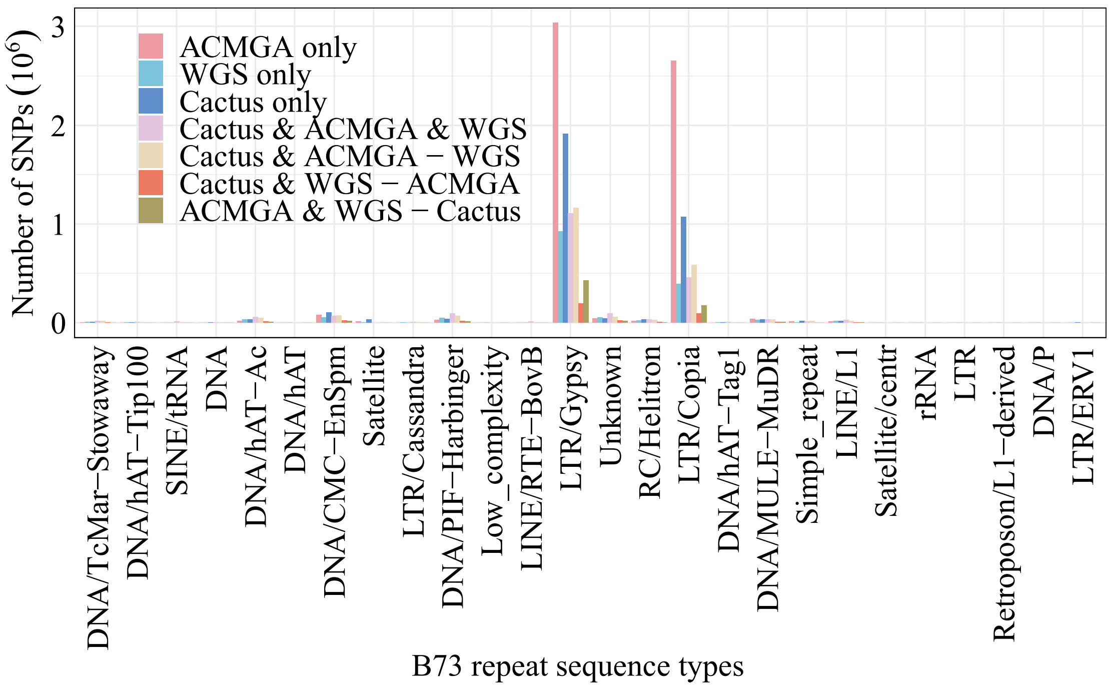

Fig. S102 The number of SNVs originating from repeated sequences between Ms71 and B73 in different methods. The legends that correspond to the seven independent parts of the Venn diagram in Fig. 19A. The 'ACMGA only' category denotes SNVs that are exclusively identifiable by the ACMGA, remaining undetected by alternative methodologies. The 'WGS only' category denotes SNVs that are exclusively identifiable by the WGS, remaining undetected by alternative methodologies. The 'Cactus only' category denotes SNVs that are exclusively identifiable by the Cactus, remaining undetected by alternative methodologies. The 'Cactus&ACMGA&WGS' category denotes SNVs that are commonly identified by all three methods. The 'Cactus&ACMGA-WGS' category denotes SNVs discerned by both Cactus and ACMGA, but remain undetected by WGS. The 'Cactus&WGS-ACMGA' category denotes SNVs discerned by both ACMGA and WGS, but remain undetected by Cactus.

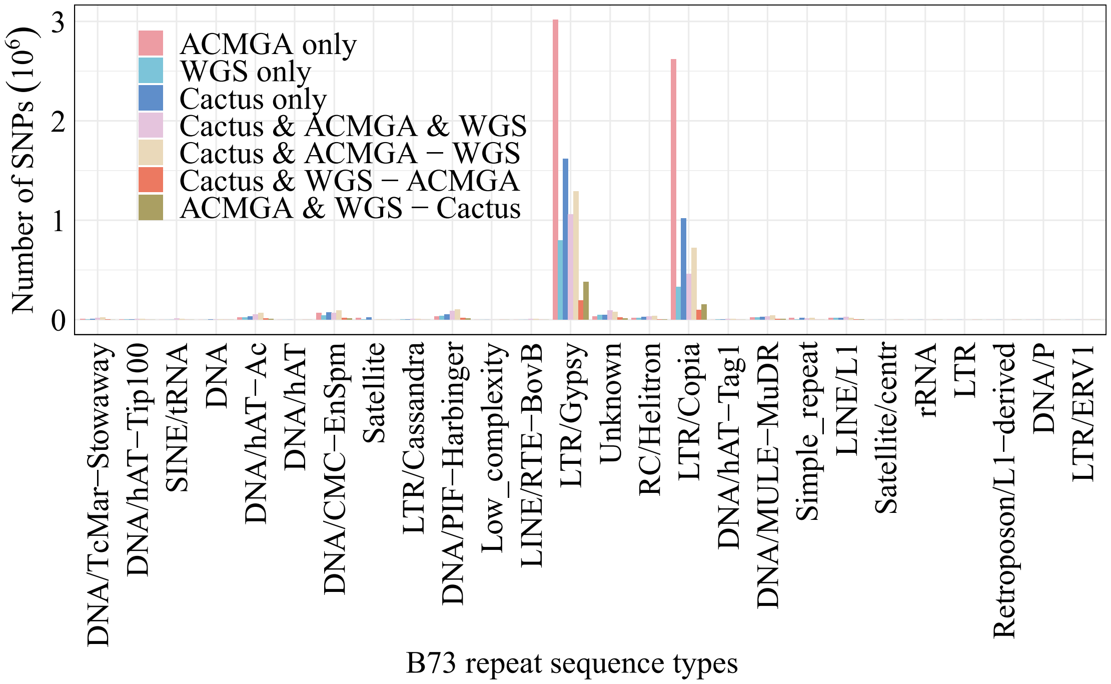

Fig. S103 The number of SNVs originating from repeated sequences between NC350 and B73 in different methods. The legends that correspond to the seven independent parts of the Venn diagram in Fig. 20A. The 'ACMGA only' category denotes SNVs that are exclusively identifiable by the ACMGA, remaining undetected by alternative methodologies. The 'WGS only' category denotes SNVs that are exclusively identifiable by the WGS, remaining undetected by alternative methodologies. The 'Cactus only' category denotes SNVs that are exclusively identifiable by the Cactus, remaining undetected by alternative methodologies. The 'Cactus&ACMGA&WGS' category denotes SNVs that are commonly identified by all three methods. The 'Cactus&ACMGA-WGS' category denotes SNVs discerned by both Cactus and ACMGA, but remain undetected by WGS. The 'Cactus&WGS-ACMGA' category denotes SNVs discerned by both ACMGA and WGS, but remain undetected by Cactus.

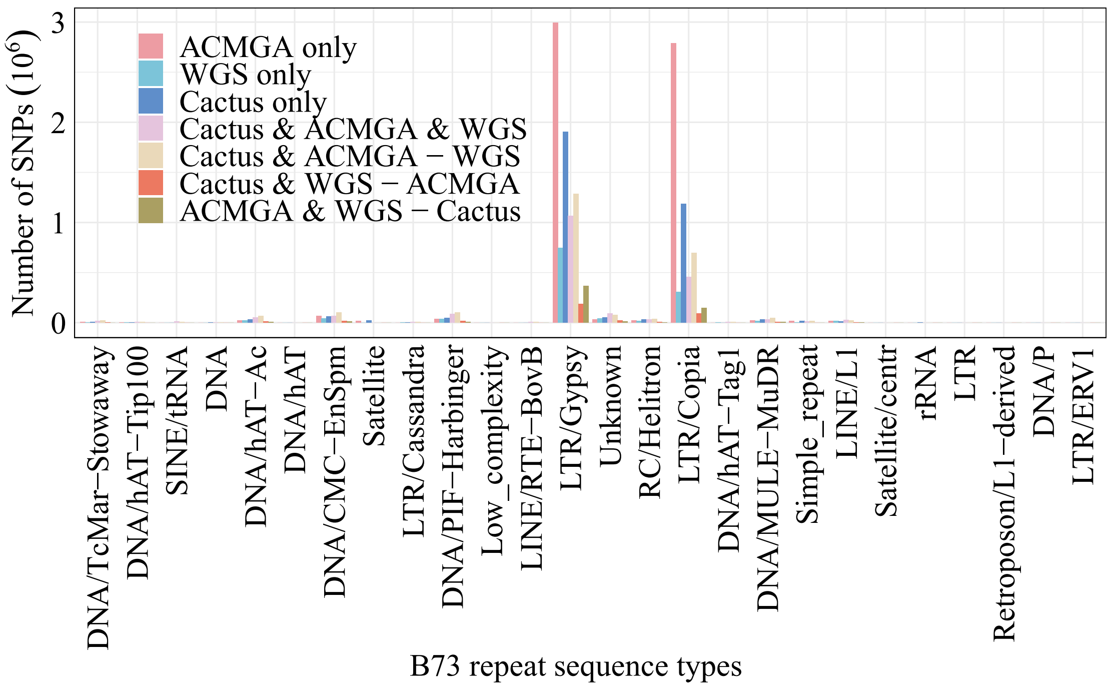

Fig. S104 The number of SNVs originating from repeated sequences between NC358 and B73 in different methods The legends that correspond to the seven independent parts of the Venn diagram in Fig. 21A. The 'ACMGA only' category denotes SNVs that are exclusively identifiable by the ACMGA, remaining undetected by alternative methodologies. The 'WGS only' category denotes SNVs that are exclusively identifiable by the WGS, remaining undetected by alternative methodologies. The 'Cactus only' category denotes SNVs that are exclusively identifiable by the Cactus, remaining undetected by alternative methodologies. The 'Cactus&ACMGA&WGS' category denotes SNVs that are commonly identified by all three methods. The 'Cactus&ACMGA-WGS' category denotes SNVs discerned by both Cactus and ACMGA, but remain undetected by WGS. The 'Cactus&WGS-ACMGA' category denotes SNVs discerned by both ACMGA and WGS, but remain undetected by Cactus.

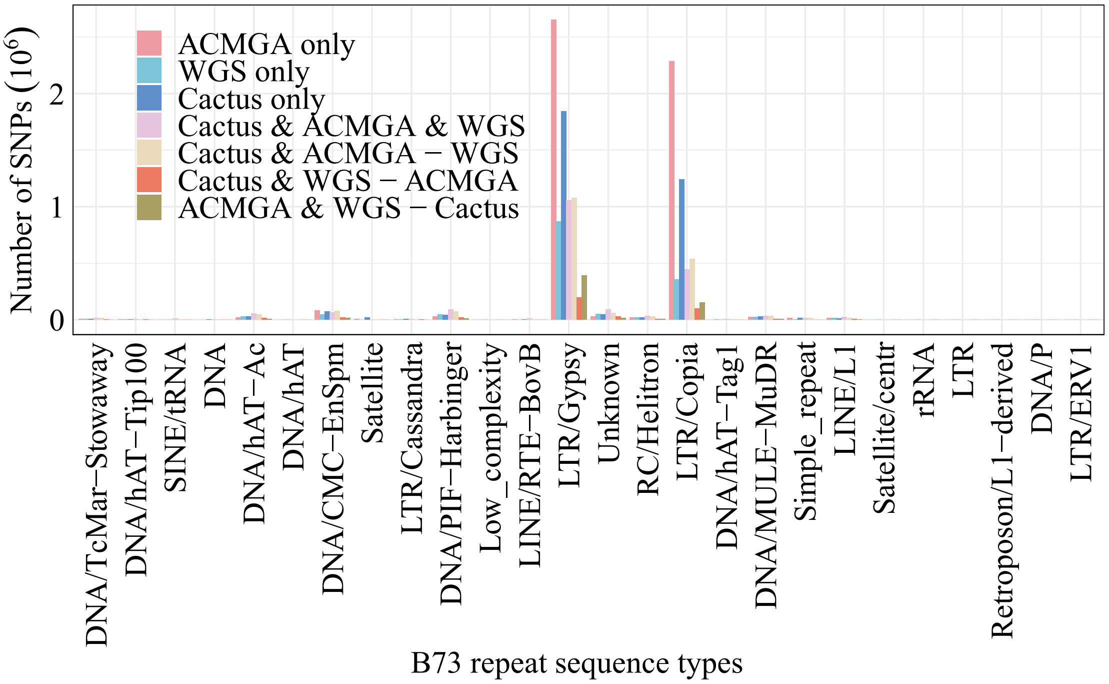

Fig. S105 The number of SNVs originating from repeated sequences between Oh43 and B73 in different methods The legends that correspond to the seven independent parts of the Venn diagram in Fig. 22A. The 'ACMGA only' category denotes SNVs that are exclusively identifiable by the ACMGA, remaining undetected by alternative methodologies. The 'WGS only' category denotes SNVs that are exclusively identifiable by the WGS, remaining undetected by alternative methodologies. The 'Cactus only' category denotes SNVs that are exclusively identifiable by the Cactus, remaining undetected by alternative methodologies. The 'Cactus&ACMGA&WGS' category denotes SNVs that are commonly identified by all three methods. The 'Cactus&ACMGA-WGS' category denotes SNVs discerned by both Cactus and ACMGA, but remain undetected by WGS. The 'Cactus&WGS-ACMGA' category denotes SNVs discerned by both ACMGA and WGS, but remain undetected by Cactus.

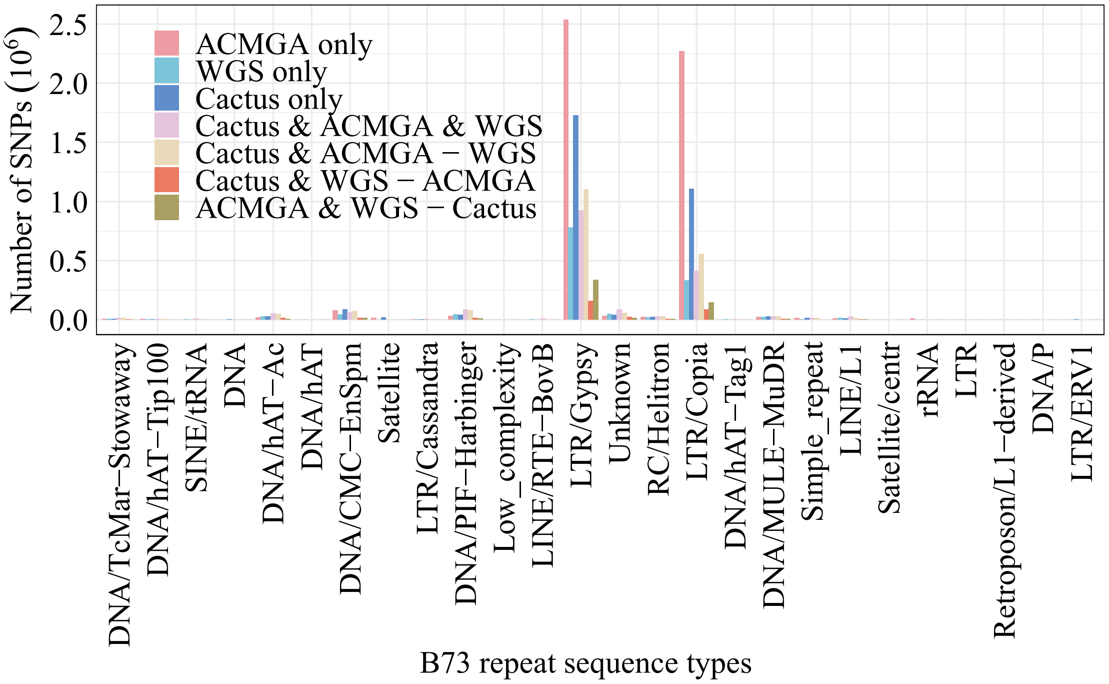

Fig. S106 The number of SNVs originating from repeated sequences between Oh7B and B73 in different methods. The legends that correspond to the seven independent parts of the Venn diagram in Fig. 23A. The 'ACMGA only' category denotes SNVs that are exclusively identifiable by the ACMGA, remaining undetected by alternative methodologies. The 'WGS only' category denotes SNVs that are exclusively identifiable by the WGS, remaining undetected by alternative methodologies. The 'Cactus only' category denotes SNVs that are exclusively identifiable by the Cactus, remaining undetected by alternative methodologies. The 'Cactus&ACMGA&WGS' category denotes SNVs that are commonly identified by all three methods. The 'Cactus&ACMGA-WGS' category denotes SNVs discerned by both Cactus and ACMGA, but remain undetected by WGS. The 'Cactus&WGS-ACMGA' category denotes SNVs discerned by both ACMGA and WGS, but remain undetected by Cactus.

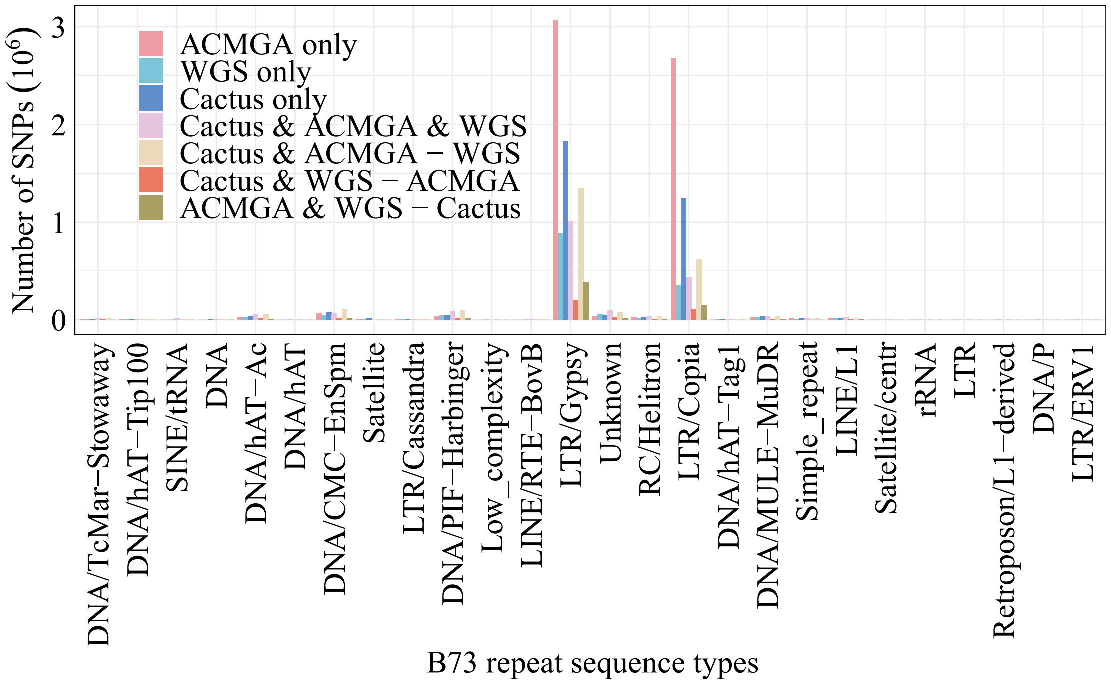

Fig. S107 The number of SNVs originating from repeated sequences between P39 and B73 in different methods. The legends that correspond to the seven independent parts of the Venn diagram in Fig. 24A. The 'ACMGA only' category denotes SNVs that are exclusively identifiable by the ACMGA, remaining undetected by alternative methodologies. The 'WGS only' category denotes SNVs that are exclusively identifiable by the WGS, remaining undetected by alternative methodologies. The 'Cactus only' category denotes SNVs that are exclusively identifiable by the Cactus, remaining undetected by alternative methodologies. The 'Cactus&ACMGA&WGS' category denotes SNVs that are commonly identified by all three methods. The 'Cactus&ACMGA-WGS' category denotes SNVs discerned by both Cactus and ACMGA, but remain undetected by WGS. The 'Cactus&WGS-ACMGA' category denotes SNVs discerned by both ACMGA and WGS, but remain undetected by Cactus.

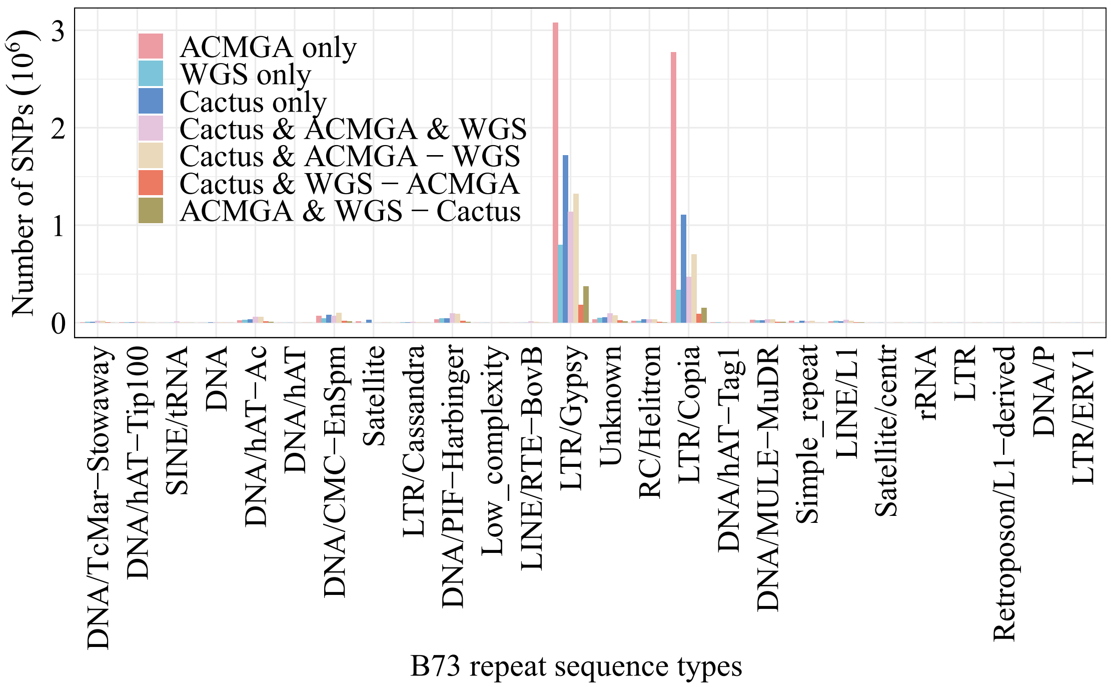

Fig. S108 The number of SNVs originating from repeated sequences between Tx303 and B73 in different methods. The legends that correspond to the seven independent parts of the Venn diagram in Fig. 25A. The ‘ACMGA only’ category denotes SNVs that are exclusively identifiable by the ACMGA, remaining undetected by alternative methodologies. The ‘WGS only’ category denotes SNVs that are exclusively identifiable by the WGS, remaining undetected by alternative methodologies. The ‘Cactus only’ category denotes SNVs that are exclusively identifiable by the Cactus, remaining undetected by alternative methodologies. The ‘Cactus&ACMGA&WGS’ category denotes SNVs that are commonly identified by all three methods. The ‘Cactus&ACMGA-WGS’ category denotes SNVs discerned by both Cactus and ACMGA, but remain undetected by WGS. The ‘Cactus&WGS-ACMGA’ category denotes SNVs discerned by both ACMGA and WGS, but remain undetected by Cactus.

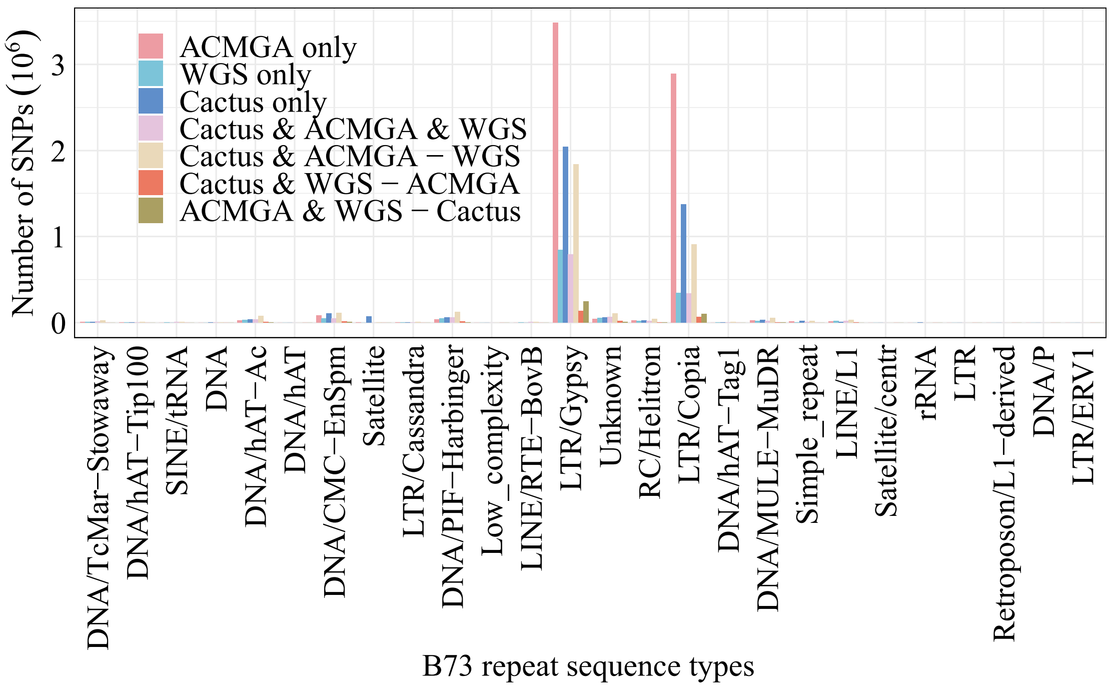

Fig. S109 The number of SNVs originating from repeated sequences between Tzi8 and B73 in different methods. The legends that correspond to the seven independent parts of the Venn diagram in Fig. 26A. The 'ACMGA only' category denotes SNVs that are exclusively identifiable by the ACMGA, remaining undetected by alternative methodologies. The 'WGS only' category denotes SNVs that are exclusively identifiable by the WGS, remaining undetected by alternative methodologies. The 'Cactus only' category denotes SNVs that are exclusively identifiable by the Cactus, remaining undetected by alternative methodologies. The 'Cactus&ACMGA&WGS' category denotes SNVs that are commonly identified by all three methods. The 'Cactus&ACMGA-WGS' category denotes SNVs discerned by both Cactus and ACMGA, but remain undetected by WGS. The 'Cactus&WGS-ACMGA' category denotes SNVs discerned by both ACMGA and WGS, but remain undetected by Cactus.

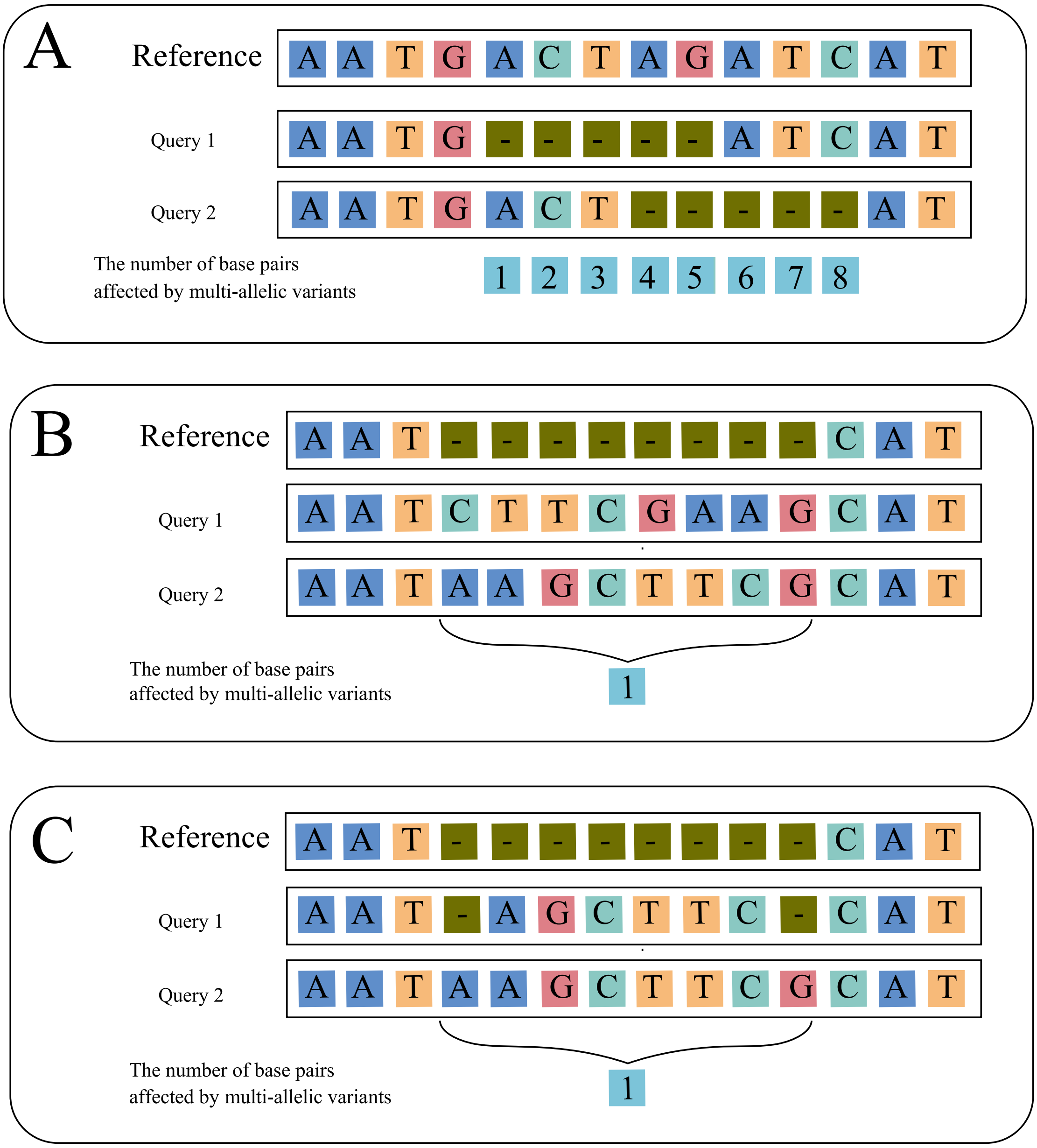

Fig. S110 Counting the number of reference genome base pairs affected by multi-allelic variants. (A) When deletion overlaps another deletion, the number of reference genome base pairs were calculated as the cumulative length covered by these overlapping deletions. **(B)** The different insertion variations of the same length, at an identical position were counted as single reference base pair affected by multi-allelic variants. **(C)** The insertions with difference lengths, while at the same position were counted as single reference base pair affected by multi-allelic variants.
